# Synthesis of Polyheterocyclic Pyrrolo[3,4-*b*]pyridin-5-ones via a One-Pot (Ugi-3CR/aza Diels-Alder/*N*-acylation/aromatization/S_N_2) Process. A Suitable Alternative towards Novel Aza-Analogues of Falipamil

**DOI:** 10.3390/molecules23040763

**Published:** 2018-03-27

**Authors:** Angel Zamudio-Medina, Ailyn N. García-González, Genesis K. Herrera-Carrillo, Daniel Zárate-Zárate, Adriana Benavides-Macías, Joaquín Tamariz, Ilich A. Ibarra, Alejandro Islas-Jácome, Eduardo González-Zamora

**Affiliations:** 1 Unidad Profesional Interdisciplinaria de Biotecnología, Instituto Politécnico Nacional, Av. Acueducto de Guadalupe S/N, Barr. La Laguna Ticomán, C.P. 07340, Del. Gustavo A Madero, Ciudad de México, Mexico; angelzamu2015@hotmail.com; 2 Departamento de Química, Universidad Autónoma Metropolitana-Iztapalapa, San Rafael Atlixco 186, Col. Vicentina, C.P. 09340, Del. Iztapalapa, Ciudad de México, Mexico; natgg@outlook.com (A.N.G.-G.); purpulove-@live.com.mx (G.K.H.-C.); danzarate_286@hotmail.com (D.Z.-Z.); 3 Escuela Nacional de Ciencias Biológicas, Instituto Politécnico Nacional, Prol. Manuel Carpio y Plan de Ayala S/N, Col. Santo Tomás, C.P. 11340, Del. Miguel Hidalgo, Ciudad de México, Mexico; a_benavides@prodigy.net.mx (A.B.-M.); jtamarizm@gmail.com (J.T.); 4 Laboratorio de Fisicoquímica y Reactividad de Superficies, Instituto de Investigaciones en Materiales, Universidad Nacional Autónoma de México, Circuito Exterior S/N, Ciudad Universitaria, C.P. 04510, Del. Coyoacán, Ciudad de México, Mexico

**Keywords:** one-pot procedures, cascade processes, multicomponent reactions, Ugi-3CR, aza Diels-Alder Cycloadditions, microwave assisted synthesis, Pyrrolo[3,4-*b*]pyridin-5-ones, Falipamil, piperazine-linker

## Abstract

We describe the one-pot synthesis of twenty polyheterocyclic pyrrolo[3,4-*b*]pyridin-5-ones *via* a cascade process (Ugi-3CR/aza Diels-Alder/*N*-acylation/aromatization) in 20 to 95% overall yields, as well as four pharmacologically promising analogues *via* an improved cascade process (Ugi-3CR/aza Diels-Alder/*N*-acylation/aromatization/S_N_2): two piperazine-linked pyrrolo[3,4-*b*]pyridin-5-ones in 33 and 34%, and a couple of Falipamil aza-analogues in 30 and 35% overall yields. It is worth highlighting the good substrate scope found, because final products are furnished with alkyl, aryl, and heterocyclic substituents. The use of chain-ring tautomerizable isocyanides (as key reagents for the Ugi-type three component reaction) allowed for a rapid and efficient assembly of the polysubstituted oxindoles, which were used in situ toward the complex products, conferring features like robustness, sustainability, and the one-pot approach to this synthetic methodology.

## 1. Introduction

**Figure 1:**
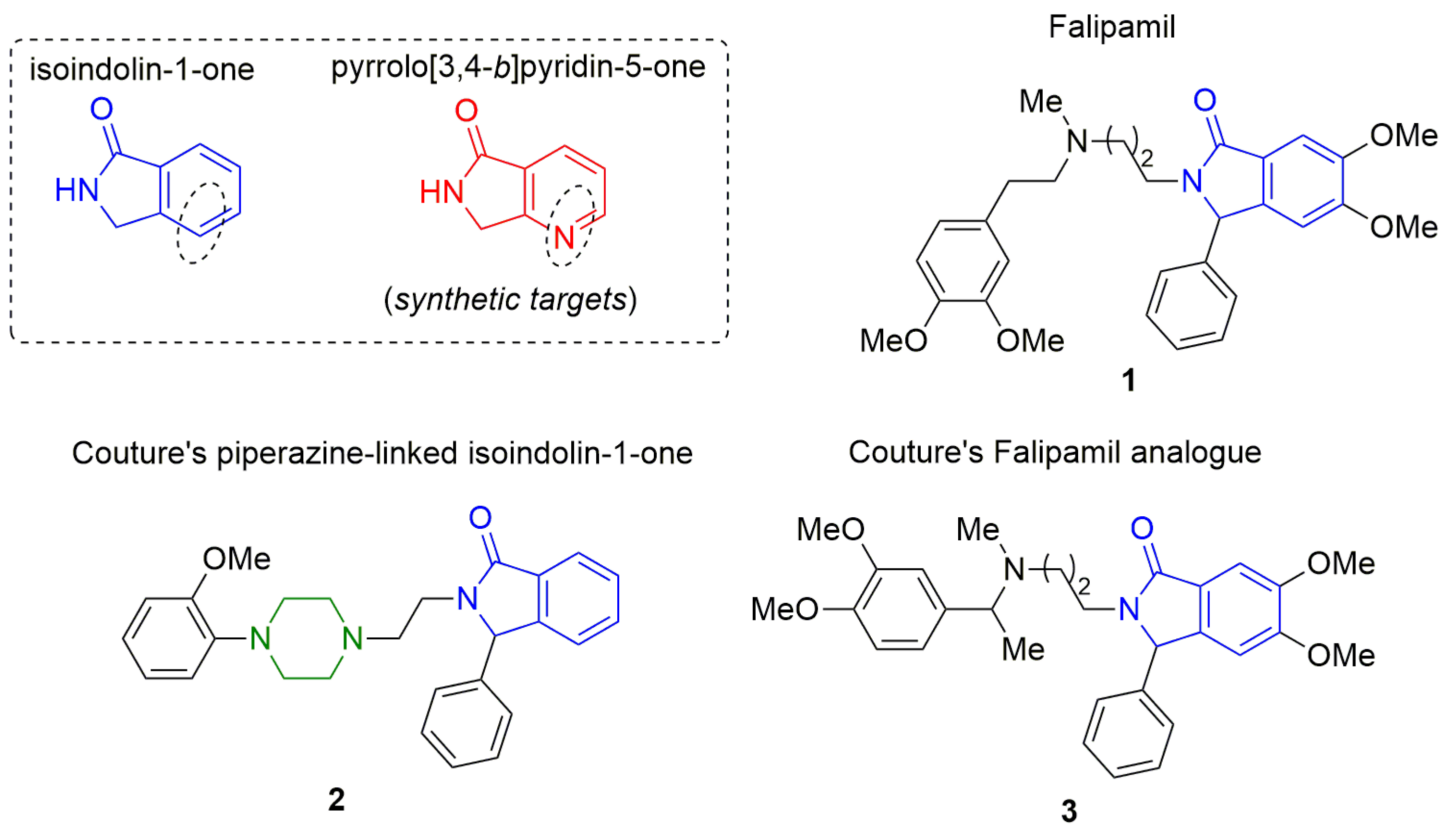
Isoindolin-1-one and one of its aza-analogues (pyrrolo[3,4-*b*]pyridin-5-one).

Falipamil (**1**) is a calcium-channel-blocker of high interest in the medicinal chemistry due to its bradycardic, vagolytic, and anti-ischemic properties [1,2,3] (Figure 1). From a structural point of view, the compound **1** is an isoindolin-1-one, a benzo[*d*]fused heterocycle found in many natural and synthetic products exhibiting biological activity [4]. Moreover, the pyrrolo[3,4-*b*]pyridin-5-one is a pyrrolo[*b*]fused polyheterocyclic system that can be considered an aza-analogue of the isoindolin-1-one core, because the fused benzene is replaced by a pyridine. Similarly, various bioactive products contain the pyrrolo[3,4-*b*]pyridin-5-one system within their structures. Besides, piperazine is a common structural motif in bioactive products, and thus it is considered a privileged linker in medicinal chemistry [5]. For example, Kung et al. described that various compounds containing the isoindolin-1-one system that are piperazine-linked to other heterocycles exhibit strong binding affinity to the 5-hydroxytryptamine 1A receptor [6]. In the same way, Couture et al. synthesized various piperazine-linked isoindolin-1-ones like the compound **2** (Figure 1), bearing in mind its possible use for further SAR studies [7]. It is worthy to note that in the same work, Falipamil (**1**) and various related analogues, such as the product **3** (Figure 1), were synthesized successfully via a stepwise (multistep) strategy. Indeed, almost all reports describing the synthesis of isoindolin-1-ones and pyrrolo[3,4-*b*]pyridin-5-ones, including those piperazine-linked to other heterocyclic systems, have been synthesized using stepwise methodologies [8].

We have investigated new one-pot synthetic strategies, mainly based on multicomponent reactions (MCR) to construct novel polyheterocyclic compounds with potential applications in different fields of knowledge such as agrochemistry, materials and polymers science, optics, and medicinal chemistry [9]. In this context, we have reported some one-pot syntheses on a series of novel, fused, polyheterocyclic pyrrolo[3,4-*b*]pyridin-5-one-based compounds via an Ugi-3CR/aza Diels-Alder/*N*-acylation/aromatization cascade sequence combined with further cyclization processes like free-radical mediated [10], Pummerer [11], Pictet-Spengler [12], and Pomeranz-Fritsch [13]. In the same way, we developed an oxidative [14] and a repetitive version [15] of this robust one-pot cascade process (Ugi-3CR/aza Diels-Alder/*N*-acylation/aromatization). Additionally, by using this methodology (one-pot approach), diverse aza-analogues of natural products, such as (±)-Nuevamine, (±)-Lennoxamine, and Magallanesine, were synthesized successfully [13]. On the other hand, to the best of our knowledge, the synthesis of Falipamil aza-analogues (or its piperazine-linked analogues) has not been previously reported either via stepwise, or one-pot cascade, or via Multicomponent Reactions (MCR)-based strategies.

Thus, it can be found in the literature of our own reports [10,11,12,13,14,15], which are previous works to the present one, and in the above-mentioned methodology from the Couture’s group [7], which is a close work to the present one, in which Falipamil (**1**) and some of its analogues (**2**–**3**) are synthesized efficiently, but via a stepwise strategy, resulting in larger times and generally using harsh conditions. Moreover, pioneering works from Bienaymé and Zhu [16,17] allowed the synthesis of pyrrolo[3,4-*b*]pyridin-5-ones via a cascade process (Ugi-3CR/aza Diels-Alder/*N*-acylation/aromatization) in good yields, short reaction times, and under relatively milder conditions with respect to stepwise methods. Thus, the main hypothesis behind the present work is that novel polyheterocyclic pyrrolo[3,4-*b*]pyridin-5-ones, including some piperazine-linked and Falipamil aza-analogues, can be synthesized efficiently via a cascade process (Ugi-3CR/aza Diels-Alder/*N*-acylation/aromatization) followed by a S_N_2 reaction under a new and robust MW-assisted one-pot process. Hence, this synthetic approach is in line with the idea extracted from the remarkable work by Danishefsky et al. “the development of novel synthetic strategies toward analogues of natural products with potential application in medicinal chemistry will always be worthy to be investigated” [18].

## 2. Results and Discussion

**Scheme 1:**
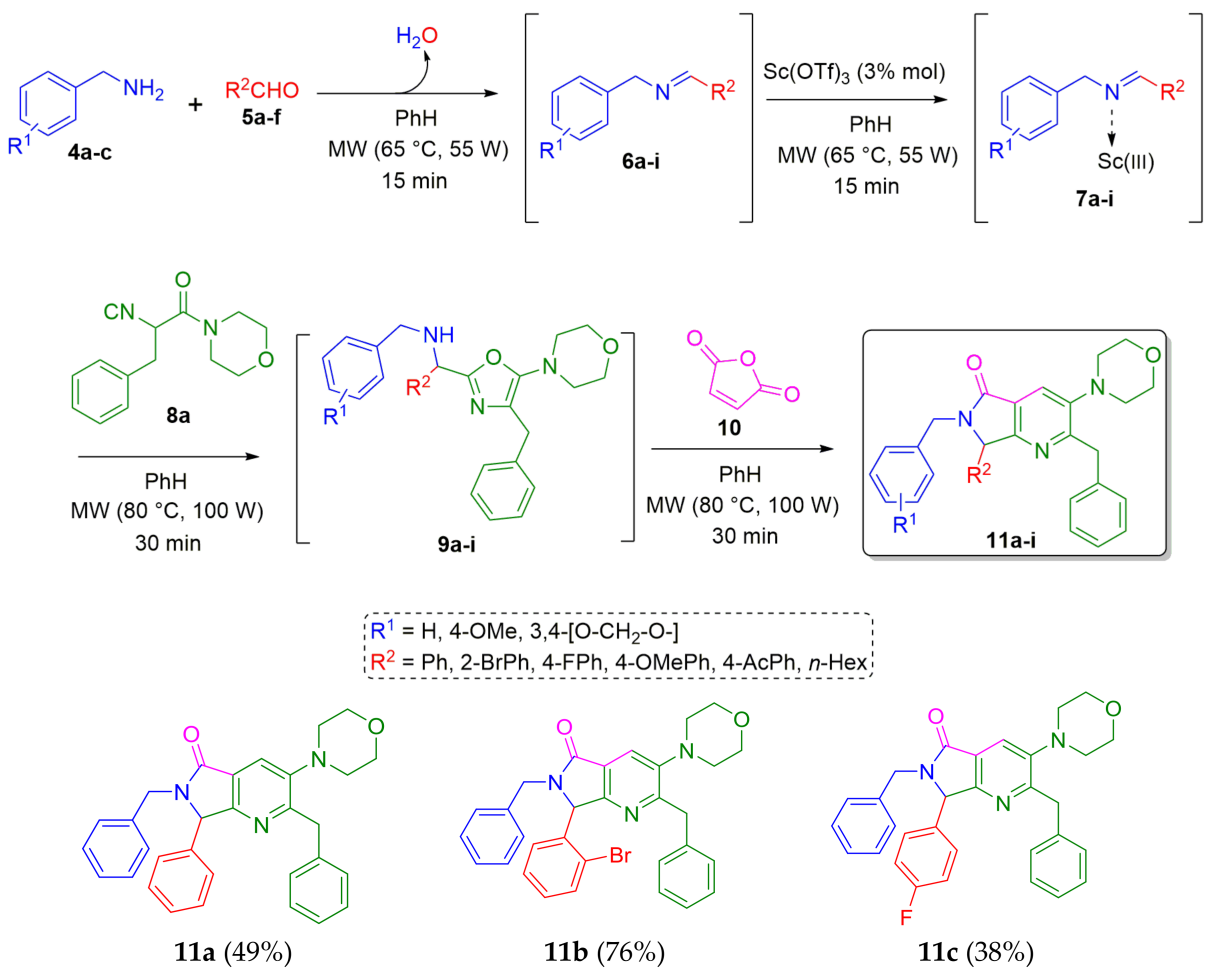

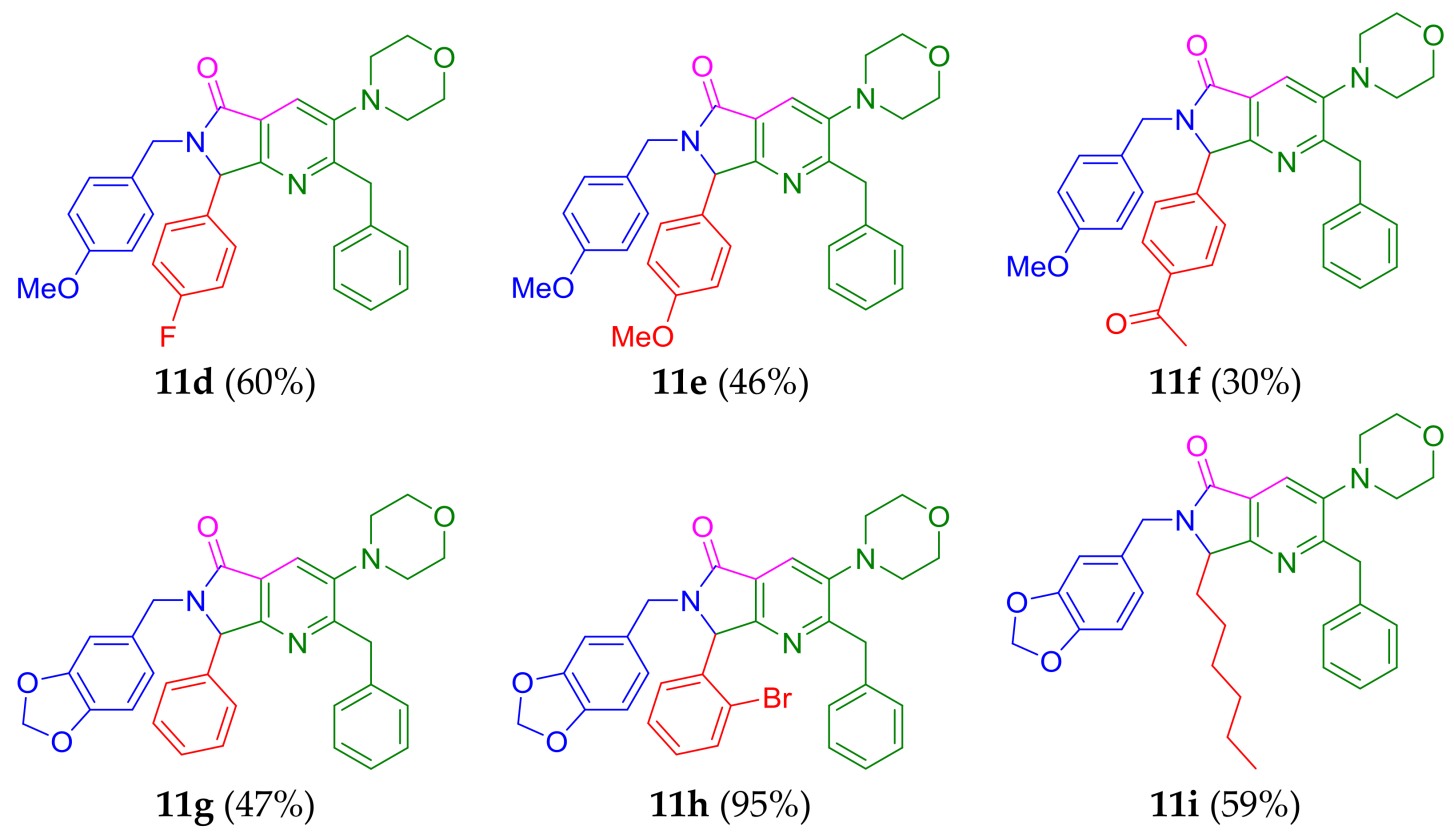
Synthesis of pyrrolo[3,4-*b*]pyridin-5-ones via a cascade process (Ugi-3CR/aza Diels-Alder/*N*-acylation/aromatization).

The synthesis of the desired pyrrolo[3,4-*b*]pyridin-5-ones **11a**–**i** was performed using our previously optimized conditions [10]: scandium(III) triflate as the Lewis-acid catalyst (3% mol) [19], microwaves (MW) as heat source to reduce reaction times [20], and benzene as the solvent. Thus, 1.0 equiv. of the corresponding benzylamines **4a**–**c** (R^1^ = **a** H, **b** 4-OMe, **c** 3,4-[-O-CH_2_-O-]) were combined with 1.0 equiv. of the corresponding aldehydes **5a**–**f** (R^2^ = **a** Ph, **b** 2-BrPh, **c** 4-FPh, **d** 4-OMePh, **e** 4-AcPh, **f** *n*-Hex) in benzene [0.5 M] at 65 °C under MW heating conditions (55 W) to give the Schiff bases **6a**–**i**, which were activated in situ by scandium (III) triflate as Lewis-acid catalyst to produce the iminium-like intermediates **7a**–**i**. Then, 1.2 equiv. of the isocyanide **8a** (prepared in three steps from the racemic phenylalanine [21]) were added sequentially to provide the corresponding 5-aminooxazoles **9a**–**i** in quantitative yields. These intermediates used as in situ were prepared to access to the polyheterocycles **11a**–**i** in one-pot manner. Consequently, when intermediates **9a**–**i** were detected by TLC (by typical features such as R*_f_* and spot nature [22]), 1.4 equiv. of the maleic anhydride (**10**) [23] was added to give the desired products **11a-i** via a cascade triple process: *N*-acylation/aza Diels-Alder cycloaddition/aromatization (decarboxylation-dehydration) (Scheme 1).

Although some yields of products **11** were moderate, these results can be considered satisfactory due to the molecular complexity of final products and the fact that they were synthesized in one-pot manner. This methodology was also demonstrated to have highly atomic economy, since various new C-C and C-N bonds were created, and only two molecules of water and one of carbon dioxide were lost in all the process. In addition, compounds **11c** and **11d** contain a fluorine atom in their structures. It is well known that the incorporation of fluorine atoms into the structure of potentially bioactive molecules often enhances their pharmacokinetic properties such as lipophilicity, oral bioavailability, and metabolic resistance [24]. Interestingly, the product **11h** was synthesized in 95% yield (the highest among all the products), probably due to the high nucleophilicity of the piperonyl amine (doubly activated by the di-oxamethylene group in its 3,4-positions) combined with the highly activated 2-bromobenzaldehyde.

**Scheme 2:**
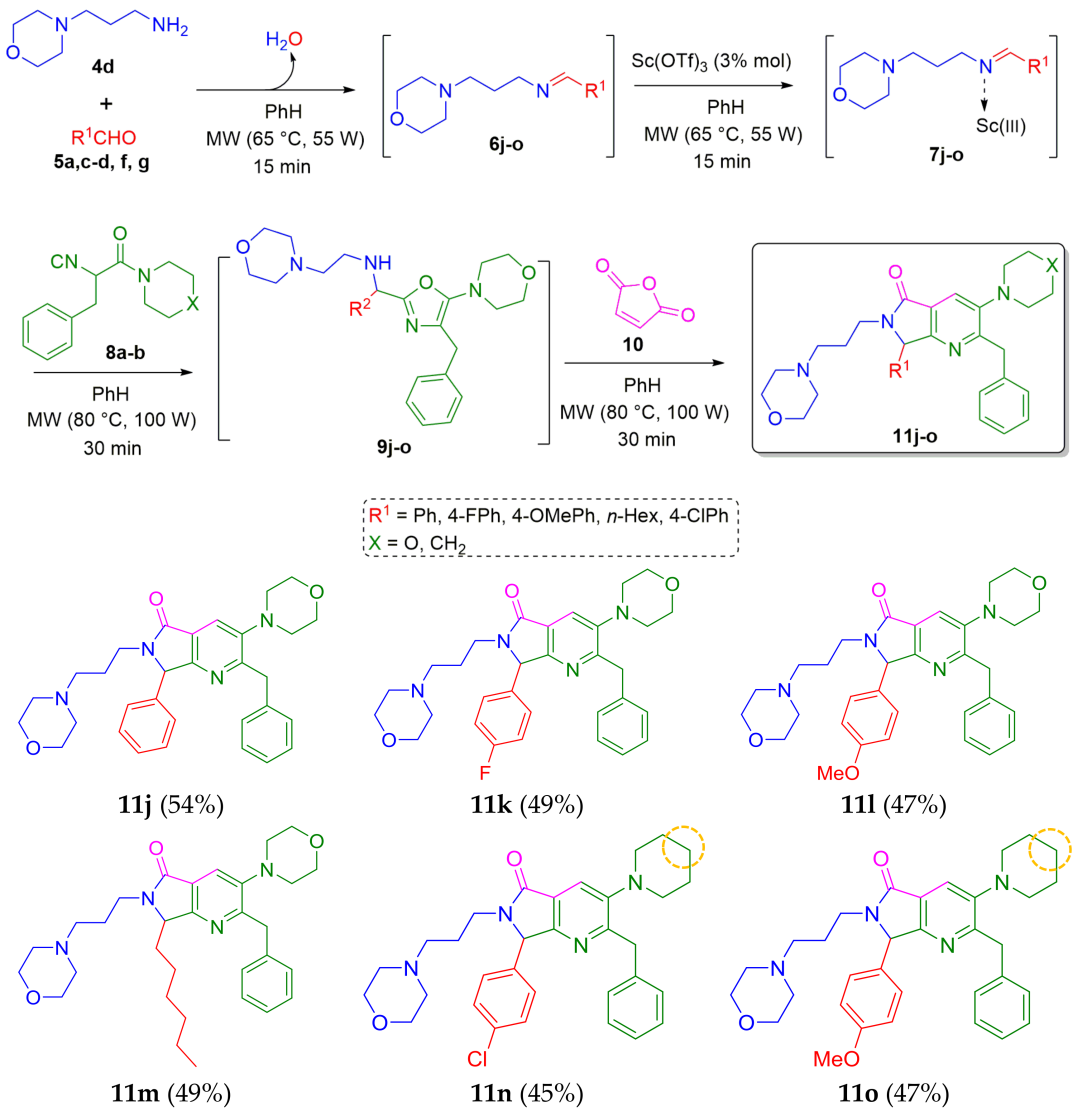
Synthesis of morpholine-based polyheterocyclic pyrrolo[3,4-*b*]pyridin-5-ones via a cascade process (Ugi-3CR/aza Diels-Alder/*N*-acylation/aromatization).

Continuing with our efforts to gain substrate scope, we synthesized the series of morpholine-containing analogues **11j**–**o**. Thus, the 3-morpholinopropan-1-amine (**4d**) was combined sequentially with the aldehydes **5a,c**–**d,f,g** (R^1^ = **a** Ph, **c** 4-FPh, **d** 4-OMePh, **f** *n*-Hex, **g** 4-ClPh), isocyanides **8a**–**b** (X = **a** O, **b** CH_2_) and maleic anhydride **10** via an Ugi-3CR/aza Diels-Alder/*N*-acylation/aromatization cascade process (Scheme 2).

It is worth highlighting the structural versatility of the products, because they are provided with alkyl, aryl, and heterocyclic substituents. The use of the piperidine-containing isocyanide **8b** to synthesize the analogues **11n** (45%) and **11o** (47%) appears not to alter the efficiency of the reactions, since their yields remained close to those of the other analogues **11j–m** (47–54%).

**Scheme 3:**
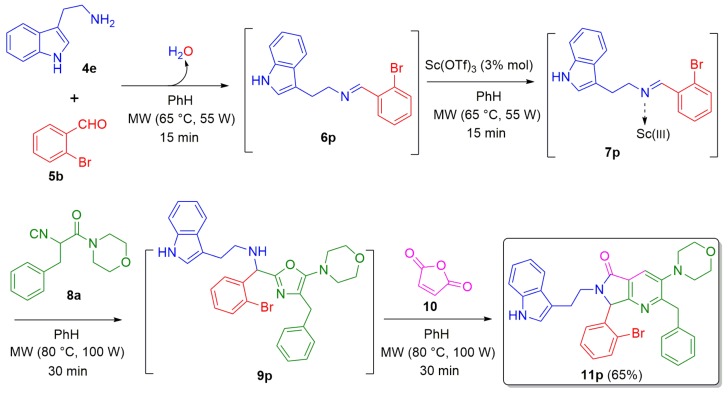
Synthesis of a tryptamine-based polyheterocyclic pyrrolo[3,4-*b*]pyridin-5-one via a cascade process (Ugi-3CR/aza Diels-Alder/*N*-acylation/aromatization).

A further example (in which the amino component of the MCR is modified) was performed by utilizing the tryptamine (**4e**) to prepare the polyheterocyclic analogue **11p** in 65% yield. Thus, the tryptamine (**4e**) was combined sequentially with 2-bromobenzaldehyde (**5b**), isocyanide **8a,** and maleic anhydride (**10**) via an Ugi-3CR/aza Diels-Alder/*N*-acylation/aromatization cascade process (Scheme 3).

**Scheme 4:**
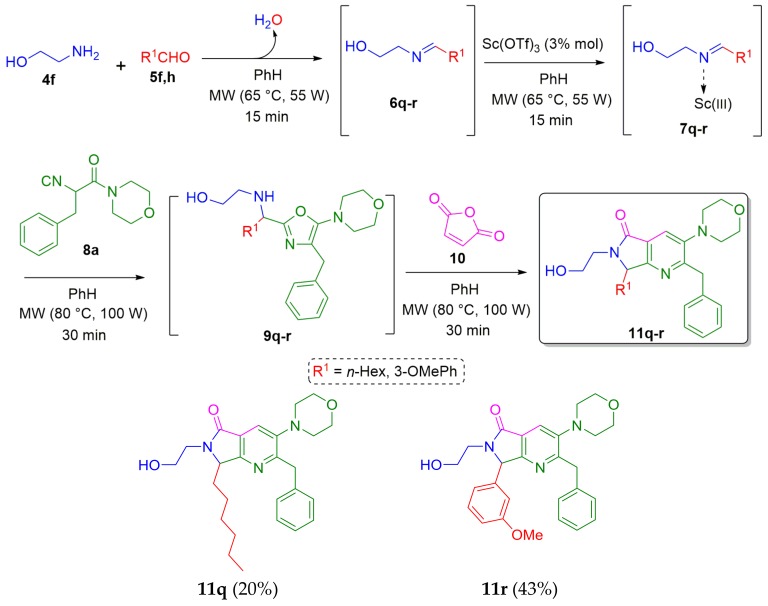
Synthesis of *N*-alcohol-functionalized pyrrolo[3,4-*b*]pyridin-5-ones via a cascade process (Ugi-3CR/aza Diels-Alder/*N*-acylation/aromatization).

Having in mind the idea of functionalizing the amine moiety of the pyrrolo[3,4-*b*]pyridin-5-ones to construct more complex polyheterocycles, the alcohol-containing analogues **11q**–**r** were synthesized in 43 and 20 % yields, respectively. Thus, 2-aminoethan-1-ol (**4f**) was combined sequentially with the corresponding aldehydes **5f**,**h** (R^1^ = **f** *n*-Hex, **h** 3-OMePh), the isocyanide **8a** and maleic anhydride (**10**), using the previously detailed methodology to afford the expected products **11q**–**r** (Scheme 4).

**Scheme 5:**
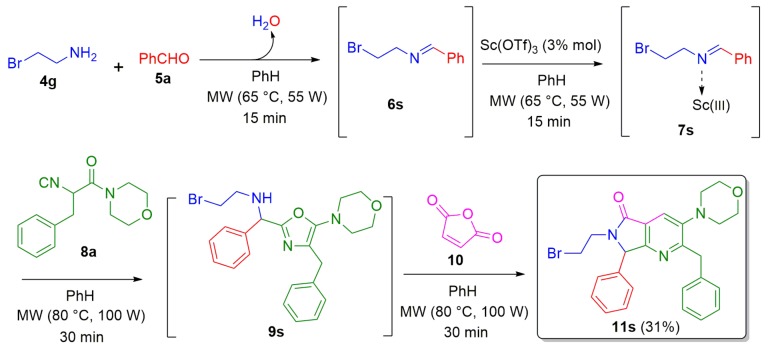
Synthesis of the bromide-functionalized pyrrolo[3,4-*b*]pyridin-5-one via a cascade process (Ugi-3CR/aza Diels-Alder/*N*-acylation/aromatization).

Then, with the aim of evaluating the introduction of an additional step in the one-pot process, a further S_N_2 reaction was adapted to our cascade process. The first objective was to prepare the bromine-containing analogue **11s** as a common precursor to introduce a variety of amines through a sequential S_N_2 reaction. Thus, 2-bromoethan-1-amine **4g** was used as starting material, which reacted sequentially with the benzaldehyde (**5a**), isocyanide **8a,** and maleic anhydride (**10**) to furnish the product **11s** in 31% yield (Scheme 5).

**Scheme 6:**
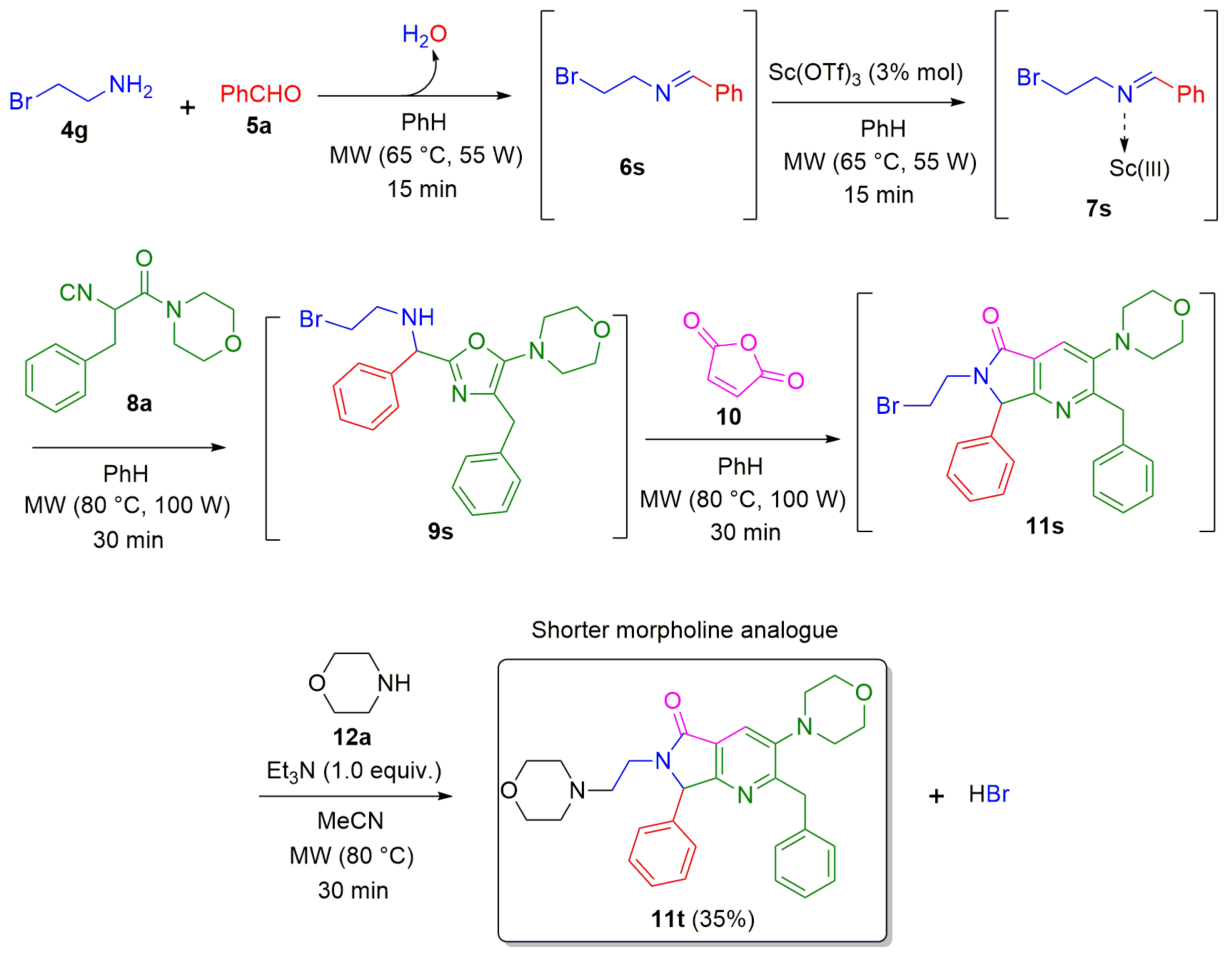
Synthesis of a morpholine-containing pyrrolo[3,4-*b*]pyridin-5-one via an improved one-pot process (Ugi-3CR/aza Diels-Alder/*N*-acylation/aromatization/S_N_2).

Subsequently, the availability of the S_N_2 reaction was tested by treatment of **11s** with a mixture of morpholine (**12a**) and triethylamine in acetonitrile as the solvent using MW as heat source (80 °C) for 30 minutes to synthesize the morpholine-containing polyheterocyclic pyrrolo[3,4-*b*]pyridin-5-one **11t** in 35% yield (Scheme 6). Since the chain length between the two heterocyclic systems was shortened by one methylene, the product **11t** is a shorter analogue of the morpholine-containing polyheterocyclic pyrrolo[3,4-*b*]pyridin-5-ones **11j**–**o** (Scheme 2).

**Scheme 7:**
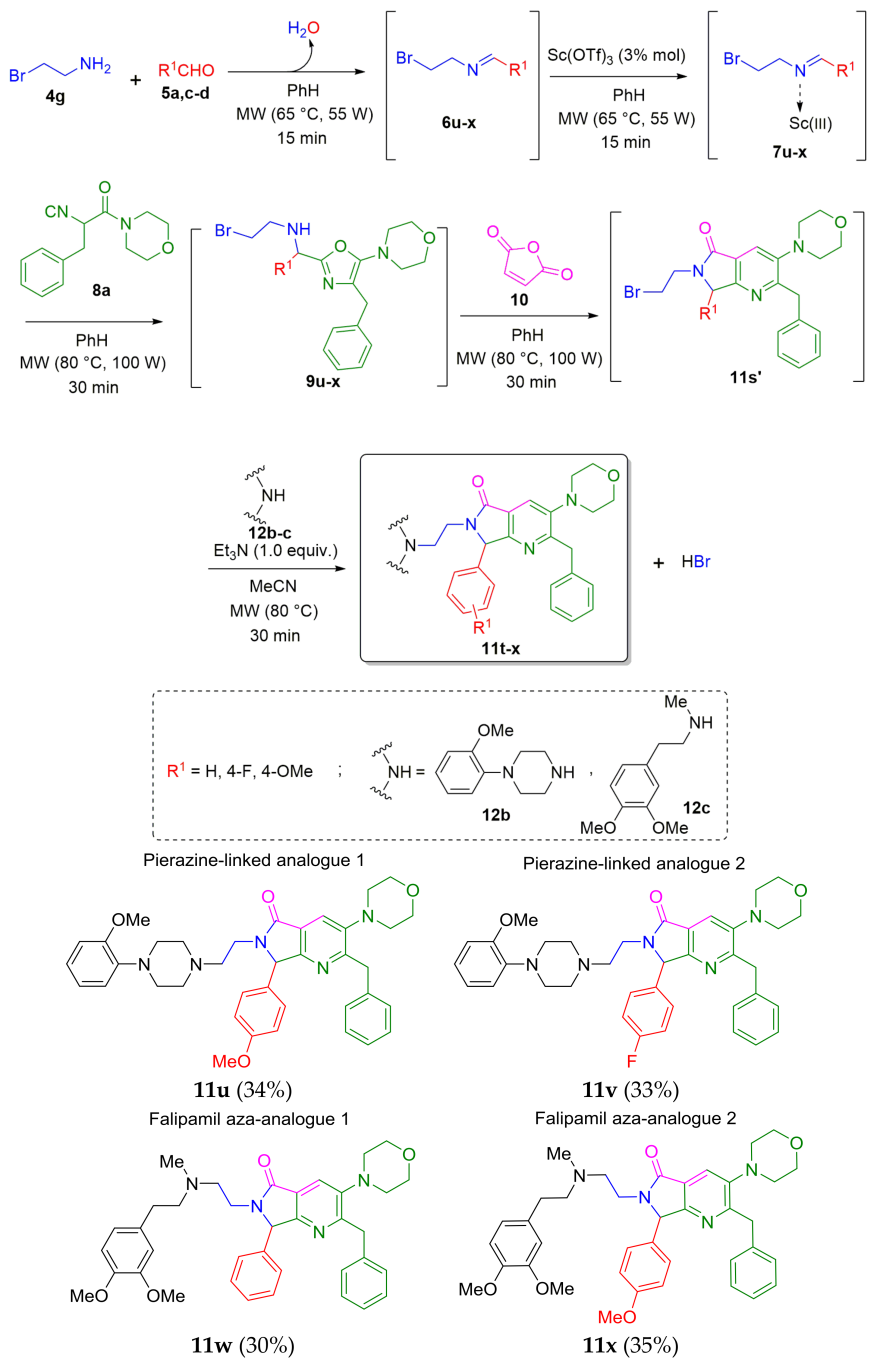
Synthesis of polyheterocyclic pyrrolo[3,4-*b*]pyridin-5-ones via an improved one-pot process (Ugi-3CR/aza Diels-Alder/*N*-acylation/aromatization/S_N_2).

Following the proposed cascade process, the more complex polyheterocyclic pyrrolo[3,4-*b*]pyridin-5-ones **11u**–**x** were synthesized from **11s** or from other of its in situ prepared bromine-containing analogues **11s**’. Then, strongly inspired by the work from Couture et al. [7], we synthesized the pair of piperazine-linked analogues **11u**–**v** in 34 and 33% yields, respectively, through the same one-pot procedure. It is noteworthy that the secondary amine used for the S_N_2 reaction (last step) was 1-(2-methoxyphenyl)piperazine (**12b**), which contains one of the most valued linkers in medicinal chemistry (piperazine). Finally, we synthesized the Falipamil aza-analogues **11w**–**x** in 30 and 35% yields, respectively, by following the procedure of preparation of the analogues **11u**–**v**. The secondary amine used for both derivatives was *N*-methyl-3,4,-dimethoxyphenethylamine (**12c**) (Scheme 7). It is worthy to note that the products **11w**–**x** present morpholine and benzyl groups attached to the pyrrolo[3,4-*b*]pyridin-5-one scaffold, which may be interpreted as a little loss of structural analogy to falipamil. However, both morpholine [25,26] and benzyl [27] are substituents that are relatively easy to remove from aromatic rings just to recover certain analogy to falipamil.

All the products herein reported **11a**–**x** were fully characterized using IR, NMR, and HRMS or EA techniques (See the Supplementary Materials for further details). Thus, the compound **11a** was selected to discuss briefly the ^1^H and ^13^C-NMR spectra. The pyridine proton (position 4) appears at 7.97 ppm due to the mesomeric effect coming from the *N*-sp^2^. Besides, the CH in which the three components of the MCR converge has a peak value of 5.30 ppm. As seen, this proton is alkylic. However, it belongs to a pyrrolidinone system. With respect to ^13^C, there are two key peaks worth highlighting. The first one is the peak at 167.1 ppm, which belongs to a γ-lactam-carbonyl carbon. The second one is just the CH in which all the reagents converge. That peak appears at 64.5 ppm, a non-typic shift for an alkylic carbon atom. It is important to mention that despite several attempts to obtain adequate crystals for X-ray analysis for at least one product were conducted; these were unsuccessful.

**Scheme 8:**
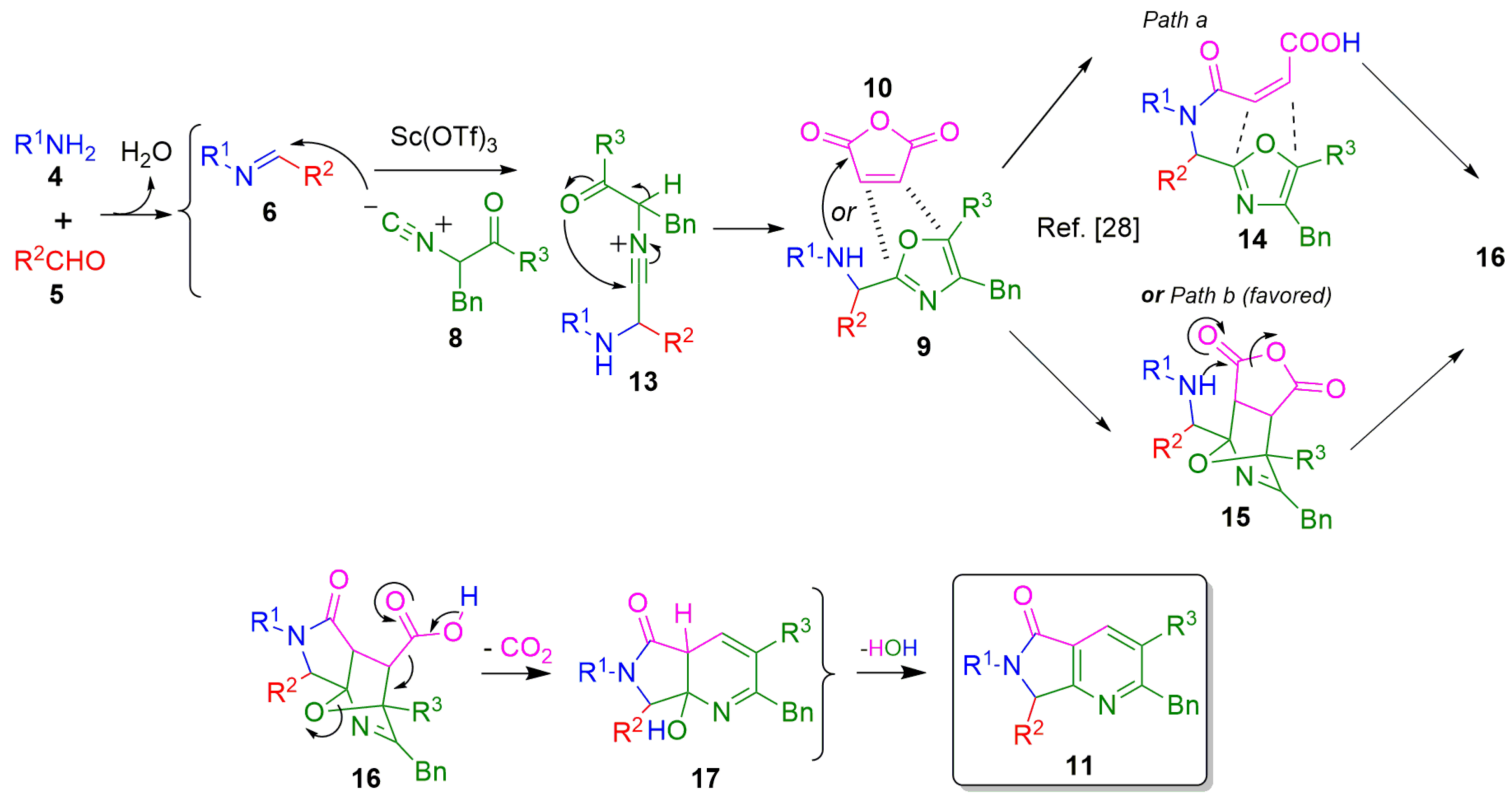
Plausible reaction mechanism (adapted from Ref. [9] with permission from the Royal Society of Chemistry).

A plausible reaction mechanism is depicted in Scheme 8, which is supported on computational calculations previously reported [28]. Thus, a condensation between amines **4** and the corresponding aldehydes **5** occurs to give the Schiff bases **6**, which are nucleophilically attacked by the respective isocyanides **8** to afford the nitrilium ions **13**. The later undergo a chain-ring tautomerization to give the 5-aminooxazoles **9** as products of the Ugi-3CR, which react with maleic anhydride (**10**) in two possible pathways: (a) intermolecular *N*-acylation/intramolecular aza Diels-Alder cycloaddition (through **14**) or (b) intermolecular aza Diels-Alder cycloaddition/intramolecular *N*-acylation (through **15**) to provide the *O*-bridged intermediate **16**. The latter undergoes a decarboxylation to give **17**, followed by a dehydration to provide the pyrrolo[3,4-*b*]pyridin-5-ones **11** (Scheme 6). It is worth noting that a close DFT-based study was reported recently by Gámez-Montaño et al. to support a plausible reaction mechanism for the construction of the isoinsolin-1-one core via an Ugi-type reaction [29]. Besides, as was discussed, the pyrrolo[3,4-*b*]pyridin-5-one is the ‘aza-version’ of the isoinsolin-1-one core.

## 3. Experimental Section

### 3.1. General Information, Instrumentation, Software, and Chemicals

^1^H and ^13^C Nuclear Magnetic Resonance (NMR) spectra were acquired on a Bruker Advance III spectrometer (500 MHz, Fällande, Uster, Switzerland). The solvent used for NMR samples was deuterated chloroform (CDCl_3_). Chemical shifts are reported in parts per million (δ/ppm). Coupling constants are reported in Hertz (*J*/Hz). Internal reference for NMR spectra was tetramethylsilane (TMS) at 0.00 ppm. Multiplicities of the signals are reported using the standard abbreviations: singlet (s), doublet (d), triplet (t), quartet (q), and multiplet (m). NMR spectra were analyzed using the MestreNova software (Ver. 10.0.1–14719). Infrared (IR) spectra were acquired on a Perkin Elmer GX spectrometer (Norwalk, CT, USA) using the Attenuated Total Reflectance (ATR) method. The absorbance peaks are reported in reciprocal centimeters (υ_max_/cm^−1^). IR spectra were analyzed using the Report Builder software (Ver. 2.01). Elemental analyses (CHNS) were determined in a Perkin Elmer 2400 Series II Analyzer (Norwalk, CT, USA). High Resolution Mass Spectroscopy (HRMS) spectra were acquired on a Jeol JMS–GC Mate II spectrometer (Akishima, Tokyo, Japan). HRMS samples were injected directly (direct probe) and analyzed by the Electron Impact (EI) method. HRMS spectra were analyzed using the Jeol-data analysis software. Melting points were determined on a Fisher-Johns apparatus (Suwanee, GA, USA) and are uncorrected. Microwave assisted reactions were performed in closed vessel mode on a CEM Discover MW-reactor (Matthews, NC, USA). Reaction progress was monitored by Thin Layer Chromatography (TLC) on precoated plates with Kieselgel 60 (F254), and the spots were visualized under Ultraviolet (UV) light (254 or 365 nm). Flash columns packed with silica-gel Merck 60 (230–400 nm) and glass preparative plates (20 × 20 cm) coated with Kieselgel 60 (F254) doped with UV indicator were used to purify the products. Mixtures in different proportions (*v*/*v*) of hexanes (Hex) with ethyl acetate (EtOAc) or ethyl acetate (EtOAc) with ethanol (EtOH) were used to run TLC, silica-gel columns, preparative plates, and to measure the Retention Factors (R*_f_*) (using the same mobile phase for all these experiments per product). All starting materials and solvents were used as received without further purification, distillation, or dehydration. Chemical structures were drawn using the ChemBioDraw software (Ver. 13.0.2.3020). The purity for all synthesized products (up to 98%) was assessed by NMR.

### 3.2. Synthesis and Characterization of the Polysubstituted Pyrrolo[3,4-*b*]pyridin-5-ones **11a**–**s**

General procedure 1 (GP-1): The corresponding amines (0.1 mmol, 1.0 equiv.) and the corresponding aldehydes (1.0 equiv.) were placed in a 10 mL sealed CEM Discover microwave reaction tube and diluted in benzene [0.5 M]. The mixture was stirred and irradiated (MW, 65 °C, 55 W) for 15 min, and then Sc(OTf)_3_ (0.03 equiv.) was added. The mixture was stirred and irradiated (MW, 65 °C, 55 W) for 15 min, and then the corresponding isocyanides (1.2 equiv.) were added. The mixture was stirred and irradiated (MW, 80 °C, 100 W) for 30 min, and then maleic anhydride (1.4 equiv.) was added. Finally, the new reaction mixture was stirred and irradiated (MW, 80 °C, 100 W) for 30 min. Then, the solvent was removed to dryness under vacuum. The crude was diluted in dichloromethane (5.0 mL), washed with a concentrated aqueous solution of NaHCO_3_ (3 × 25 mL), and then washed with brine (3 × 25 mL). The organic layer was dried using anhydrous Na_2_SO_4_ and then filtered over a celite pad. The solvent was removed to dryness under vacuum. The residue was purified immediately using a silica-gel column chromatography followed by preparative TLC using mixtures of Hex–EtOAc or EtOAc-EtOH (*v*/*v*) in different proportions as mobile phase to afford the corresponding polyheterocyclic pyrrolo[3,4-*b*]pyridin-5-ones **11a**–**s**.

#### 3.2.1. 2,6-Dibenzyl-3-morpholino-7-phenyl-6,7-dihydro-5*H*-pyrrolo[3,4-*b*]pyridin-5-one (**11a**)

According to GP-1, benzylamine (10.7 mg), benzaldehyde (10.2 µL), scandium (III) triflate (1.5 mg), 2-isocyano-1-morpholino-3-phenylpropan-1-one (29.3 mg), and maleic anhydride (13.7 mg) were reacted together in PhH (0.2 mL) to afford the product **11a** (23.3 mg, 49%) as a yellow solid; mp = 128–130 °C; R*_f_* = 0.54 (Hex–AcOEt = 1/1, *v*/*v*); FT–IR (ATR) υ_max_/cm^−1^ 921, 1114, 1263, 1442, 1695; ^1^H-NMR (500 MHz, CDCl_3_, 25 °C): δ 2.81–2.88 (m, 4H), 3.79–3.84 (m, 5H), 4.19 (d, *J* = 13.9 Hz, 1H), 4.31 (d, *J* = 13.9 Hz, 1H), 5.30 (s, 1H), 5.45 (d, *J* = 14.8 Hz, 1H), 7.13–7.18 (m, 7H), 7.22–7.23 (m, 2H), 7.29–7.34 (m, 3H), 7.38–7.41 (m, 3H), 7.97 (s, 1H); ^13^C-NMR (126 MHz, CDCl_3_, 25 °C): δ 40.0 (CH_2_), 43.9 (CH_2_), 53.0 (CH_2_), 64.5 (CH), 67.1 (CH_2_), 123.9 (Cqar), 124.1 (CHar), 126.1 (CHar), 127.7 (CHar), 128.1 (CHar), 128.2 (CHar), 128.5 (CHar), 128.7 (CHar), 128.8 (2 CHar), 129.0 (CHar), 135.3 (Cqar), 136.8 (Cqar), 139.2 (Cqar), 147.8 (Cqar), 160.5 (Cqar), 162.1 (Cqar), 167.1 (Cq); HRMS (EI): calcd. for C_31_H_30_N_3_O_2_^+^ 476.2338, found 476.2341.

#### 3.2.2. 2,6-Dibenzyl-7-(2-bromophenyl)-3-morpholino-6,7-dihydro-5*H*-pyrrolo[3,4-*b*]pyridin-5-one **11b**

According to GP-1, benzylamine (10.7 mg), 2-bromobenzaldehyde (11.7 µL), scandium (III) triflate (1.5 mg), 2-isocyano-1-morpholino-3-phenylpropan-1-one (29.3 mg), and maleic anhydride (13.7 mg) were reacted together in PhH (0.2 mL) to afford the product **11b** (42.0 mg, 76%) as a white solid; mp = 109–111 °C; R*_f_* = 0.53 (Hex–AcOEt = 1/1, *v*/*v*); FT–IR (ATR) υ_max_/cm^−1^ 940, 1015, 1112, 1441, 1694; ^1^H-NMR (500 MHz, CDCl_3_, 25 °C): δ 2.81–2.84 (m, 4H), 3.00–3.82 (m, 4H), 3.86 (d, *J* = 14.8 Hz, 1H), 4.12 (d, *J* = 13.7 Hz, 1H), 4.27 (d, *J* = 13.7 Hz, 1H), 5.32 (d, *J* = 14.7 Hz, 1H), 5.99 (s, 1H), 6.74–6.76 (m, 1H), 7.12–7.28 (m, 12H), 7.65–7.67 (m, 1H), 7.89 (s, 1H); ^13^C-NMR (126 MHz, CDCl_3_, 25 °C): δ 39.8 (CH_2_), 44.3 (CH_2_), 53.0 (CH_2_), 63.2 (CH), 67.1 (CH_2_), 123.6 (Cqar), 123.8 (CHar), 125.6 (Cqar), 126.1 (CHar), 127.7 (CHar), 127.9 (2 CHar), 128.1 (CHar), 128.6 (CHar), 128.7 (CHar), 128.9 (CHar), 129.9 (CHar), 133.5 (CHar), 134.8 (Cqar), 136.5 (Cqar), 139.1 (Cqar), 147.7 (Cqar), 160.3 (Cqar), 162.2 (Cqar), 167.4 (Cq); Elemental analysis: calcd. for C_31_H_28_BrN_3_O_2_ C 67.15, H 5.09, N 7.58%, found C 66.65, H 5.29, N 7.34%.

#### 3.2.3. 2,6-Dibenzyl-7-(4-fluorophenyl)-3-morpholino-6,7-dihydro-5*H*-pyrrolo[3,4-*b*]pyridin-5-one **11c**

According to GP-1, benzylamine (10.7 mg), 4-fluorobenzaldehyde (10.7 µL), scandium (III) triflate (1.5 mg), 2-isocyano-1-morpholino-3-phenylpropan-1-one (29.3 mg), and maleic anhydride (13.7 mg) were reacted together in PhH (0.2 mL) to afford the product **11c** (18.8 mg, 38%) as a yellow solid; mp = 152–154 °C; R*_f_* = 0.54 (Hex–AcOEt = 1/1, *v*/*v*); FT–IR (ATR) υ_max_/cm^−1^ 920, 1114, 1220, 1441, 1696; ^1^H-NMR (500 MHz, CDCl_3_, 25 °C): δ 2.79–2.85 (m, 4H), 3.76–3.81 (m, 5H), 4.17 (d, *J* = 13.9 Hz, 1H), 4.27 (d, *J* = 13.9 Hz, 1H), 5.24 (s, 1H), 5.41 (d, *J* = 14.9 Hz, 1H), 7.05–7.17 (m, 10H), 7.24–7.31 (m, 4H), 7.92 (s,1H); ^13^C-NMR (126 MHz, CDCl_3_, 25 °C): δ 40.0 (CH_2_), 43.8 (CH_2_), 53.0 (CH_2_), 63.7 (CH), 67.1 (CH_2_), 116.0 (d, *^o^J*_CF_ = 21.0 Hz, CF), 123.7 (Cqar), 123.9 (CHar), 126.2 (CHar), 127.8 (CHar), 128.1 (CHar), 128.4 (CHar), 128.7 (CHar), 128.8 (CHar), 129.8 (d, *^m^J*_CF_ = 8.3 Hz, CF), 131.1 (d, *^p^J*_CF_ = 2.6 Hz, CF), 136.6 (Cqar), 139.1 (Cqar), 147.9 (Cqar), 160.3 (Cqar), 162.1 (Cqar), 162.8 (d, *^i^J*_CF_ = 247.5 Hz, CF), 166.9 (Cq); Elemental analysis: calcd. for C_31_H_28_FN_3_O_2_ C 75.44, H 5.72, N 8.51%, found C 75.10, H, 5.75, N 8.42%.

#### 3.2.4. 2-Benzyl-7-(4-fluorophenyl)-6-(4-methoxybenzyl)-3-morpholino-6,7-dihydro-5*H*-pyrrolo[3,4-*b*]pyridin-5-one **11d**

According to GP-1, (4-methoxyphenyl)methanamine (13.1 µL), 4-fluorobenzaldehyde (10.7 µL), scandium (III) triflate (1.5 mg), 2-isocyano-1-morpholino-3-phenylpropan-1-one (29.3 mg), and maleic anhydride (13.7 mg) were reacted together in dry PhH (0.2 mL) to afford the product **11d** (31.4 mg, 60%) as a white solid; mp = 145–147 °C; R*_f_* = 0.52 (Hex–AcOEt = 1/1, *v*/*v*); FT–IR (ATR) υ_max_/cm^−1^ 917, 1114, 1221, 1246, 1441, 1509, 1694, 2852; ^1^H-NMR (500 MHz, CDCl_3_, 25 °C): δ 2.78–2.85 (m, 4H), 3.72 (d, *J* = 14.7 Hz, 1H), 3.75 (s, 3H), 3.79 (t, *J* = 4.6 Hz, 4H), 4.16 (d, *J* = 13.9 Hz, 1H), 4.27 (d, *J* = 13.9 Hz, 1H), 5.24 (s, 1H), 5.35 (d, *J* = 14.7 Hz, 1H), 6.81–6.83 (m, 2H), 7.05–7.14 (m, 11H), 7.91 (s, 1H); ^13^C-NMR (126 MHz, CDCl_3_, 25 °C): δ 39.9 (CH_2_), 43.2 (CH_2_), 53.0 (CH_2_), 55.2 (CH_3_), 63.6 (CH), 67.1 (CH_2_), 114.1 (CHar), 115.9 (d, *^o^J*_CF_ = 21.7 Hz, CF), 123.8 (CHar), 123.9 (CHar), 126.1 (CHar), 128.1 (CHar), 128.7 (CHar), 129.8 (CHar), 131.2 (d, *^p^J*_CF_ = 2.5 Hz, CF), 139.2 (Cqar), 147.8 (Cqar), 159.2 (Cqar), 160.3 (Cqar), 162.0 (Cqar), 162.8 (d, *^i^J*_CF_ = 247.4 Hz, CF), 166.8 (Cq); Elemental analysis: calcd. for C_32_H_30_FN_3_O_3_ C 73.40, H 5.78, N 8.03%, found C 73.45, H 6.13, N 8.15%.

#### 3.2.5. 2-Benzyl-6-(4-methoxybenzyl)-7-(4-methoxyphenyl)-3-morpholino-6,7-dihydro-5*H*-pyrrolo[3,4-*b*]pyridin-5-one **11e**

According to GP-1, (4-methoxyphenyl)methanamine (13.1 µL), 4-methoxybenzaldehyde (12.2 µL), scandium (III) triflate (1.5 mg), 2-isocyano-1-morpholino-3-phenylpropan-1-one (29.3 mg), and maleic anhydride (13.7 mg) were reacted together in PhH (0.2 mL) to afford the product **11e** (24.6 mg, 46%) as a yellow solid; mp = 166–168 °C; R*_f_* = 0.42 (Hex–AcOEt = 1/1, *v*/*v*); FT–IR (ATR) υ_max_/cm^−1^ 701, 940, 1015, 1112, 1441, 1694, 2842; ^1^H-NMR (500 MHz, CDCl_3_, 25 °C): δ 2.76–2.84 (m, 4H), 3.70 (d, *J* = 14.7 Hz, 1H), 3.80–3.92 (m, 10H), 4.15 (d, *J* = 13.8 Hz, 1H), 4.29 (d, *J* = 13.8 Hz, 1H), 5.21 (s, 1H), 5.34 (d, *J* = 14.7 Hz, 1H), 6.82–6.83 (m, 2H), 6.88–6.90 (m, 2H), 7.02–7.04 (m, 2H), 7.10–7.15 (m, 7H), 7.91 (s, 1H); ^13^C-NMR (126 MHz, CDCl_3_, 25 °C): δ 40.0 (CH_2_), 43.0 (CH_2_), 53.0 (CH_2_), 55.2 (CH_3_), 55.3 (CH_3_), 63.9 (CH), 67.1 (CH_2_), 114.1 (CHar), 114.4 (CHar), 123.9 (CHar), 124.0 (Cqar), 126.1 (CHar), 127.1 (Cqar), 128.1 (CHar), 128.7 (CHar), 129.0 (Cqar), 129.3 (CHar), 129.8 (CHar), 139.3 (Cqar), 147.7 (Cqar), 159.1 (Cqar), 159.8 (Cqar), 160.8 (Cqar), 161.9 (Cqar), 166.7 (Cq); HRMS (EI): calcd. for C_33_H_34_N_3_O_4_^+^ 536.2549, found 536.2611.

#### 3.2.6. 7-(4-Acetylphenyl)-2-benzyl-6-(4-methoxybenzyl)-3-morpholino-6,7-dihydro-5*H*-pyrrolo[3,4-*b*]pyridin-5-one **11f**

According to GP-1, (4-methoxyphenyl)methanamine (13.1 µL), 4-acetylbenzaldehyde (14.8 mg), scandium (III) triflate (1.5 mg), 2-isocyano-1-morpholino-3-phenylpropan-1-one (29.3 mg), and maleic anhydride (13.7 mg) were reacted together in PhH (0.2 mL) to afford the product **11f** (16.4 mg, 30%) as a yellow solid; mp = 149–151 °C; R*_f_* = 0.34 (Hex–AcOEt = 1/1, *v*/*v*); FT–IR (ATR) υ_max_/cm^−1^ 917, 1035, 1114, 1247, 1441, 1687, 2852; ^1^H-NMR (500 MHz, CDCl_3_, 25 °C): δ 2.62 (s, 3H), 2.79–2.86 (m, 4H), 3.72 (d, *J* = 14.8 Hz, 1H), 3.78 (s, 3H), 3.79–3.82 (m, 4H), 4.12 (d, *J* = 14.0 Hz, 1H), 4.27 (d, *J* = 13.9 Hz, 1H), 5.29 (s, 1H), 5.39 (d, *J* = 14.7 Hz, 1H), 6.81–6.83 (m, 2H), 7.07–7.13 (m, 7H), 7.24–7.26 (m, 2H), 7.91 (s, 1H), 7.96–7.97 (m, 2H); ^13^C-NMR (126 MHz, CDCl_3_, 25 °C): δ 126.7 (CH_3_), 40.0 (CH_2_), 43.5 (CH_2_), 53.0 (CH_2_), 55.3 (CH_3_), 63.8 (CH), 67.1 (CH_2_), 114.2 (CHar), 123.8 (Cqar), 124.0 (CHar), 126.2 (CHar), 128.2 (2 CHar), 128.5 (Cqar), 128.7 (Cqar), 129.0 (CHar),129.8 (CHar), 137.3 (Cqar),139.1 (Cqar), 140.9 (Cqar),148.0 (Cqar), 159.2 (Cqar), 159.8 (Cqar), 162.1 (Cqar), 167.0 (Cq), 197.5 (Cq); Elemental analysis: calcd. for C_34_H_33_N_3_O_4_ C 74.57, H 6.07, N 7.67%, found C 74.55, H 6.26, N 7.49%.

#### 3.2.7. 6-(benzo[*d*][1,3]dioxol-5-ylmethyl)-2-benzyl-3-morpholino-7-phenyl-6,7-dihidro-5*H*-pyrrolo[3,4-*b*]pyridin-5-one **11g**

According to GP-1, piperonylamine (12.5 µL), benzaldehyde (10.2 µL), scandium (III) triflate (1.5 mg), 2-isocyano-1-morpholino-3-phenylpropan-1-one (29.3 mg), and maleic anhydride (13.7 mg) were reacted together in PhH (0.2 mL) to afford the product **11g** (24.4 mg, 47%) as a yellow solid; mp = 126–128 °C; R*_f_* = 0.51 (Hex–AcOEt = 1/1, *v*/*v*); FT–IR (ATR) υ_max_/cm^−1^ 927, 1036, 1114, 1243, 1442, 1694; ^1^H-NMR (500 MHz, CDCl_3_, 25 °C): δ 2.77–2.84 (m, 4H), 3.68 (d, *J* = 14.8 Hz, 1H), 3.78–3.80 (m, 4H), 4.15 (d, *J* = 13.9 Hz, 1H), 4.28 (d, *J* = 13.9 Hz, 1H), 5.27 (s, 1H), 5.32 (d, *J* = 14.7 Hz, 1H), 5.90 (d, *J* = 1.5 Hz, 1H), 5.92 (d, *J* = 1.5 Hz, 1H), 6.62 (dd, *J* = 1.5, 7.8 Hz, 1H), 6.71–6.72 (m, 2H), 7.12–7.15 (m, 7H), 7.35–7.38 (m, 3H), 7.90 (s,1H); ^13^C-NMR (126 MHz, CDCl_3_, 25 °C): δ 40.0 (CH_2_), 43.6 (CH_2_), 53.0 (CH_2_), 64.3 (CH), 67.1 (CH_2_), 101.1 (CH_2_), 108.3 (CHar), 108.9 (CHar), 121.9 (CHar), 123.8 (Cqar), 123.9 (CHar), 126.1 (CHar), 128.1 (2 CHar), 128.6 (CHar), 128.8 (CHar), 129.0 (CHar), 130.7 (Cqar), 135.3 (Cqar), 139.2 (Cqar), 147.1 (Cqar), 147.8 (Cqar), 148.0 (Cqar), 160.5 (Cqar), 162.0 (Cqar), 166.9 (Cq); HRMS (EI): calcd. for C_32_H_30_N_3_O_4_^+^ 520.2236, found 520.2208.

#### 3.2.8. 6-(Benzo[*d*][1,3]dioxol-5-ylmethyl)-2-benzyl-7-(2-bromophenyl)-3-morpholino-6,7-dihydro-5*H*-pyrrolo[3,4-*b*]pyridin-5-one **11h**

According to GP-1, piperonylamine (12.5 µL), 2-bromobenzaldehyde (11.7 µL), scandium (III) triflate (1.5 mg), 2-isocyano-1-morpholino-3-phenylpropan-1-one (29.3 mg), and maleic anhydride (13.7 mg) were reacted together in PhH (0.2 mL) to afford the product **11h** (56.7 mg, 95%) as a yellow solid; mp = 109–111 °C; R*_f_* = 0.51 (Hex–AcOEt = 1/1, *v*/*v*); FT–IR (ATR) υ_max_/cm^−1^ 701, 940, 1015, 1112, 1441, 1694; ^1^H-NMR (500 MHz, CDCl_3_, 25 °C): δ 2.83–2.85 (m, 4H), 3.79 (d, *J* = 14.7 Hz, 1H), 3.83–3.85 (m, 4H), 4.12–4.16 (m, 1H), 4.31 (d, *J* = 13.7 Hz, 1H), 5.24 (d, *J* = 14.7 Hz, 1H), 5.93 (dd, *J* = 1.5, 6.1 Hz, 2H), 6.01 (s, 1H), 6.65 (dd, *J* = 1.7, 7.9 Hz, 1H), 6.71–6.77 (m, 4H), 7.13–7.24 (m, 6H), 7.69–7.71 (m, 1H), 7.90 (s, 1H); ^13^C-NMR (126 MHz, CDCl_3_, 25 °C): δ 39.9 (CH_2_), 44.1 (CH_2_), 53.0 (CH_2_), 63.1 (CH), 67.1 (CH_2_), 101.0 (CH_2_), 108.3 (CHar), 109.1 (CHar), 122.2 (CHar), 123.6 (Cqar), 123.8 (CHar), 125.6 (Cqar), 126.1 (CHar), 127.9 (CHar), 128.0 (CHar), 128.1 (CHar), 129.0 (CHar), 129.9 (CHar), 130.3 (Cqar), 133.5 (CHar), 134.9 (Cqar), 139.1 (Cqar), 147.1 (Cqar), 147.7 (Cqar), 147.9 (Cqar), 160.3 (Cqar), 162.2 (Cqar), 167.3 (Cq); HRMS (EI): calcd. for C_32_H_29_BrN_3_O_4_^+^ 598.1341, found 598.1360.

#### 3.2.9. 6-(Benzo[*d*][1,3]dioxol-5-ylmethyl)-2-benzyl-7-hexyl-3-morpholino-6,7-dihydro-5*H*-pyrrolo[3,4-*b*]pyridin-5-one **11i**

According to GP-1, piperonylamine (12.5 µL), heptanal (14.0 µL), scandium (III) triflate (1.5 mg), 2-isocyano-1-morpholino-3-phenylpropan-1-one (29.3 mg), and maleic anhydride (13.7 mg) were reacted together in PhH (0.2 mL) to afford the product **11i** (31.3 mg, 59%) as a yellow viscous liquid; R*_f_* = 0.53 (Hex–AcOEt = 1/1, *v*/*v*); FT–IR (ATR) υ_max_/cm^−1^ 701, 928, 1038,1115, 1243, 1441, 1691; ^1^H-NMR (500 MHz, CDCl_3_, 25 °C): δ 0.72–0.78 (m, 1H), 0.85 (t, *J* = 7.16 Hz, 3H), 1.10–1.22 (m, 7H), 1.84–1.93 (m, 1H), 2.01–2.15 (m, 1H), 2.83–2.89 (m, 4H), 3.83–3.86 (m, 4H), 4.11 (d, *J* = 14.9 Hz, 1H), 4.29 (d, *J* = 14.0 Hz, 1H), 4.39–4.42 (m, 2H), 5.26 (d, *J* = 14.9 Hz, 1H), 5.94 (dd, *J* = 1.4, 3.7 Hz, 2H), 6.76 (d, *J* = 7.8 Hz, 2H), 6.78–6.82 (m, 2H), 7.16–7.19 (m, 1H), 7.23–7.28 (m, 5H), 7.88 (s, 1H); ^13^C-NMR (126 MHz, CDCl_3_, 25 °C): δ 14.0 (CH_3_), 22.3 (CH_2_), 22.5 (CH_2_), 29.1 (2 CH_2_), 31.6 (CH_2_), 40.0 (CH_2_), 43.7 (CH_2_), 53.1 (CH_2_), 59.8 (CH), 67.2 (CH_2_), 101.1 (CH_2_), 108.3 (CHar), 108.7 (CHar), 121.5 (Cqar), 123.6 (Cqar), 124.7 (Cqar), 126.2 (CHar), 128.2 (CHar), 128.8 (CHar), 130.9 (Cqar), 139.5 (Cqar), 147.1 (Cqar), 147.5 (Cqar), 148.0 (Cqar), 160.4 (Cqar), 161.3 (Cqar), 167.1 (Cq); Elemental analysis: calcd. for C_32_H_37_N_3_O_4_ C 72.84, H 7.07, N 7.96%, found C 72.76, H 7.31, N 7.76%.

#### 3.2.10. 2-Benzyl-3-morpholino-6-(3-morpholinopropyl)-7-phenyl-6,7-dihydro-5*H*-pyrrolo[3,4-*b*]pyridin-5-one **11j**

According to GP-1, 3-morpholinopropan-1-amine (14.6 µL), benzaldehyde (10.2 µL), scandium (III) triflate (1.5 mg), 2-isocyano-1-morpholino-3-phenylpropan-1-one (29.3 mg), and maleic anhydride (13.7 mg) were reacted together in PhH (0.2 mL) to afford the product **11j** (27.7 mg, 54%) as a yellow viscous liquid; R*_f_* = 0.20 (Hex–AcOEt = 1/1, *v*/*v*); FT–IR (ATR) υ_max_/cm^−1^ 698, 1031, 1115, 1252, 1443, 1512, 1689; ^1^H-NMR (500 MHz, CDCl_3_, 25 °C): δ 1.67–1.73 (m, 1H), 1.77–1.82 (m, 1H), 2.29–2.37 (m, 6H), 2.76–2.83 (m, 4H), 2.99–3.05 (m, 1H), 3.58–3.66 (m, 4H), 3.76–3.79 (m, 4H), 3.94–3.99 (m, 1H), 4.20 (d, *J* = 13.9 Hz, 1H), 4.28 (d, *J* = 13.9 Hz, 1H), 5.49 (s, 1H), 7.08–7.11 (m, 1H), 7.14–7.15 (m, 4H), 7.16–7.18 (m, 2H), 7.32–7.35 (m, 3H), 7.87 (s, 1H); ^13^C-NMR (126 MHz, CDCl_3_, 25 °C): δ 25.0 (CH_2_), 38.7 (CH_2_), 40.0 (CH_2_), 53.0 (CH_2_), 53.6 (CH_2_), 56.0 (CH_2_), 65.6 (CH), 66.8 (CH_2_), 67.1 (CH_2_), 123.7 (CHar), 124.2 (Cqar), 126.1 (CHar), 128.0 (CHar), 128.1 (CHar), 128.6 (CHar), 128.7 (CHar), 128.9 (CHar), 135.7 (Cqar), 139.3 (Cqar), 147.8 (Cqar), 160.4 (Cqar), 161.8 (Cqar), 167.2 (Cq); HRMS (EI): calcd. for C_31_H_36_N_4_O_3_ 512.2787, found 512.2795.

#### 3.2.11. 2-Benzyl-7-(4-fluorophenyl)-3-morpholino-6-(3-morpholinopropyl)-6,7-dihydro-5*H*-pyrrolo[3,4-*b*]pyridin-5-one **11k**

According to GP-1, 3-morpholinopropan-1-amine (14.6 µL), 4-fluorobenzaldehyde (10.7 µL), scandium (III) triflate (1.5 mg), 2-isocyano-1-morpholino-3-phenylpropan-1-one (29.3 mg), and maleic anhydride (13.7 mg) were reacted together in PhH (0.2 mL) to afford the product **11k** (26.0 mg, 49%) as a yellow viscous liquid; R*_f_* = 0.32 (Hex–AcOEt = 1/1, *v*/*v*); FT–IR (ATR) υ_max_/cm^−1^ 699, 862, 1032, 1115, 1221, 1443, 1508, 1692; ^1^H-NMR (500 MHz, CDCl_3_, 25 °C): δ 1.69–1.74 (m, 1H), 1.77–1.83 (m, 1H), 2.32–2.38 (m, 6H), 2.79–2.85 (m, 4H), 2.96–3.02 (m, 1H), 3.61–3.64 (m, 4H), 3.79–3.82 (m, 4H), 4.00–3.95 (m, 1H), 4.22 (d, *J* = 13.9 Hz, 1H), 4.29 (d, *J* = 13.9 Hz, 1H), 5.48 (s, 1H), 7.03–7.08 (m, 2H), 7.13–7.18 (m, 7H), 7.87 (s, 1H); ^13^C-NMR (126 MHz, CDCl_3_, 25 °C): δ 25.2 (CH_2_), 38.7 (CH_2_), 40.1 (CH_2_), 53.1 (CH_2_), 53.7 (CH_2_), 56.1 (CH_2_), 64.9 (CH), 67.0 (CH_2_), 67.2 (CH_2_), 116.1 (d, *^o^J*_CF_ = 21.8 Hz, CF), 123.8 (CHar), 124.2 (Cqar), 126.3 (CHar), 128.3 (CHar), 128.8 (CHar), 129.7 (d, *^m^J*_CF_ = 8.3 Hz, CF), 131.5 (d, *^p^J*_CF_ = 3.2 Hz, CF), 139.3 (Cqar), 148.0 (Cqar), 160.3 (Cqar), 162.0 (Cqar), 162.8 (d, *^i^J*_CF_ = 247.6 Hz, CF), 167.2 (CO); HRMS (EI): calcd. for C_31_H_35_FN_4_O_3_ 530.2693, found 530.2694.

#### 3.2.12. 2-Benzyl-7-(4-methoxyphenyl)-3-morpholino-6-(3-morpholinopropyl)-6,7-dihydro-5*H*-pyrrolo[3,4-*b*]pyridin-5-one **11l**

According to GP-1, 3-morpholinopropan-1-amine (14.6 µL), 4-methoxybenzaldehyde (12.2 µL), scandium (III) triflate (1.5 mg), 2-isocyano-1-morpholino-3-phenylpropan-1-one (29.3 mg), and maleic anhydride (13.7 mg) were reacted together in PhH (0.2 mL) to afford the product **11l** (25.5 mg, 47%) as a yellow viscous liquid; R*_f_* = 0.17 (AcOEt–EtOH = 10/1, *v*/*v*); FT–IR (ATR) υ_max_/cm^−1^ 1115, 1252, 1443, 1512, 1689, 2852; ^1^H-NMR (500 MHz, CDCl_3_, 25 °C): δ 1.67–1.73 (m, 1H), 1.77–1.83 (m, 1H), 2.30–2.39 (m, 6H), 2.85–2.76 (m, 4H), 2.98–3.03 (m, 1H), 3.62–3.65 (m, 4H), 3.78–380 (m, 7H), 3.91–3.96 (m, 1H), 4.21 (d, *J* = 13.9 Hz, 1H), 4.30 (d, *J* = 13.9 Hz, 1H), 5.45 (s, 1H), 6.88–6.89 (m, 2H), 7.07–7.09 (m, 2H), 7.13–7.18 (m, 5H), 7.87 (s, 1H); ^13^C-NMR (126 MHz, CDCl_3_, 25 °C): δ 25.1 (CH_2_), 38.5 (CH_2_), 40.1 (CH_2_), 53.1 (CH_2_), 53.6 (CH_2_), 55.3 (CH_3_), 56.1 (CH_2_), 65.2 (CH), 66.9 (CH_2_), 67.1 (CH_2_), 114.4 (CHar), 123.7 (CHar), 124.3 (Cqar), 126.1 (CHar), 127.4 (Cqar), 128.2 (CHar), 128.7 (CHar), 129.3 (CHar), 139.4 (Cqar), 147.8 (Cqar), 159.9 (Cqar), 160.7 (Cqar), 161.8 (Cqar), 167.0 (CO); HRMS (FAB): calcd. for C_32_H_38_N_4_O_4_ 542.2893, found 542.2890.

#### 3.2.13. 2-Benzyl-7-hexyl-3-morpholino-6-(3-morpholinopropyl)-6,7-dihydro-5*H*-pyrrolo[3,4-*b*]pyridin-5-one **11m**

According to GP-1, 3-morpholinopropan-1-amine (14.6 µL), heptanal (14.0 µL), scandium (III) triflate (1.5 mg), 2-isocyano-1-morpholino-3-phenylpropan-1-one (29.3 mg), and maleic anhydride (13.7 mg) were reacted together in PhH (0.2 mL) to afford the product **11m** (25.5 mg, 49%) as a yellow viscous liquid; R*_f_* = 0.40 (Hex–AcOEt = 1/1, *v*/*v*); FT–IR (ATR) υ_max_/cm^−1^ 700, 1031, 1115, 1399, 1446, 1648; ^1^H-NMR (500 MHz, CDCl_3_, 25 °C): δ 0.71–0.79 (m, 1H), 0.84 (d, *J* = 7.1 Hz, 3H), 1.10–1.25 (m, 8H), 1.79–1.91 (m, 3H), 2.23–2.17 (m, 1H), 2.39–2.43 (m, 6H), 2.80–2.87 (m, 4H), 3.23–3.27 (m, 1H), 3.65–3.70 (m, 4H), 3.82 (t, *J* = 4.6 Hz, 4H), 4.05 (m, 1H), 4.28 (d, *J* = 14.0 Hz, 1H), 4.43 (d, *J* = 14.0 Hz, 1H), 4.56 (dd, *J* = 3.2, 5.7 Hz, 1H), 7.15–7.19 (m, H), 7.22–7.28 (m, 4H), 7.82 (s, 1H); ^13^C-NMR (126 MHz, CDCl_3_, 25 °C): δ 14.1 (CH_3_), 22.5 (2 CH_2_), 25.4 (CH_2_), 29.2 (CH_2_), 29.4 (CH_2_), 31.6 (CH_2_), 38.1 (CH_2_), 40.0 (CH_2_), 53.1 (CH_2_), 53.7 (CH_2_), 56.2 (CH_2_), 60.5 (CH), 67.0 (CH_2_), 67.2 (CH_2_), 123.4 (CHar), 125.0 (Cqar), 126.2 (CHar), 128.3 (CHar), 128.8 (CHar), 139.6 (Cqar), 147.5 (Cqar), 160.3 (Cqar), 161.1 (Cqar), 167.1 (CO); HRMS (EI): calcd. for C_31_H_44_N_4_O_3_ 520.3413, found 520.3414.

#### 3.2.14. 2-Benzyl-7-(4-chlorophenyl)-6-(3-morpholinopropyl)-3-(piperidin-1-yl)-6,7-dihydro-5*H*-pyrrolo[3,4-*b*]pyridin-5-one **11n**

According to GP-1, 3-morpholinopropan-1-amine (14.6 µL), 4-chlorobenzaldehyde (14.1 mg), scandium (III) triflate (1.5 mg), 2-benzyl-3-oxo-3-(piperidin-1-yl)propanenitrile (29.1 mg), and maleic anhydride (13.7 mg) were reacted together in PhH (0.2 mL) to afford the product **11n** (24.5 mg, 45%) as a yellow viscous liquid; R*_f_* = 0.33 (AcOEt–EtOH = 10/1, *v*/*v*); FT–IR (ATR) υ_max_/cm^−1^ 680, 755, 781, 833, 982, 1036, 1168, 1250, 1385, 1515, 1759; ^1^H-NMR (500 MHz, CDCl_3_, 25 °C): δ 1.54–1.60 (m, 2H), 1.68–1.73 (m, 5H), 1.76–1.82 (m, 1H), 3.31–3.40 (m, 6H), 2.75–2.81 (m, 4H), 2.94–3.00 (m, 1H), 3.62–3.65 (m, 4H), 3.94–4.00 (m, 1H), 4.16 (d, *J* = 13.8 Hz, 1H), 4.25 (d, *J* = 13.9 Hz, 1H), 5.43 (s, 1H), 7.09–7.20 (m, 7H), 7.32–7.33 (m, 2H), 7.80 (s, 1H); ^13^C-NMR (126 MHz, CDCl_3_, 25 °C): δ 24.1 (CH_2_), 25.2 (CH_2_), 26.5 (CH_2_), 38.8 (CH_2_), 39.9 (CH_2_), 53.7 (CH_2_), 54.4 (CH_2_), 56.2 (CH_2_), 64.9 (CH), 67.0 (CH_2_), 123.2 (CHar), 123.9 (Cqar), 126.1 (CHar), 128.2 (CHar), 129.0 (CHar), 129.2 (CHar), 129.4 (CHar), 134.6 (2 Cqar), 139.6 (Cqar), 149.7 (Cqar), 159.2 (Cqar), 162.2 (Cqar), 167.6 (CO); HRMS (EI): calcd. for C_32_H_38_ClN_4_O_2_^+^ 545.2683, found 545.2639.

#### 3.2.15. 2-Benzyl-7-(4-methoxyphenyl)-6-(3-morpholinopropyl)-3-(piperidin-1-yl)-6,7-dihydro-5*H*-pyrrolo[3,4-*b*]pyridin-5-one **11o**

According to GP-1, 3-morpholinopropan-1-amine (14.6 µL), 4-methoxybenzaldehyde (12.2 µL), scandium (III) triflate (1.5 mg), 2-benzyl-3-oxo-3-(piperidin-1-yl)propanenitrile (29.1 mg), and maleic anhydride (13.7 mg) were reacted together in PhH (0.2 mL) to afford the product **11o** (25.4 mg, 47%) as a yellow viscous liquid; R*_f_* = 0.28 (AcOEt–EtOH = 10/1, *v*/*v*); FT–IR (ATR) υ_max_/cm^−1^ 1115, 1252, 1443, 1512, 1689, 2852; ^1^H-NMR (500 MHz, CD_3_OD, 25 °C): δ 1.67–1.74 (m, 1H), 1.77–1.84 (m, 1H), 2.35–2.49 (m, 10H), 2.81–2.88 (m, 4H), 3.06–3.11 (m, 1H), 3.61–3.65 (m, 5H), 3.76–3.78 (m, 2H), 3.79 (s, 3H), 4.24 (d, *J* = 14.2 Hz, 1H), 4.32 (d, *J* = 14.1 Hz, 1H), 5.62 (s, 1H), 6.93–6.95 (m, 2H), 7.08–7.16 (m, 7H), 7.98 (s, 1H); ^13^C-NMR (126 MHz, CD_3_OD, 25 °C): δ 25.5 (CH_2_), 39.8 (CH_2_), 40.7 (CH_2_), 54.1 (CH_2_), 54.4 (CH_2_), 55.8 (CH_3_), 57.0 (CH_2_), 66.7 (CH), 67.4 (CH_2_), 68.1 (CH_2_), 115.5 (CHar), 125.2 (CHar), 125.6 (Cqar), 127.2 (CHar), 128.3 (Cqar), 129.2 (CHar), 129.7 (CHar), 130.7 (CHar), 140.6 (Cqar), 149.7 (Cqar), 161.7 (Cqar), 162.1 (Cqar), 163.6 (Cqar), 168.9 (CO); HRMS (EI): calcd. for C_33_H_40_N_4_O_3_ 540.3100, found 540.2995.

#### 3.2.16. 6-(2-(1*H*-Indol-3-yl)ethyl)-2-benzyl-7-(2-bromophenyl)-3-morpholino-6,7-dihydro-5*H*-pyrrolo[3,4-*b*]pyridin-5-one **11p**

According to GP-1, tryptamine (16.0 mg), 2-bromobenzaldehyde (11.7 µL), scandium (III) triflate (1.5 mg), 2-isocyano-1-morpholino-3-phenylpropan-1-one (29.3 mg), and maleic anhydride (13.7 mg) were reacted together in PhH (0.2 mL) to afford the product **11p** (39.4 mg, 65%) as a yellow solid; mp = 99–101 °C; R*_f_* = 0.33 (Hex–AcOEt = 1/1, *v*/*v*); FT–IR (ATR) υ_max_/cm^−1^ 420, 695, 738, 1112, 1440, 1674, 3287; ^1^H-NMR (500 MHz, CDCl_3_, 25 °C): δ 2.81–2.83 (m, 4H), 3.01–3.07 (m, 1H), 3.17–3.21 (m, 2H), 3.78–3.81 (m, 1H), 3.83–3.84 (m, 4H), 4.21 (d, *J* = 13.8 Hz, 1H), 4.32 (d, *J* = 13.9 Hz, 1H), 6.23 (s, 1H), 6.71–6.73 (m, 1H), 7.01 (d, *J* = 2.1 Hz, 1H), 7.06–7.09 (m, 1H), 7.15–7.23 (m, 8H), 7.28–7.29 (m, 1H), 7.55 (d, *J* = 7.9 Hz, 1H), 7.69–7.71 (m, 1H), 7.89 (s, 1H), 8.64 (s, 1H); ^13^C-NMR (126 MHz, CDCl_3_, 25 °C): δ 29.4 (CH_2_), 40.0 (CH_2_), 41.2 (CH_2_), 53.0 (CH_2_), 63.9 (CH), 67.1 (CH_2_), 111.3 (CHar), 112.2 (Cqar), 118.6 (CHar), 119.2 (CHar), 121.9 (CHar), 122.1 (CHar), 123.6 (CHar), 124.3 (Cqar), 125.7 (Cqar), 126.2 (CHar), 127.4 (Cqar), 128.0 (CHar), 128.2 (CHar), 128.9 (CHar), 130.1 (CHar), 133.4 (CHar), 135.1 (Cqar), 136.4 (Cqar), 139.2 (Cqar), 147.9 (Cqar), 160.3 (Cqar), 162.0 (Cqar), 167.4 (CO); HRMS (EI): calcd. for C_34_H_32_BrN_4_O_2_ 607.1708, found 607.1710.

#### 3.2.17. 2-Benzyl-7-hexyl-6-(2-hydroxyethyl)-3-morpholino-6,7-dihydro-5*H*-pyrrolo[3,4-*b*]pyridin-5-one **11q**

According to GP-1, 2-aminoethan-1-ol (6.0 µL), heptanal (14.0 µL), scandium (III) triflate (1.5 mg), 2-isocyano-1-morpholino-3-phenylpropan-1-one (29.3 mg), and maleic anhydride (13.7 mg) were reacted together in PhH (0.2 mL) to afford the product **11q** (8.7 mg, 20%) as a yellow viscous liquid; R*_f_* = 0.22 (AcOEt); FT–IR (ATR) υ_max_/cm^−1^ 1115, 1399, 1447, 1672; ^1^H-NMR (500 MHz, CDCl_3_, 25 °C): δ 0.73–0.78 (m, 1H), 0.84 (t, *J* = 7.1 Hz, 3H), 1.15–1.21 (m, 7H), 1.83–1.90 (m, 2H), 2.19–2.24 (m, 1H), 2.82–2.85 (m, 4H), 3.44–3.47 (m, 1H), 3.81–3.83 (m, 4H), 3.86–3.89 (m, 2H), 3.96–3.99 (m, 1H), 4.28 (d, *J* = 14.0 Hz, 1H), 4.42 (d, *J* = 14.0 Hz, 1H), 4.64–4.66 (m, 1H), 7.16–7.18 (m, 1H), 7.23–7.25 (m, 4H), 7.82 (s, 1H); ^13^C-NMR (126 MHz, CDCl_3_, 25 °C): δ 14.4 (CH_3_), 22.6 (2 CH_2_), 29.3 (CH_2_), 29.6 (CH_2_), 31.7 (CH_2_), 40.1 (CH_2_), 44.3 (CH_2_), 53.2 (CH_2_), 62.0 (CH_2_), 62.3 (CH), 67.3 (CH_2_), 123.6 (CHar), 124.7 (Cqar), 126.3 (CHar), 128.4 (CHar), 128.9 (CHar), 139.6 (Cqar), 147.7 (Cqar), 160.5 (Cqar), 161.6 (Cqar), 168.7 (CO); HRMS (EI): calcd. for C_26_H_36_N_3_O_3_^+^ 438.2757, found 438.2777.

#### 3.2.18. 2-Benzyl-6-(2-hydroxyethyl)-7-(3-methoxyphenyl)-3-morpholino-6,7-dihydro-5*H*-pyrrolo[3,4-*b*]pyridin-5-one **11r**

According to GP-1, 2-aminoethan-1-ol (6.0 µL), 3-methoxybenzaldehyde (12.2 µL), scandium (III) triflate (1.5 mg), 2-isocyano-1-morpholino-3-phenylpropan-1-one (29.3 mg), and maleic anhydride (13.7 mg) were reacted together in PhH (0.2 mL) to afford the product **11r** (19.4 mg, 43%) as a yellow viscous liquid; R*_f_* = 0.11 (Hex–AcOEt = 1/1, *v*/*v*); FT–IR (ATR) υ_max_/cm^−1^ 1114, 1261, 1393, 1444, 1680, 2919; ^1^H-NMR (500 MHz, CDCl_3_, 25 °C): δ 2.83 (s, 1H), 2.77–2.82 (m, 4H), 3.16–3.19 (m, 1H), 3.40–3.43 (m, 1H), 3.56–3.59 (m, 1H), 3.74 (s, 3H), 3.77–3.79 (m, 4H), 3.95–3.99 (m, 1H), 4.2 (d, *J* = 14.0 Hz, 1H), 4.29 (d, *J* = 14.0 Hz, 1H), 5.61 (s, 1H), 6.70–6.71 (m, 1H), 6.77–6.78 (m, 1H), 6.86 (ddd, *J* = 0.9, 2.6, 8.3 Hz, 1H), 7.13–7.16 (m, 5H), 7.18–7.20 (m, 1H), 7.87 (s, 1H); ^13^C-NMR (126 MHz, CDCl_3_, 25 °C): δ 40.0 (CH_2_), 43.8 (CH_2_), 53.0 (CH_2_), 55.3 (CH_3_), 61.2 (CH_2_), 66.6 (CH), 67.1 (CH_2_), 113.7 (CHar), 114.0 (CHar), 120.3 (CHar), 126.2 (CHar), 127.4 (Cqar), 128.2 (CHar), 128.8 (CHar), 129.5 (CHar), 130.0 (CHar), 136.9 (Cqar), 139.2 (Cqar), 147.9 (Cqar), 160.0 (Cqar), 160.4 (Cqar), 162.1 (Cqar), 168.2 (CO); HRMS (EI): calcd. for C_27_H_29_N_3_O_4_ 459.2158, found 459.2158.

#### 3.2.19. 2-Benzyl-6-(2-bromoethyl)-3-morpholino-7-phenyl-6,7-dihydro-5*H*-pyrrolo[3,4-*b*]pyridin-5-one **11s**

According to GP-1, 22-bromoethan-1-amina (20.5 mg), benzaldehyde (10.2 µL), scandium (III) triflate (1.5 mg), 2-isocyano-1-morpholino-3-phenylpropan-1-one (29.3 mg), and maleic anhydride (13.7 mg) were reacted together in dry PhH (0.2 mL) to afford the product **11s** (15.2 mg, 31%) as a yellow viscous liquid; R*_f_* = 0.48 (Hex–AcOEt = 1/1, *v*/*v*); FT–IR (ATR) υ_max_/cm^−1^ 699, 747, 1114, 1391, 1443, 1696; ^1^H-NMR (500 MHz, CDCl_3_, 25 °C): δ 2.78–2.82 (m, 4H), 3.32–3.40 (m, 2H), 3.57–3.61 (m, 1H), 3.78–3.80 (m, 4H), 4.21 (d, *J* = 14.0 Hz, 1H), 4.29 (d, *J* = 13.9 Hz, 1H), 4.30–4.33 (m, 1H), 5.72 (s, 1H), 7.11–7.18 (m, 8H), 7.36–7.38 (m, 2H), 7.92 (s, 1H); ^13^C-NMR (126 MHz, CDCl_3_, 25 °C): δ 29.6 (CH_2_), 40.1 (CH_2_), 42.3 (CH_2_), 53.0 (CH_2_), 66.3 (CH), 67.1 (CH_2_), 123.6 (Cqar), 124.0 (CHar), 126.2 (CHar), 128.0 (CHar), 128.2 (CHar), 128.7 (CHar), 128.9 (CHar), 129.1 (CHar), 135.1 (Cqar), 139.1 (Cqar),147.9 (Cqar), 160.4 (Cqar), 162.4 (Cqar), 167.5 (CO); HRMS (EI): calcd. for C_26_H_26_BrN_3_O_2_ 491.1208, found 491.1208.

### 3.3. One-Pot Synthesis and Characterization of the Polysubstituted Pyrrolo[3,4-*b*]pyridin-5-ones **11t**–**x**

General procedure 2 (GP-2): The 2-bromoethan-1-amine (0.1 mmol, 1.0 equiv.) and the corresponding aldehydes (1.0 equiv.) were placed in a 10 mL sealed CEM Discover microwave reaction tube and diluted in benzene [0.5 M]. The mixture was stirred and irradiated (MW, 65 °C, 55 W) for 15 min, and then Sc(OTf)_3_ (0.03 equiv.) was added. The mixture was stirred and irradiated (MW, 65 °C, 55 W) for 15 min, and then the corresponding isocyanides (1.2 equiv.) were added. The mixture was stirred and irradiated (MW, 80 °C, 100 W) for 30 min, and then maleic anhydride (1.4 equiv.) was added. The mixture was stirred and irradiated (MW, 80 °C, 100 W) for 30 min, and then the solvent was removed to dryness under vacuum. Anhydrous acetonitrile [0.5 M] was added. Finally, the corresponding secondary amine (1.0 equiv.) and triethylamine (1.1 equiv.) were sequentially added, and then the new reaction mixture was stirred and irradiated (MW, 80 °C, 100 W) for 30 min. Then, the solvent was removed to dryness under vacuum. The crude was diluted in dichloromethane (5.0 mL), washed with a concentrated aqueous solution of NaHCO_3_ (3 × 25 mL), and then washed with brine (3 × 25 mL). The organic layer was dried using anhydrous Na_2_SO_4_ and then filtered over a celite pad. The solvent was removed to dryness under vacuum. The residue was purified immediately using a silica-gel column chromatography followed by a preparative TLC using mixtures of Hex–EtOAc or EtOAc-EtOH (*v*/*v*) in different proportions as mobile phase to afford the corresponding polyheterocyclic pyrrolo[3,4-*b*]pyridin-5-ones **11t**–**x**.

#### 3.3.1. 2-Benzyl-3-morpholino-6-(2-morpholinoethyl)-7-phenyl-6,7-dihydro-5*H*-pyrrolo[3,4-*b*]pyridin-5-one **11t**

According to GP-2, 2-bromoethan-1-amine (20.5 mg), benzaldehyde (10.2 µL), scandium (III) triflate (1.5 mg), 2-isocyano-1-morpholino-3-phenylpropan-1-one (29.3 mg), maleic anhydride (13.7 mg), morpholine (8.7 µL), and triethylamine (15.3 µL) were reacted together first in PhH (0.2 mL) and then in MeCN (0.2 mL) to afford the product **11t** (17.4 mg, 35%) as a yellow solid; mp = 71–73 °C; R*_f_* = 0.17 (AcOEt); FT–IR (ATR) υ_max_/cm^−1^ 1015, 1115, 1393, 1443, 1693; ^1^H-NMR (500 MHz, CDCl_3_, 25 °C): δ 2.38–2.43 (m, 4H), 2.45–2.50 (m, 1H), 2.56–2.61 (m, 1H), 2.78–2.84 (m, 4H), 3.00–3.06 (m, 1H), 3.66–3.67 (m, 4H), 3.68–3.80 (m, 4H), 4.09–4.14 (m, 1H), 4.21 (d, *J* = 13.9 Hz, 1H), 4.28 (d, *J* = 13.9 Hz, 1H), 5.73 (s, 1H), 7.14–7.19 (m, 8H), 7.34–7.36 (m, 2H), 7.88 (s, 1H); ^13^C-NMR (126 MHz, CDCl_3_, 25 °C): δ 36.8 (CH_2_), 40.1 (CH_2_), 53.1 (CH_2_), 53.7 (CH_2_), 57.3 (CH_2_), 66.2 (CH), 67.0 (CH_2_), 67.2 (CH_2_), 123.8 (CHar), 126.2 (CHar), 127.8 (CHar), 128.2 (CHar), 128.6 (CHar), 128.8 (CHar), 129.0 (CHar), 129.6 (Cqar), 135.8 (Cqar), 139.3 (Cqar), 147.7 (Cqar), 160.8 (Cqar), 161.8 (Cqar), 167.2 (Cq); HRMS (EI): calcd. for C_30_H_34_N_4_O_3_ 498.2631, found 498.2631.

#### 3.3.2. 2-Benzyl-7-(4-methoxyphenyl)-6-(2-(4-(2-methoxyphenyl)piperazin-1-yl)ethyl)-3-morpholino-6,7-dihydro-5*H*-pyrrolo[3,4-*b*]pyridin-5-one **11u**

According to GP-2, 2-bromoethan-1-amine (20.5 mg), 4-methoxybenzaldehyde (12.2 µL), scandium (III) triflate (1.5 mg), 2-isocyano-1-morpholino-3-phenylpropan-1-one (29.3 mg), maleic anhydride (13.7 mg), 1-(2-methoxyphenyl)piperazine (19.2 mg), and triethylamine (15.3 µL) were reacted together first in PhH (0.2 mL) and then in MeCN (0.2 mL) to afford the product **11u** (21.5 mg, 34%) as a yellow viscous liquid; R*_f_* = 0.71 (AcOEt–EtOH = 10/1, *v*/*v*); FT–IR (ATR) υ_max_/cm^−1^ 1114, 1241, 1444, 1504, 1689, 2821; ^1^H-NMR (500 MHz, CDCl_3_, 25 °C): δ 2.59–2.71 (m, 4H), 2.78–2.82 (m, 6H), 3.05–3.08 (m, 4H), 3.78–3.79 (m, 5H), 3.80 (s, 3H), 3.84 (s, 3H), 4.07–4.13 (m, 1H), 4.22 (d, *J* = 13.9 Hz, 1H), 4.29 (d, *J* = 13.9 Hz, 1H), 5.73 (s, 1H), 6.86–6.91 (m, 6H), 7.11–7.16 (m, 7H), 7.89 (s, 1H); ^13^C-NMR (126 MHz, CDCl_3_, 25 °C): δ 36.9 (CH_2_), 40.1 (CH_2_), 50.5 (CH_2_), 53.0 (CH_2_), 53.4 (CH_2_), 55.3 (CH_3_), 55.4 (CH_3_), 56.9 (CH_2_), 65.8 (CH), 67.1 (CH_2_), 111.2 (CHar), 114.4 (CHar), 118.2 (CHar), 121.0 (CHar), 123.0 (CHar), 123.9 (CHar), 124.0 (Cqar), 126.1 (CHar), 127.5 (Cqar), 128.2 (CHar), 128.7 (CHar), 129.1 (CHar), 139.3 (Cqar), 141.2 (Cqar), 147.7 (Cqar), 152.2 (Cqar), 159.8 (Cqar), 161.1 (Cqar), 161.8 (Cqar), 167.0 (Cq); HRMS (EI): calcd. for C_38_H_43_N_5_O_4_ 633.3315, found 633.3317.

#### 3.3.3. 2-Benzyl-7-(4-fluorophenyl)-6-(2-(4-(2-methoxyphenyl)piperazin-1-yl)ethyl)-3-morpholino-6,7-dihydro-5*H*-pyrrolo[3,4-*b*]pyridin-5-one **11v**

According to GP-2, 2-bromoethan-1-amine (20.5 mg), 4-fluorobenzaldehyde (10.7 µL), scandium (III) triflate (1.5 mg), 2-isocyano-1-morpholino-3-phenylpropan-1-one (29.3 mg), maleic anhydride (13.7 mg), 1-(2-methoxyphenyl)piperazine (19.2 mg), and triethylamine (15.3 µL) were reacted together first in PhH (0.2 mL) and then in MeCN (0.2 mL) to afford the product **11v** (20.5 mg, 33%) as a yellow viscous liquid; R*_f_* = 0.73 (AcOEt–EtOH = 10/1, *v*/*v*); FT–IR (ATR) υ_max_/cm^−1^ 698, 748, 1027, 1114, 1239, 1444, 1500, 1693, 2819; ^1^H-NMR (500 MHz, CDCl_3_, 25 °C): δ 2.52–2.73 (m, 6H), 2.80–2.83 (m, 4H), 3.04–3.08 (m, 4H), 3.69–3.75 (m, 1H), 3.78–3.80 (m, 4H), 3.84 (s, 3H), 4.11–4.16 (m, 1H), 4.23 (q, *J* =13.9 Hz, 1H), 4.27 (q, *J* =14.0 Hz, 1H), 5.79 (s, 1H), 6.84–6.86 (m, 1H), 6.91–6.93 (m, 2H), 6.97–7.00 (m, 1H), 7.03–7.07 (m, 3H), 7.11–7.19 (m, 6H), 7.89 (s, 1H); ^13^C-NMR (126 MHz, CDCl_3_, 25 °C): δ 37.0 (CH_2_), 40.1 (CH_2_), 50.6 (CH_2_), 53.1 (CH_2_), 53.4 (CH_2_), 55.4 (CH_3_), 57.0 (CH_2_), 65.6 (CH), 67.1 (CH_2_), 111.3 (CHar), 116.0 (d, *^o^J*_CF_ = 21.7 Hz) (CHar), 118.2 (CHar), 121.0 (CHar), 123.0 (CHar), 123.9 (CHar), 126.2 (CHar), 128.2 (CHar), 128.8 (CHar), 129.5 (d, *^m^J*_CF_ = 8.3 Hz) (CHar), 131.6 (d, *^p^J*_CF_ = 3.1 Hz) (Cqar), 139.2 (Cqar), 141.2 (Cqar), 147.8 (Cqar), 152.3 (Cqar), 160.6 (Cqar), 161.9 (Cqar), 162.8 (d, *^i^J*_CF_ = 247.4 Hz) (Cqar), 167.1 (Cq); HRMS (EI): calcd. for C_37_H_40_FN_5_O_3_ 621.3115, found 621.3113.

#### 3.3.4. 2-Benzyl-6-(2-((3,4-dimethoxybenzyl)(methyl)amino)ethyl)-3-morpholino-7-phenyl-6,7-dihydro-5*H*-pyrrolo[3,4-*b*]pyridin-5-one **11w**

According to GP-2, 2-bromoethan-1-amine (20.5 mg), benzaldehyde (10.2 µL), scandium (III) triflate (1.5 mg), 2-isocyano-1-morpholino-3-phenylpropan-1-one (29.3 mg), maleic anhydride (13.7 mg), 2-(3,4-dimethoxyphenyl)-*N*-methylethan-1-amine (23.8 mg), and triethylamine (15.3 µL) were reacted together first in PhH (0.2 mL) and then in MeCN (0.2 mL) to afford the product **11w** (18.2 mg, 30%) as a yellow viscous liquid; R*_f_* = 0.35 (AcOEt–EtOH = 10/1, *v*/*v*); FT–IR (ATR) υ_max_/cm^−1^ 700, 1028, 1114, 1236, 1261, 1443, 1515, 1691; ^1^H-NMR (500 MHz, CDCl_3_, 25 °C): δ 2.31 (s, 3H), 2.54–2.64 (m, 2H), 2.66–2.74 (m, 2H), 2.80–2.83 (m, 4H), 2.99–3.07 (m, 2H), 3.79–3.81 (m, 4H), 3.82 (s, 3H), 3.84 (s, 3H), 3.85–3.87 (m, 1H), 4.01–4.13 (m, 1H), 4.23 (d, *J* = 13.9 Hz, 1H), 4.28 (d, *J* = 14.0 Hz, 1H), 5.66 (s, 1H), 6.67–6.70 (m, 2H), 6.73–6.75 (m, 1H), 7.17–7.18 (m, 7H), 7.36–7.35 (m, 3H), 7.89 (s, 1H); ^13^C-NMR (126 MHz, CDCl_3_, 25 °C): δ 33.2 (CH_2_), 37.8 (CH_2_), 40.1 (CH_2_), 42.1 (CH_2_), 53.1 (CH_2_), 55.5 (CH_2_), 55.9 (CH_3_), 56.0 (CH_3_), 59.7 (CH_2_), 66.2 (CH), 67.2 (CH_2_), 111.4 (CHar), 112.1 (CHar), 120.6 (CHar), 123.8 (CHar), 124.0 (Cqar), 126.2 (CHar), 128.0 (CHar), 128.2 (CHar), 128.7 (Cqar), 128.8 (CHar), 129.0 (CHar), 132.6 (Cqar), 135.8 (Cqar), 139.4 (Cqar), 147.4 (Cqar), 147.8 (Cqar), 148.9 (Cqar), 160.8 (Cqar), 161.8 (Cqar), 167.3 (Cq); HRMS (EI): calcd. for C_37_H_42_N_4_O_4_ [M] 606.3206, found 606.3208.

#### 3.3.5. 2-Benzyl-6-(2-((3,4-dimethoxybenzyl)(methyl)amino)ethyl)-7-(4-methoxyphenyl)-3-morpholino-6,7-dihydro-5*H*-pyrrolo[3,4-*b*]pyridin-5-one **11x**

According to GP-2, 2-bromoethan-1-amine (20.5 mg), 4-methoxybenzaldehyde (12.2 µL), scandium (III) triflate (1.5 mg), 2-isocyano-1-morpholino-3-phenylpropan-1-one (29.3 mg), maleic anhydride (13.7 mg), 2-(3,4-dimethoxyphenyl)-*N*-methylethan-1-amine (23.8 mg), and triethylamine (15.3 µL) were reacted together first in PhH (0.2 mL) and then in MeCN (0.2 mL) to afford the product **11x** (22.3 mg, 35%) as a yellow viscous liquid; R*_f_* = 0.33 (Hex–AcOEt = 1/1, *v*/*v*); FT–IR (ATR) υ_max_/cm^−1^ 698, 749, 1027, 1114, 1241, 1444, 1504, 1689, 2849; ^1^H-NMR (500 MHz, CDCl_3_, 25 °C): δ 2.29 (s, 3H), 2.52–2.54 (m, 1H), 2.59–2.61 (m, 1H), 2.66–2.67 (m, 2H), 2.79–2.82 (m, 4H), 2.97–3.02 (m, 1H), 3.77–3.80 (m, 9H), 3.81 (s, 3H), 3.82 (s, 3H), 4.05–4.08 (m, 1H), 4.23 (d, *J* = 13.9, Hz, 1H), 4.29 (d, *J* = 13.9 Hz, 1H), 5.60 (s, 1H), 6.67–6.69 (m, 2H), 6.72–6.74 (m, 1H), 6.87–6.89 (m, 2H), 7.06–7.08 (m, 2H), 7.10–7.13 (m, 1H), 7.13–7.16 (m, 6H), 7.88 (s, 1H); ^13^C-NMR (126 MHz, CDCl_3_, 25 °C): δ 33.3 (CH_2_), 37.7 (CH_2_), 40.0 (CH_2_), 42.1 (CH_2_), 53.1 (CH_2_), 55.3 (CH_3_), 55.6 (CH_2_), 55.8 (CH_3_), 55.9 (CH_3_), 59.8 (CH_2_), 65.6 (CH), 67.2 (CH_2_), 111.3 (CHar),112.0 (CHar), 120.5 (CHar), 123.7 (CHar), 124.1 (Cqar), 126.1 (CHar), 127.6 (CHar), 128.1 (CHar), 128.7 (CHar), 129.2 (CHar), 129.5 (Cqar), 132.8 (Cqar), 139.4 (Cqar), 147.3 (Cqar), 147.6 (Cqar), 148.8 (Cqar), 159.8 (Cqar), 161.0 (Cqar), 161.7 (Cqar), 167.0 (Cq); HRMS (EI): calcd. for C_38_H_44_N_4_O_5_ [M] 636.3312, found 636.3315.

## 4. Conclusions

The herein described one-pot synthetic strategy, based on multicomponent reactions, allowed for the rapid construction of various new polyheterocyclic compounds (for example, pyrrolo[3,4-*b*]pyridine-5-ones linked with other heterocycles like morpholines, piperidines, and piperazines). Hence, highly polysubstituted and polyheterocyclic pyrrolo[3,4-*b*]pyridin-5-ones, including some Falipamil aza-analogues and a pair of piperazine-linked analogues, were synthesized through a robust one-pot process. Likewise, a novel and complex sequence Ugi-3CR/aza Diels-Alder/*N*-acylation/aromatization (decarboxylation-dehydration) was carried out in a one-pot manner including an additional final S_N_2 step to provide a wide diversity of polyheterocycles. Despite the molecular complexity of the final products and the high number of formed bonds, modest to good yields were obtained. The high atomic economy (-2H_2_O and -CO_2_) provides to this methodology the category of ‘sustainable’. The final products could be considered for further SAR studies, since the pyrrolo[3,4-*b*]pyridine-5-one scaffold, piperazine-linker, and the drug falipamil are of high interest in medicinal chemistry.

## Supplementary Materials

The following are available online: ^1^H and ^13^C-NMR spectra of all the products **11a**–**x** Figures S1–S48.
