# Supplementary material for: Synthesis of Polyheterocyclic Pyrrolo[3,4-b]pyridin-5-ones via a One-Pot (Ugi-3CR/aza Diels-Alder/N-acylation/aromatization/SN2) Process. A Suitable Alternative towards Novel Aza-Analogues of Falipamil
Source: Molecules. 2018 Mar 27;23(4):763. doi: 10.3390/molecules23040763 (PMC6017480; doi:10.3390/molecules23040763)
Supplement: Supplementary file 1 [file molecules-23-00763-s001.pdf]

E L E C T R O N I C   S U P P L E M E N T A R Y   M A T E R I A L

Synthesis of polyheterocyclic pyrrolo[3,4-*b*]pyridin-5-ones via a one-pot (Ugi-3CR/aza Diels-Alder/*N*-acylation/aromatization/S_N2) process. A suitable alternative towards novel aza-analogues of falipamil

Ángel Zamudio-Medina[1], Ailyn N. García-González[2], Genesis K. Herrera-Carrillo[2], Daniel Zárate-Zárate[2], Adriana Benavides-Macías[3], Joaquín Tamariz[3], Ilich A. Ibarra[4,*], Alejandro Islas-Jácome[2,*], and Eduardo González-Zamora[2,*]

[1] Unidad Profesional Interdisciplinaria de Biotecnología, Instituto Politécnico Nacional, Av. Acueducto de Guadalupe S/N, Barr. La Laguna Ticomán, C.P. 07340, Del. Gustavo A Madero, Ciudad de México; angelzamu2015@hotmail.com

[2] Departamento de Química, Universidad Autónoma Metropolitana-Iztapalapa, San Rafael Atlixco 186, Col. Vicentina, C.P. 09340, Del. Iztapalapa, Ciudad de México; natgg@outlook.com (A.N.G.-G.); purpulove-@live.com.mx (G.K.H.-C.); danzarate_286@hotmail.com (D.Z.-Z.)

[3] Escuela Nacional de Ciencias Biológicas, Instituto Politécnico Nacional, Prol. Manuel Carpio y Plan de Ayala S/N, Col. Santo Tomás, C.P. 11350, Del. Miguel Hidalgo, Ciudad de México; a_benavides@prodigy.net.mx (A.B.-M.); jtamarizm@gmail.com (J.T.)

[4] Laboratorio de Fisicoquímica y Reactividad de Superficies, Instituto de Investigaciones en Materiales, Universidad Nacional Autónoma de México, Circuito Exterior S/N, Ciudad Universitaria, C.P. 04510, Del. Coyoacán, Ciudad de México.

* Correspondence: argel@unam.mx (I.A.I.); aij@xanum.uam.mx (A.I.-J.); egz@xanum.uam.mx (E.G.-Z.) Tel.: +52-55-6709-1138 (I.A.I.); +52-55-5804-4913 (A.I.-J., E.G.-Z.)

C O N T E N T S

^1H and ^{13}C NMR spectra of the products **11a-x** **S2-S49**

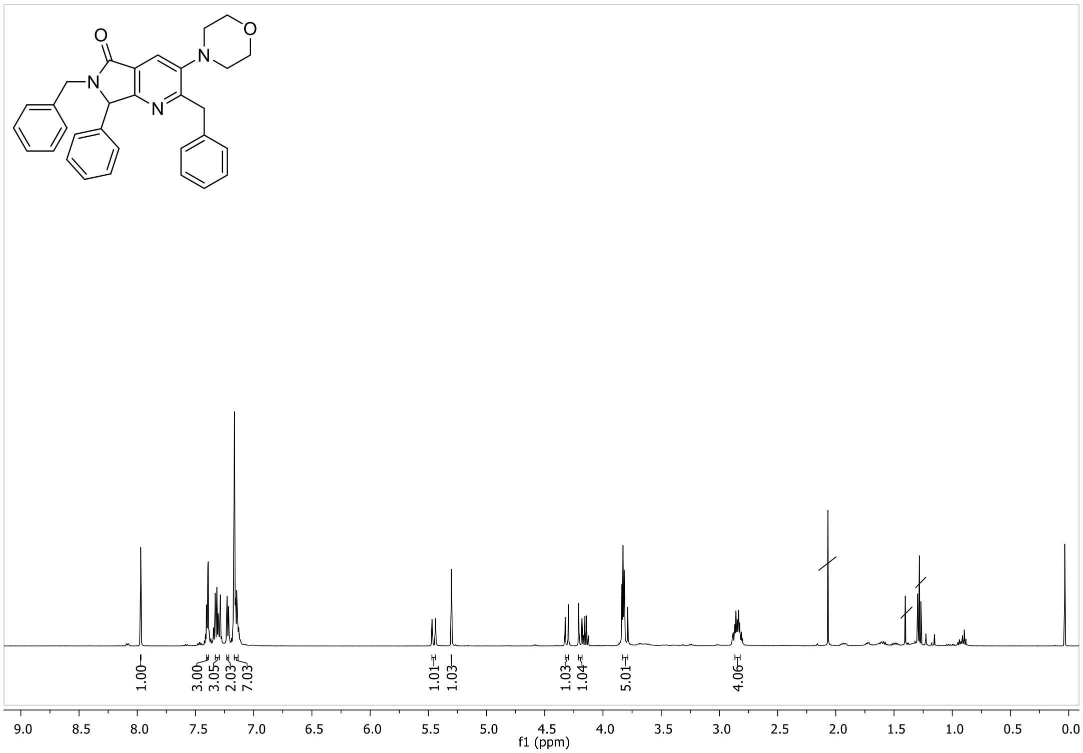

Figure S1. ^{1}H NMR spectrum of the product **11a**

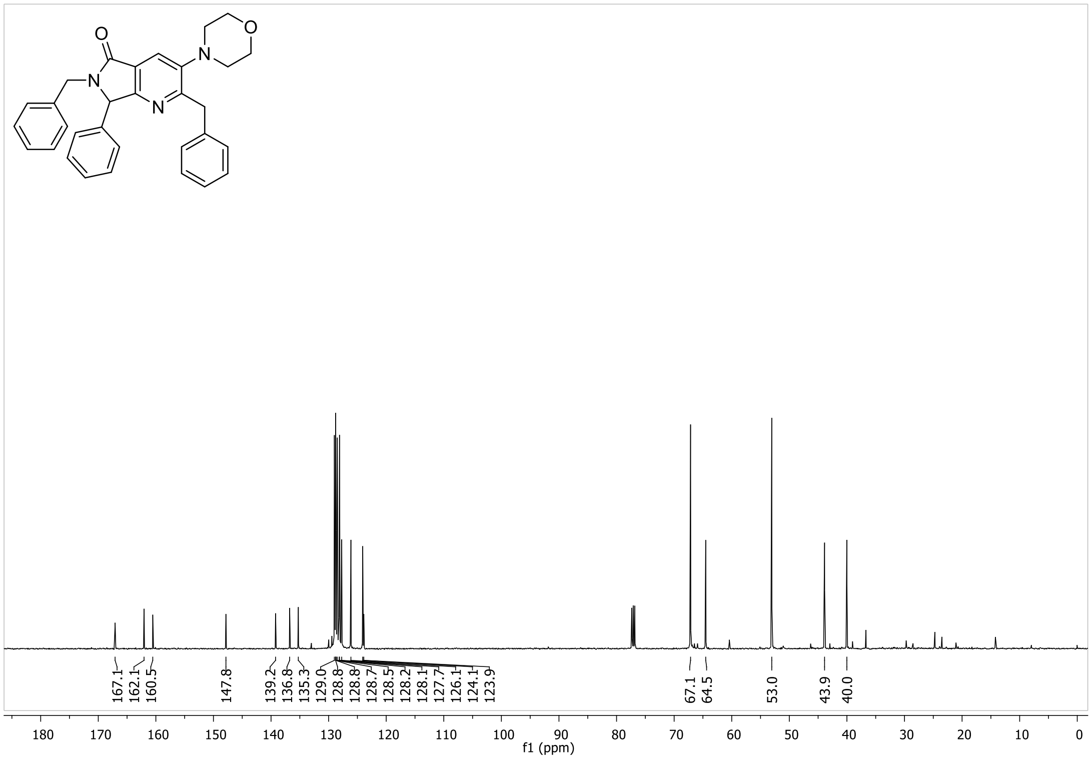

Figure S2. ^{13}C NMR spectrum of the product **11a**

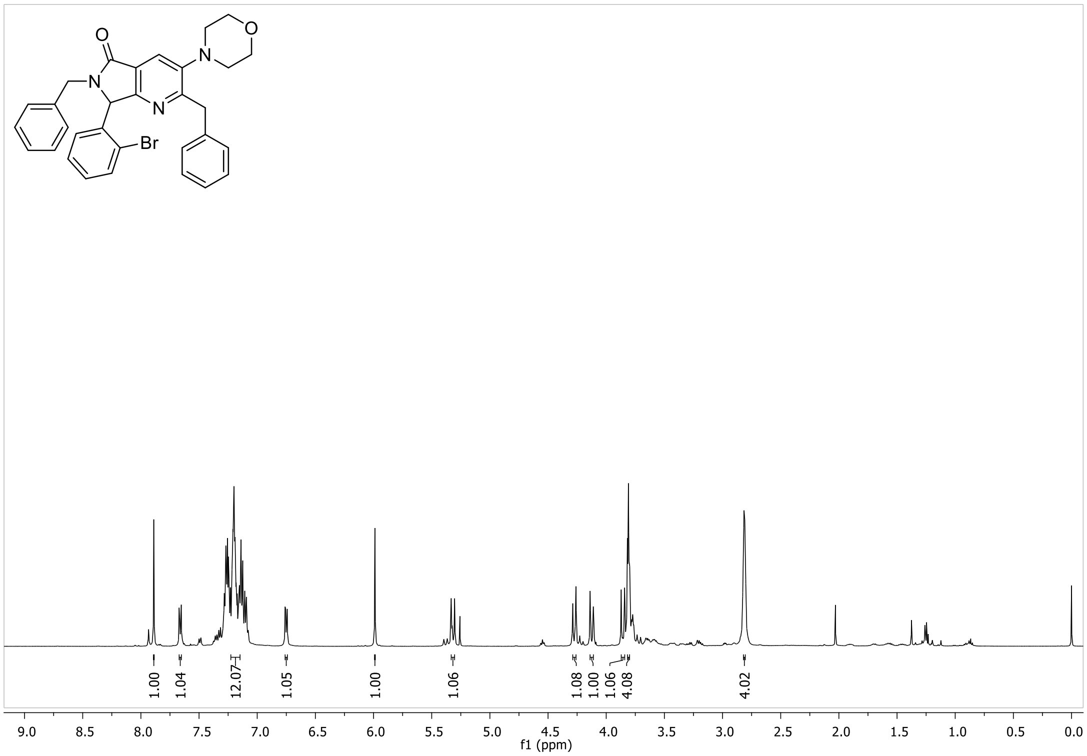

Figure S3. ^{1}H NMR spectrum of the product **11b**

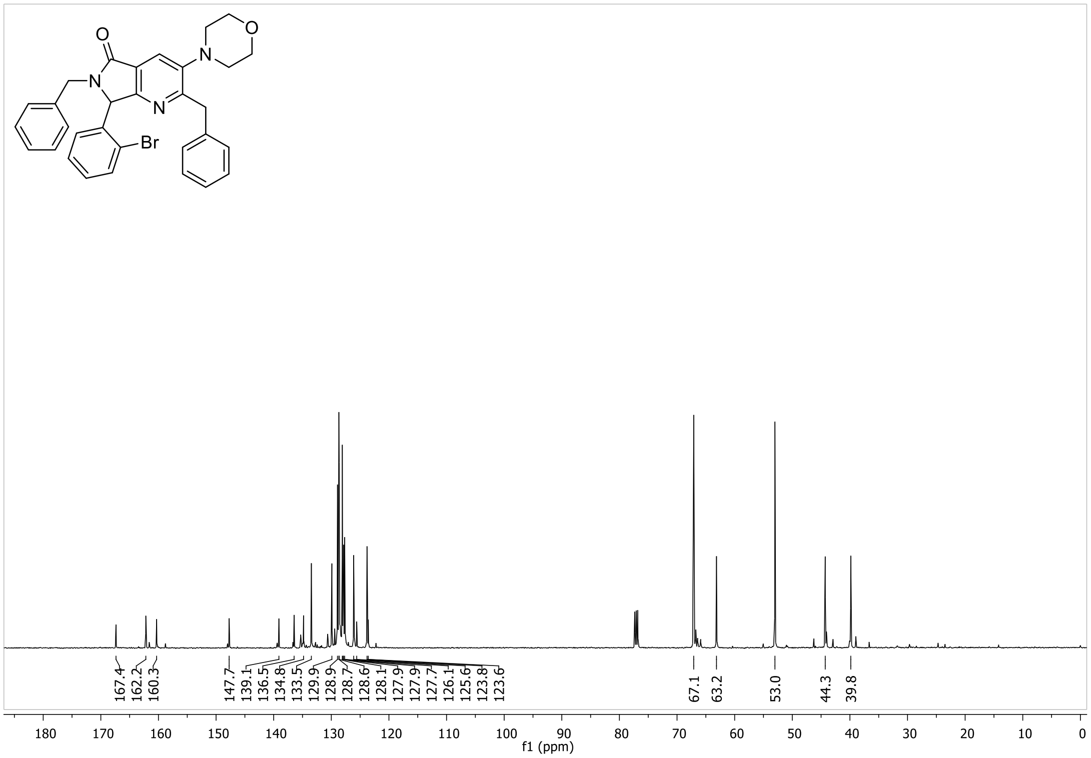

Figure S4. ^{13}C NMR spectrum of the product **11b**

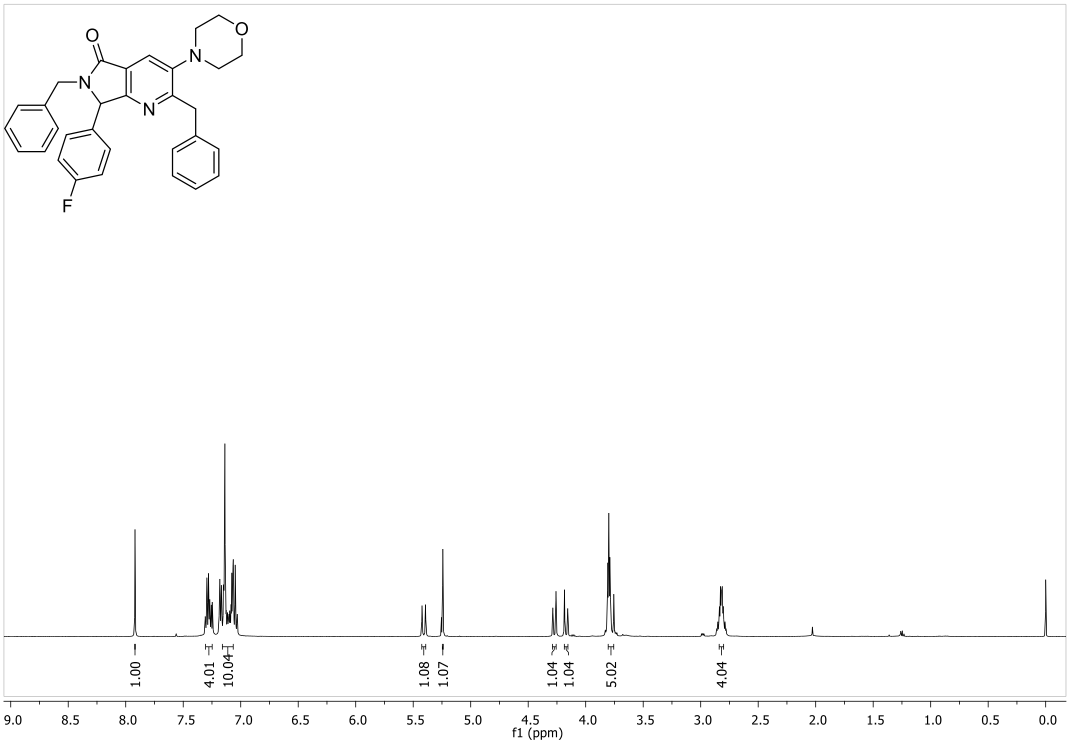

Figure S5. ^{1}H NMR spectrum of the product **11c**

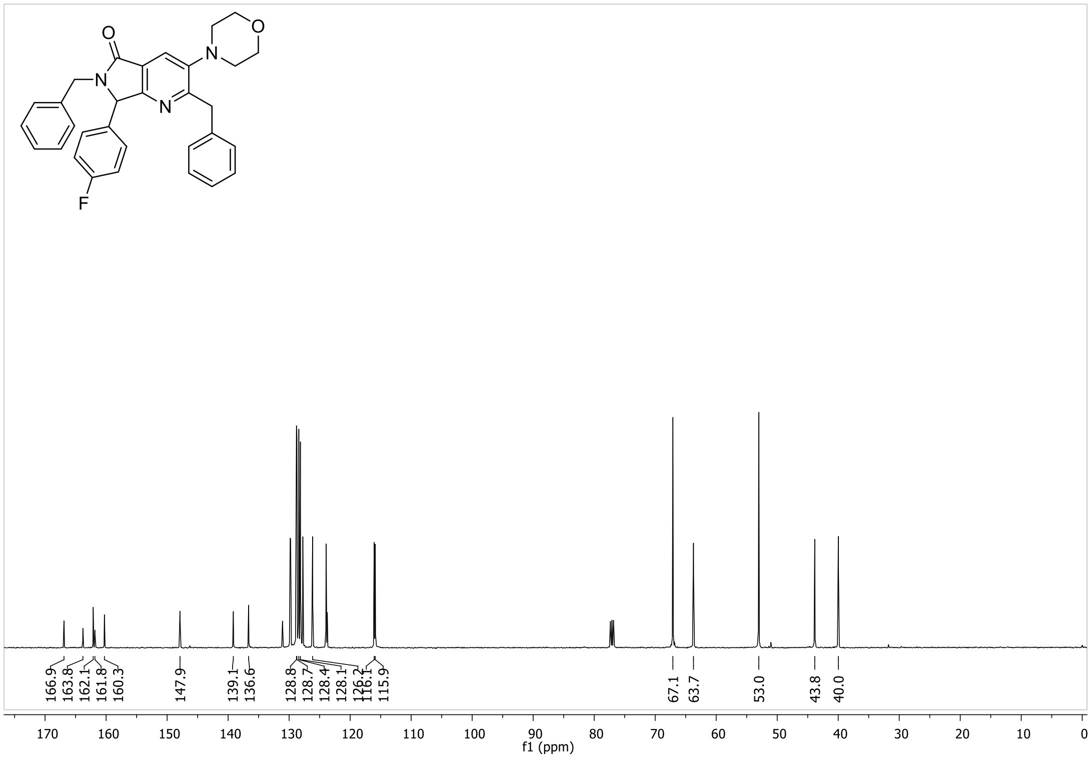

Figure S6. ^{13}C NMR spectrum of the product **11c**

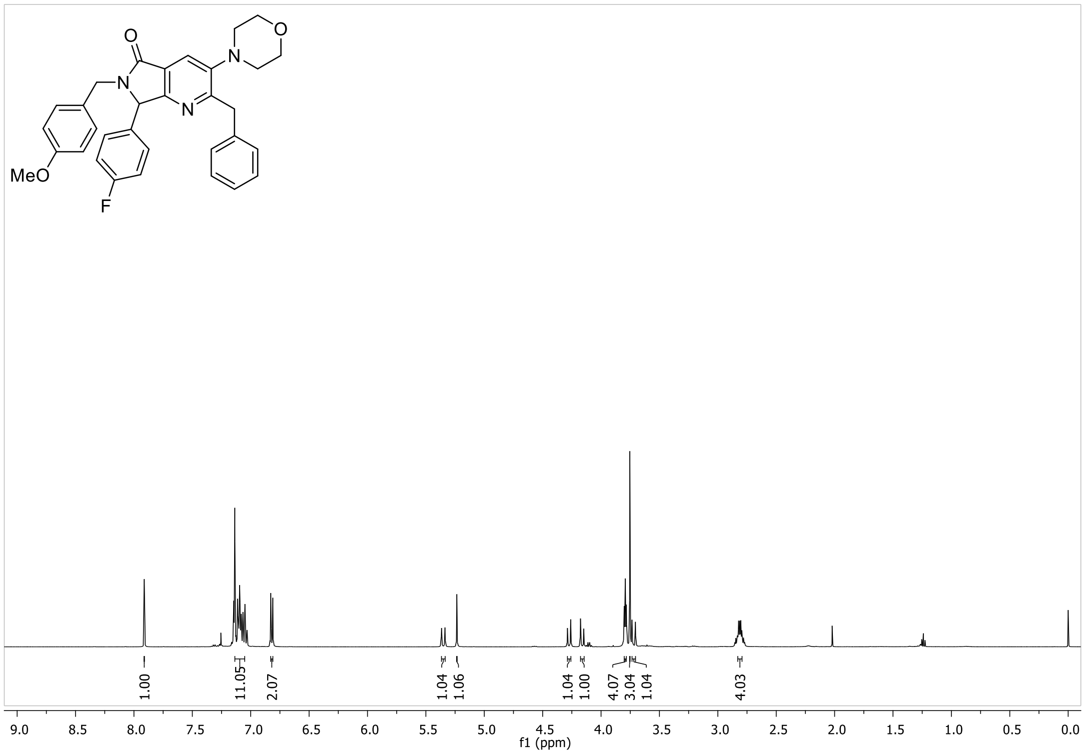

Figure S7. ^{1}H NMR spectrum of the product **11d**

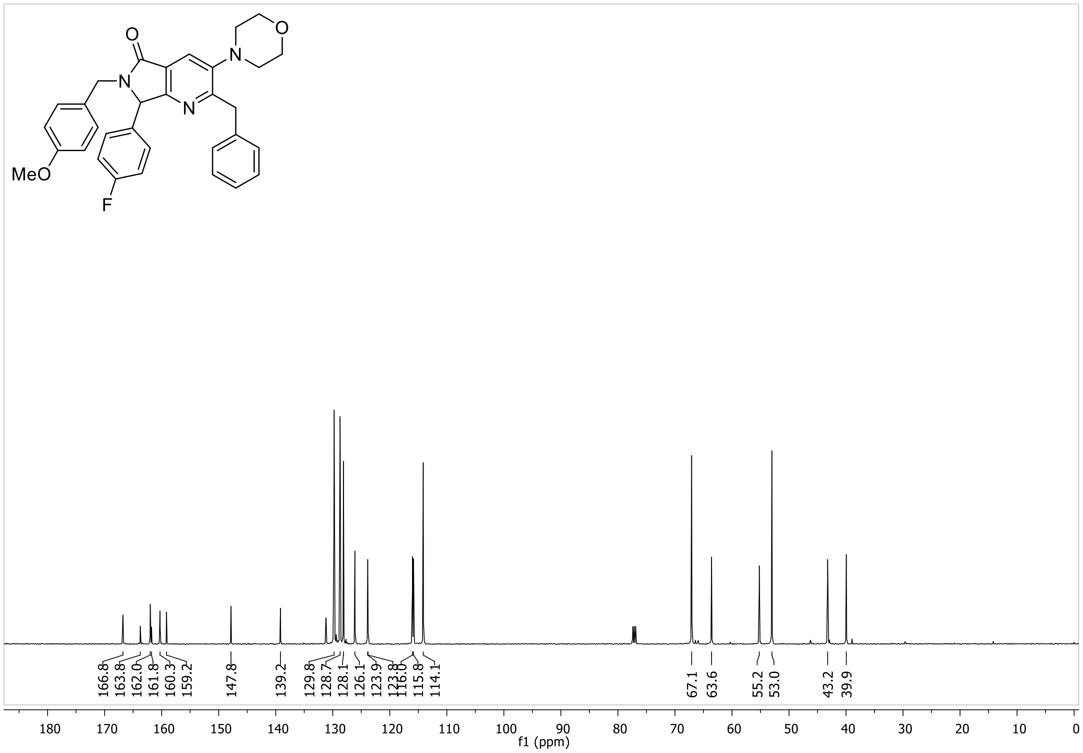

Figure S8. ^{13}C NMR spectrum of the product **11d**

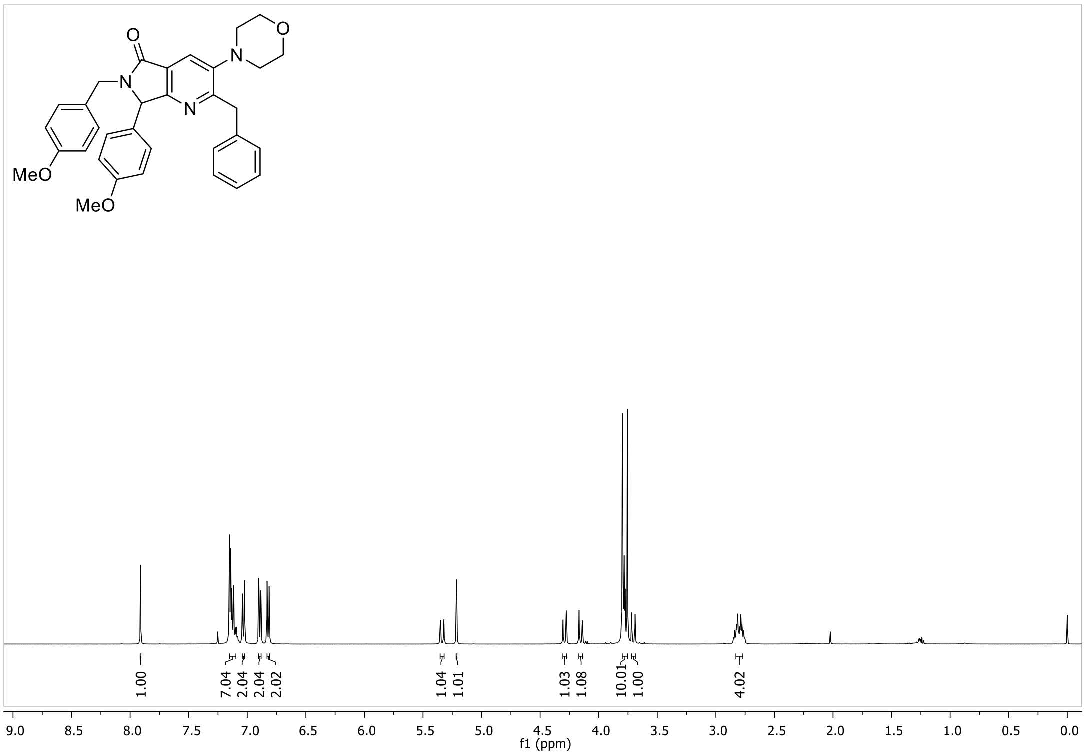

Figure S9. ^{1}H NMR spectrum of the product **11e**

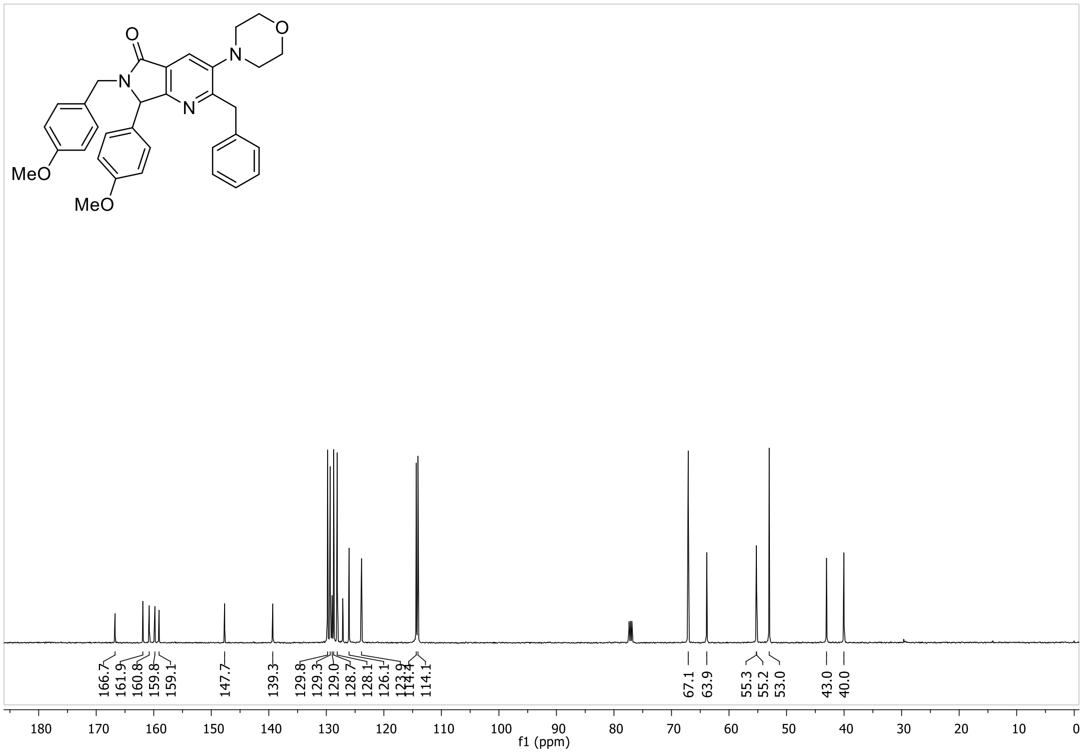

Figure S10. ^{13}C NMR spectrum of the product **11e**

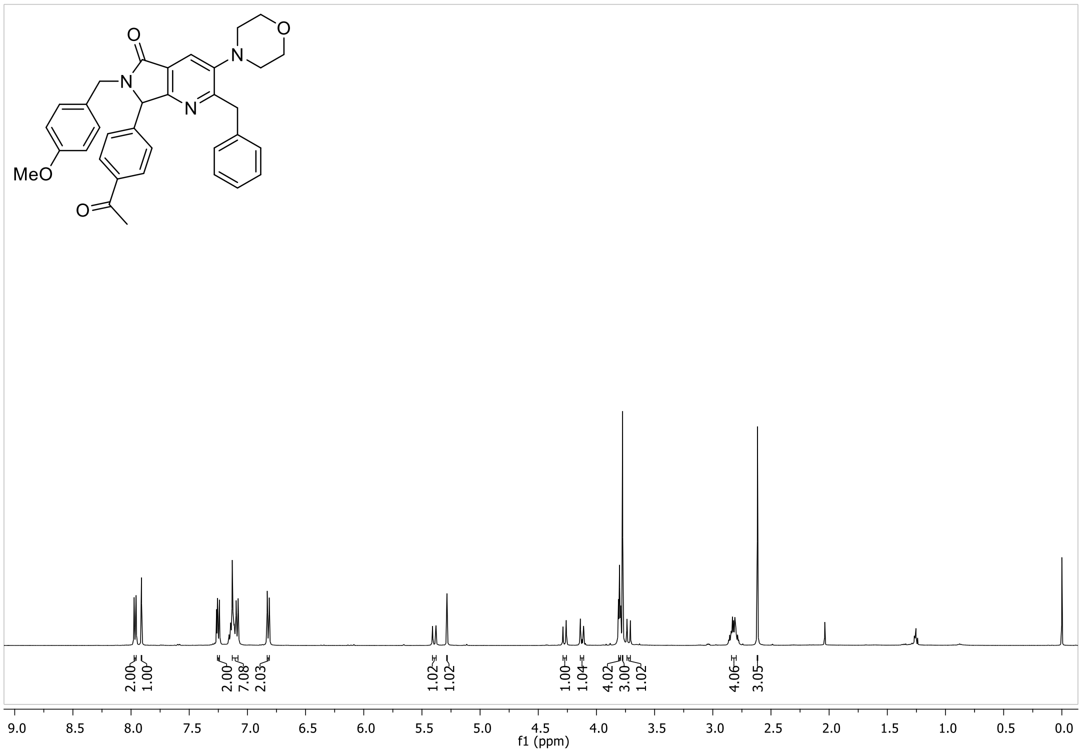

Figure S11. ^{1}H NMR spectrum of the product **11f**

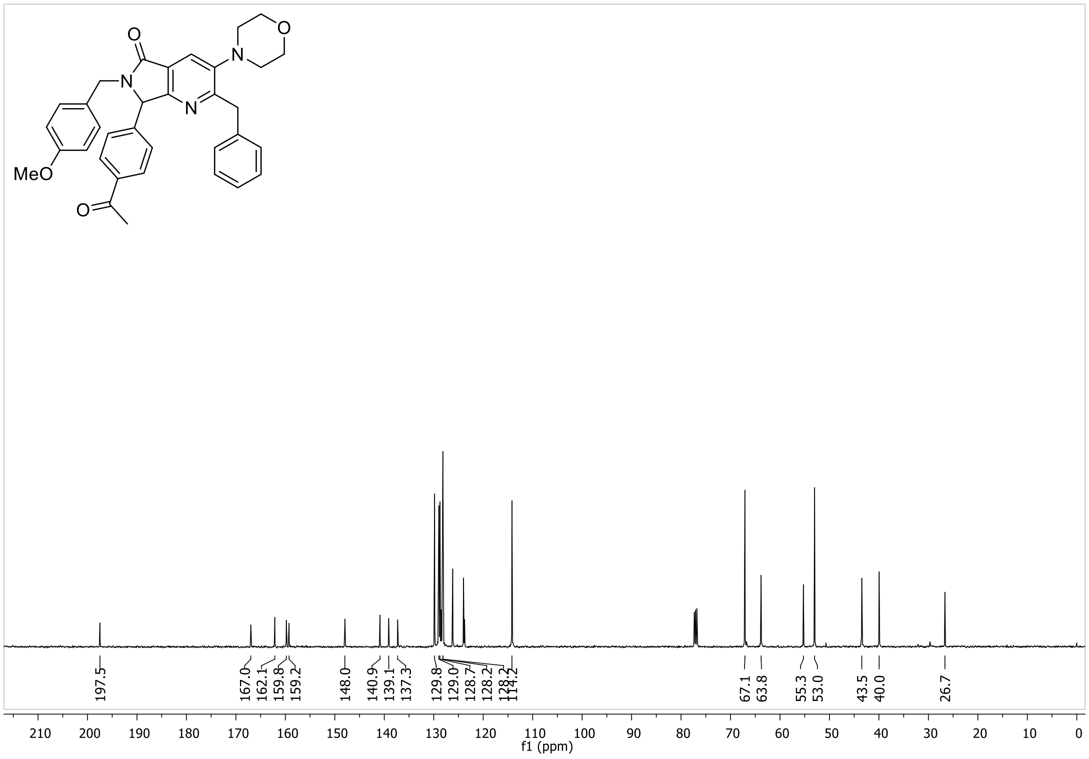

Figure S12. ^{13}C NMR spectrum of the product **11f**

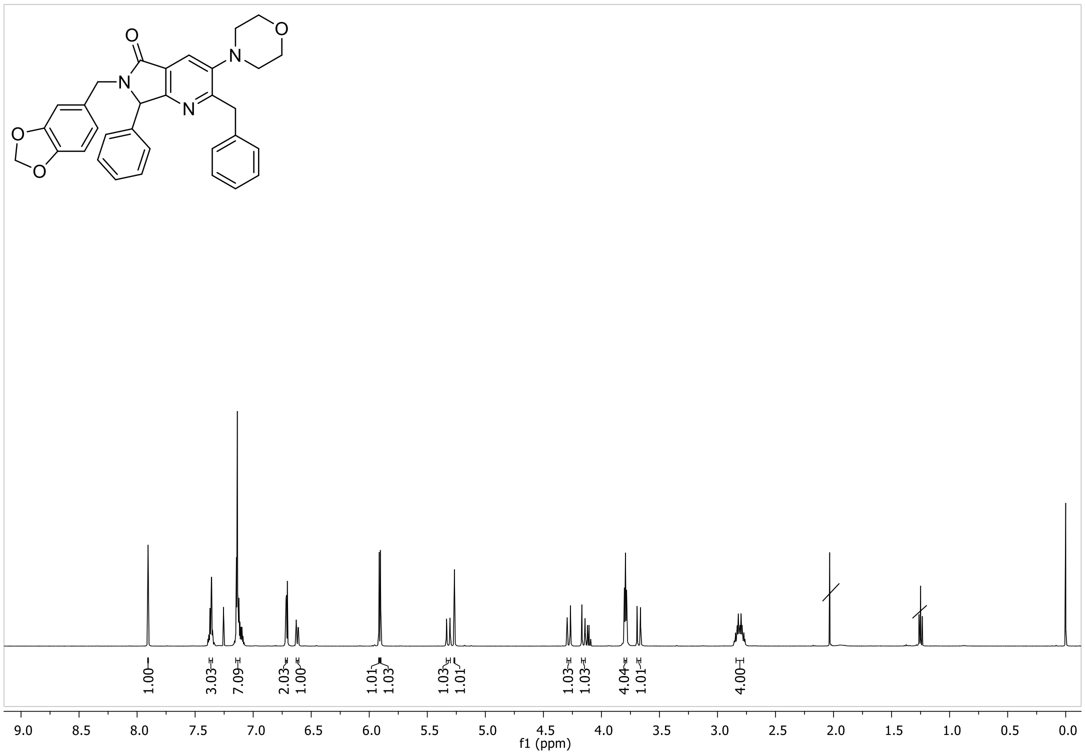

Figure S11. ^{1}H NMR spectrum of the product **11g**

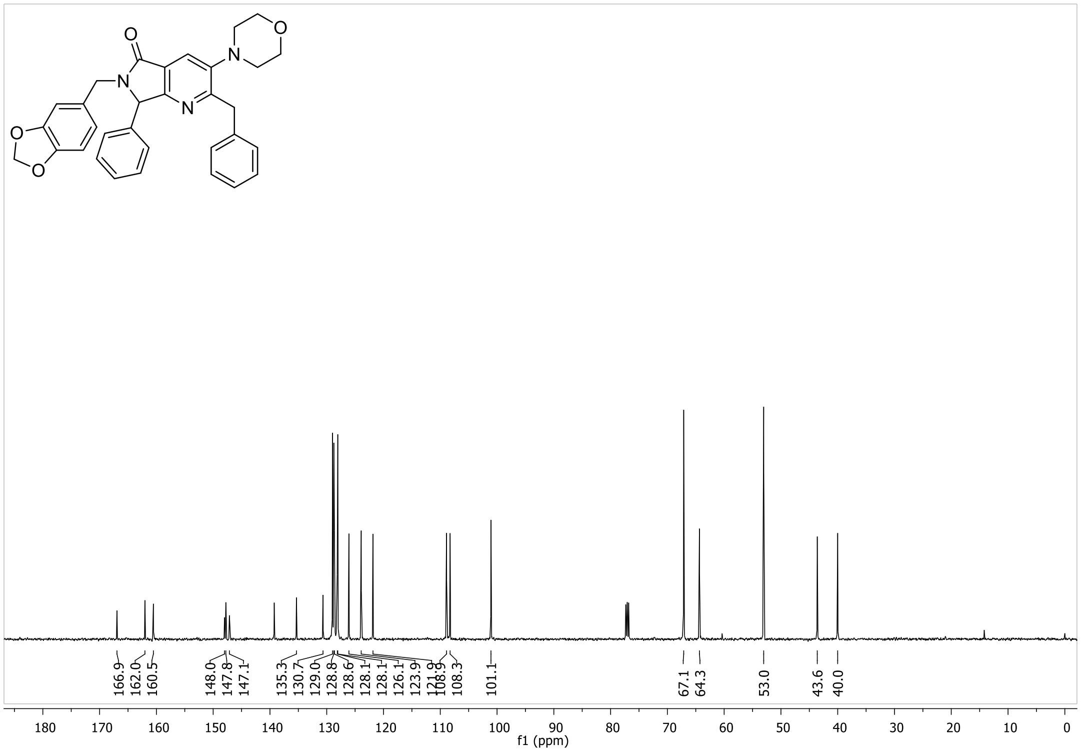

Figure S14. ^{13}C NMR spectrum of the product **11g**

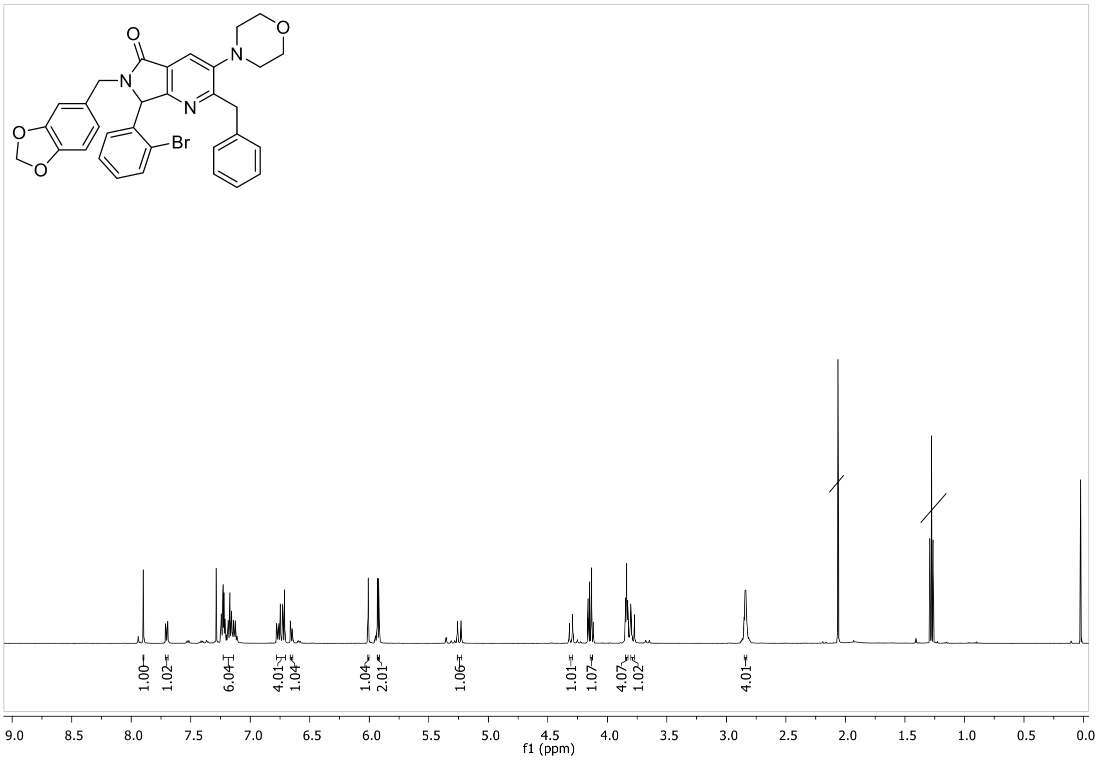

Figure S15. ^{1}H NMR spectrum of the product **11h**

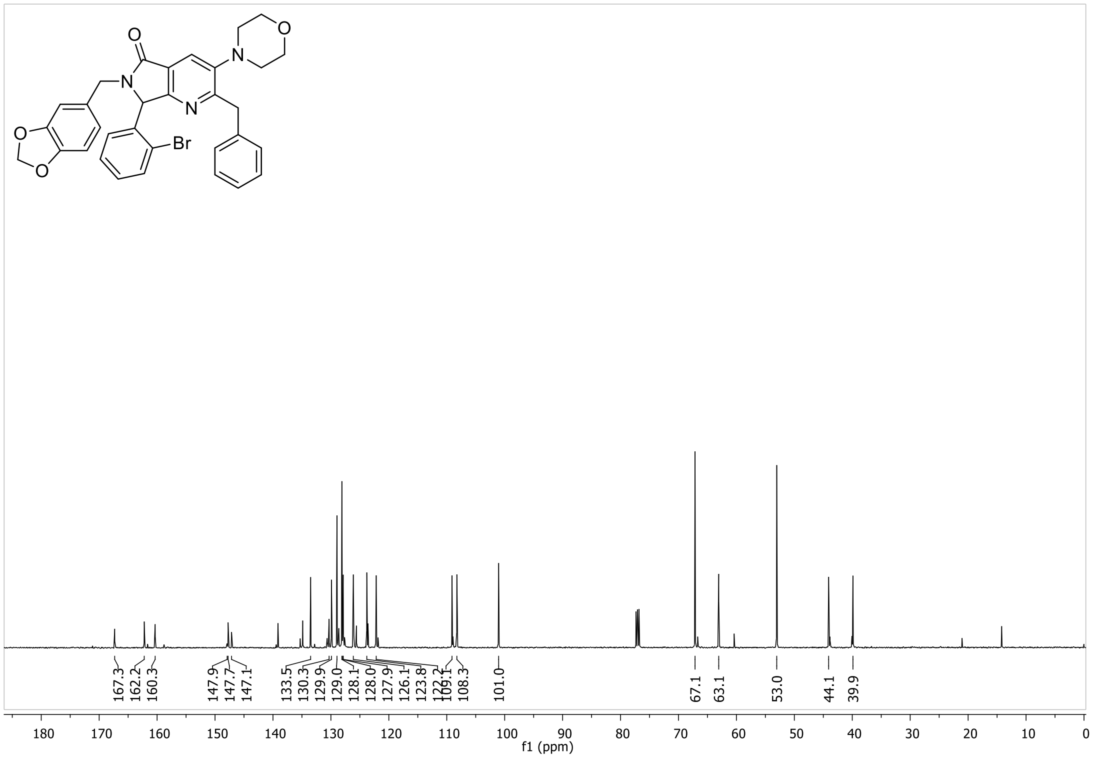

Figure S16. ^{13}C NMR spectrum of the product **11h**

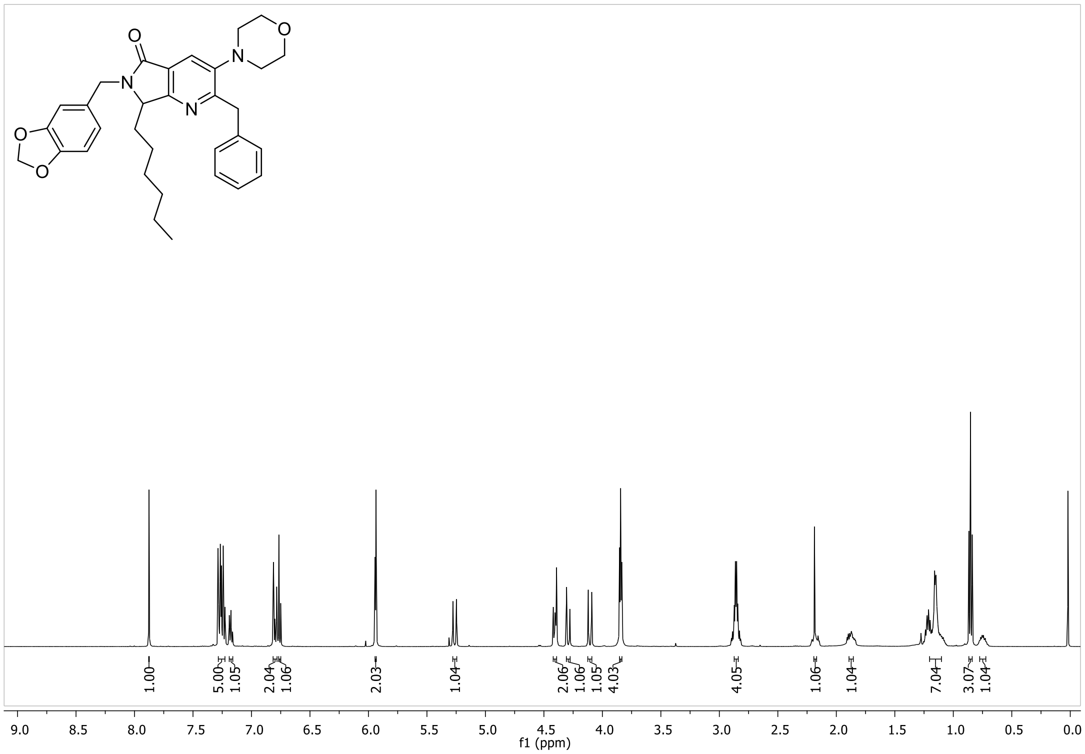

Figure S17. ^{1}H NMR spectrum of the product **11i**

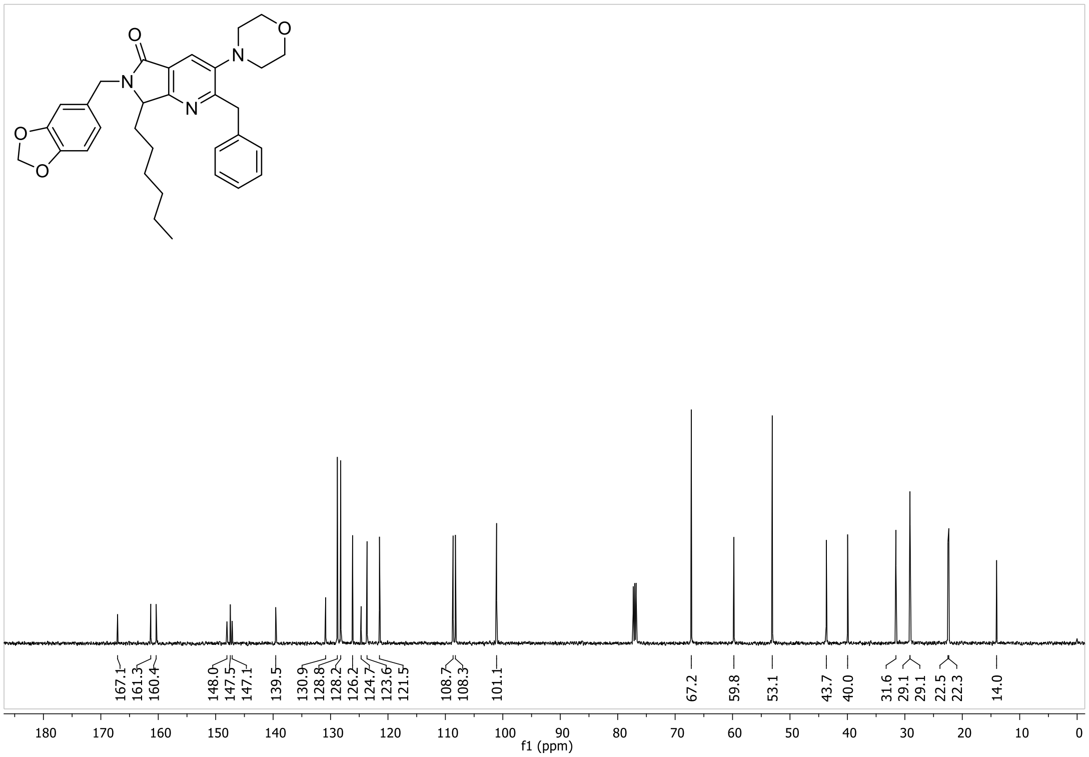

Figure S18. ^{13}C NMR spectrum of the product **11i**

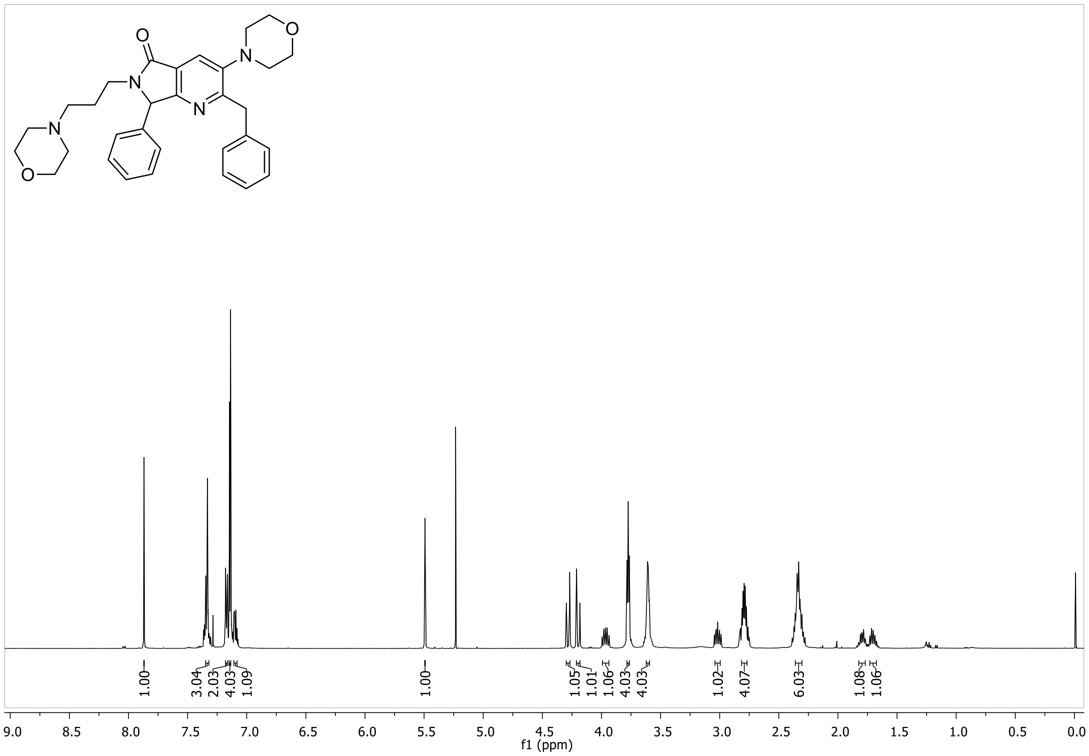

Figure S19. ^{1}H NMR spectrum of the product **11j**

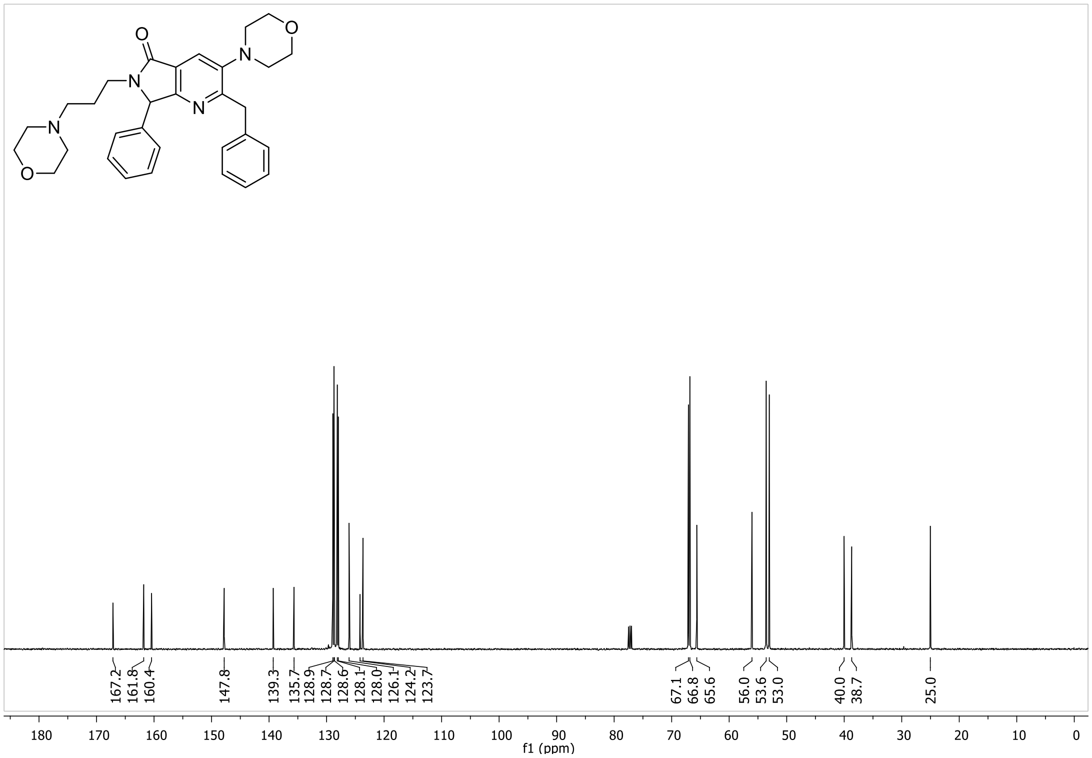

Figure S20. ^{13}C NMR spectrum of the product **11j**

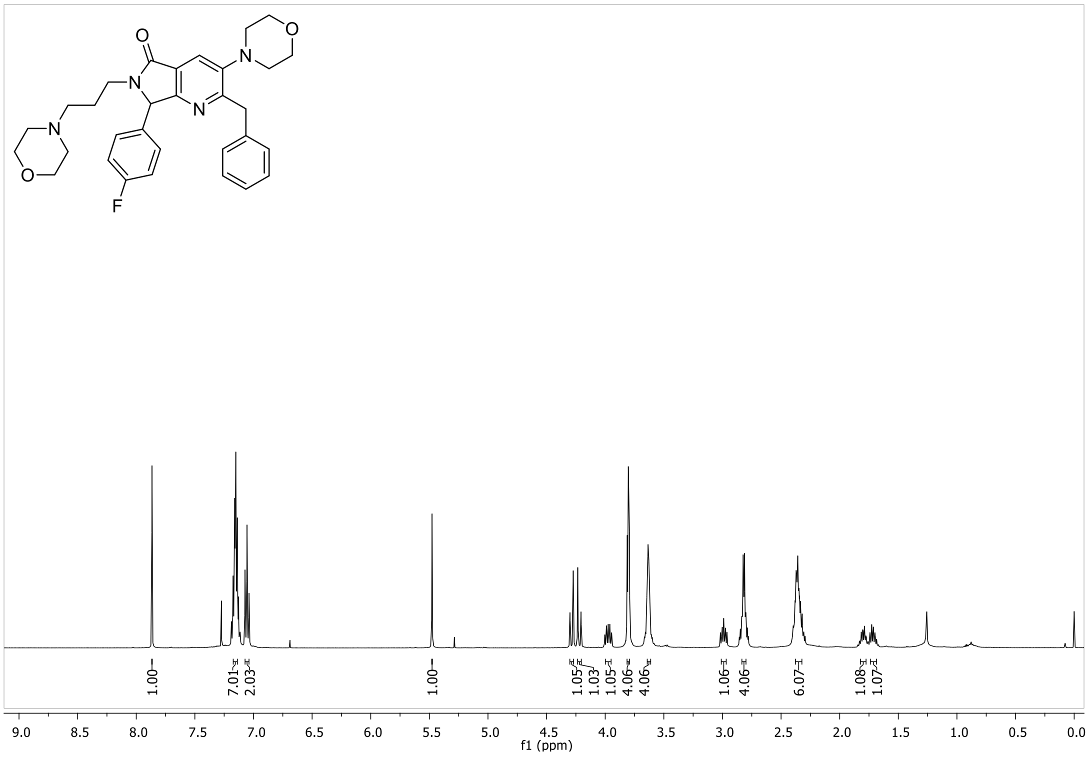

Figure S21. ¹H NMR spectrum of the product **11k**

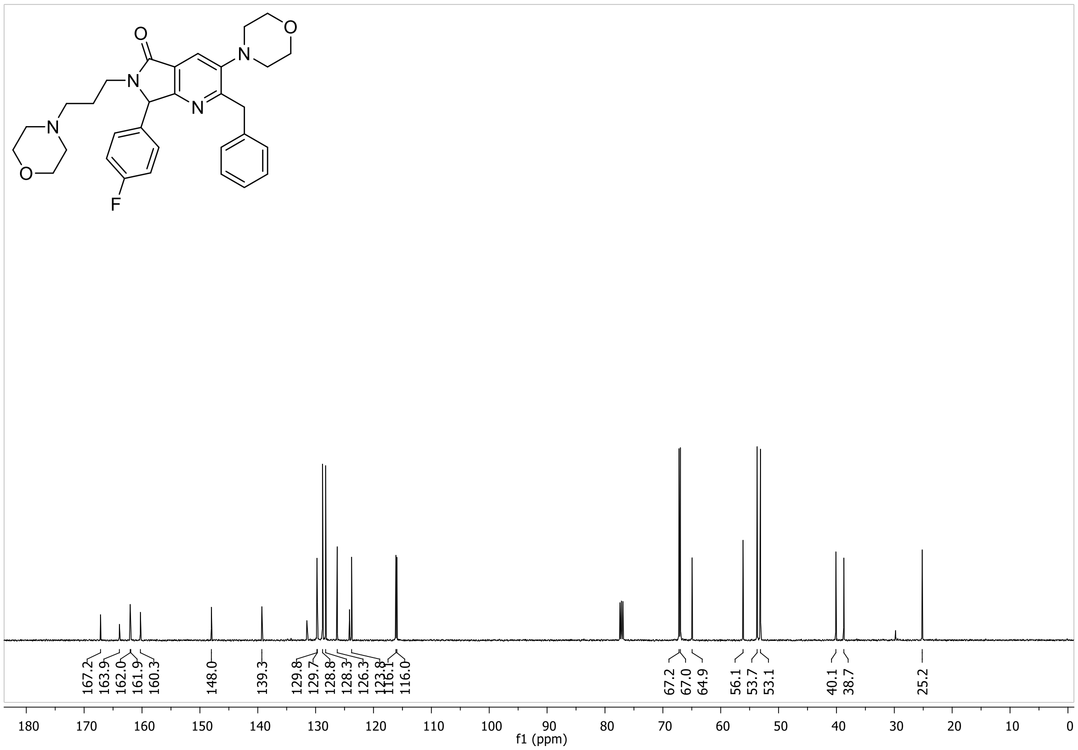

Figure S22. ¹³C NMR spectrum of the product **11k**

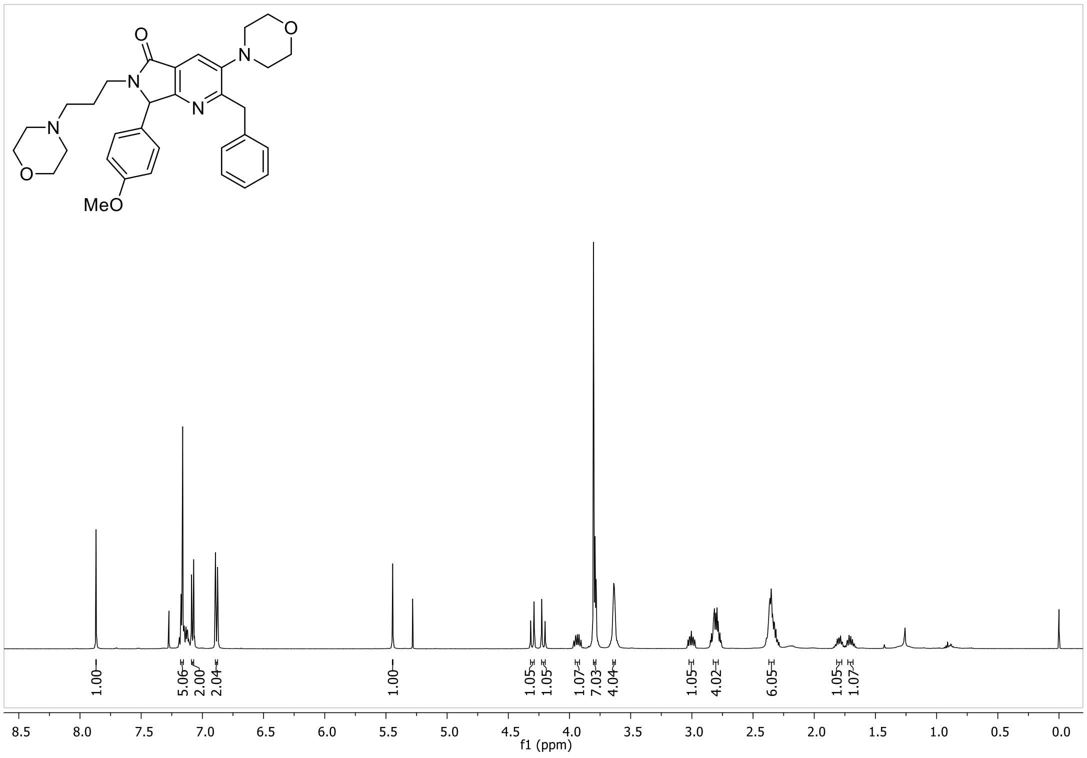

Figure S23. ^{1}H NMR spectrum of the product **11l**

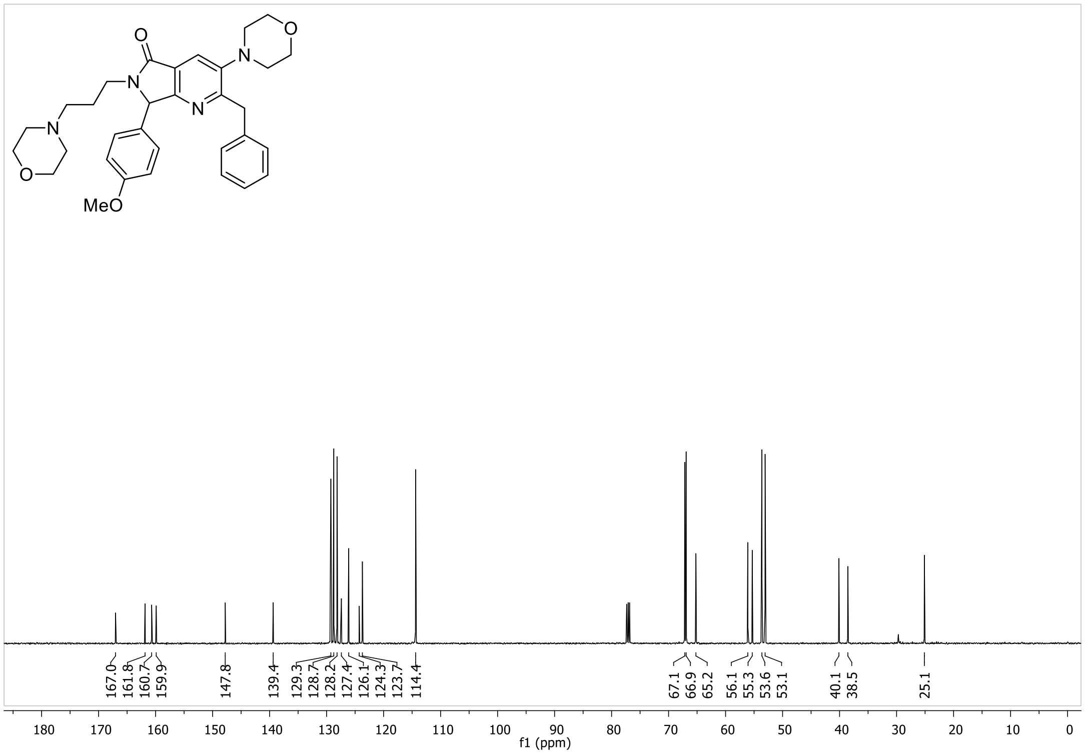

Figure S24. ^{13}C NMR spectrum of the product **11l**

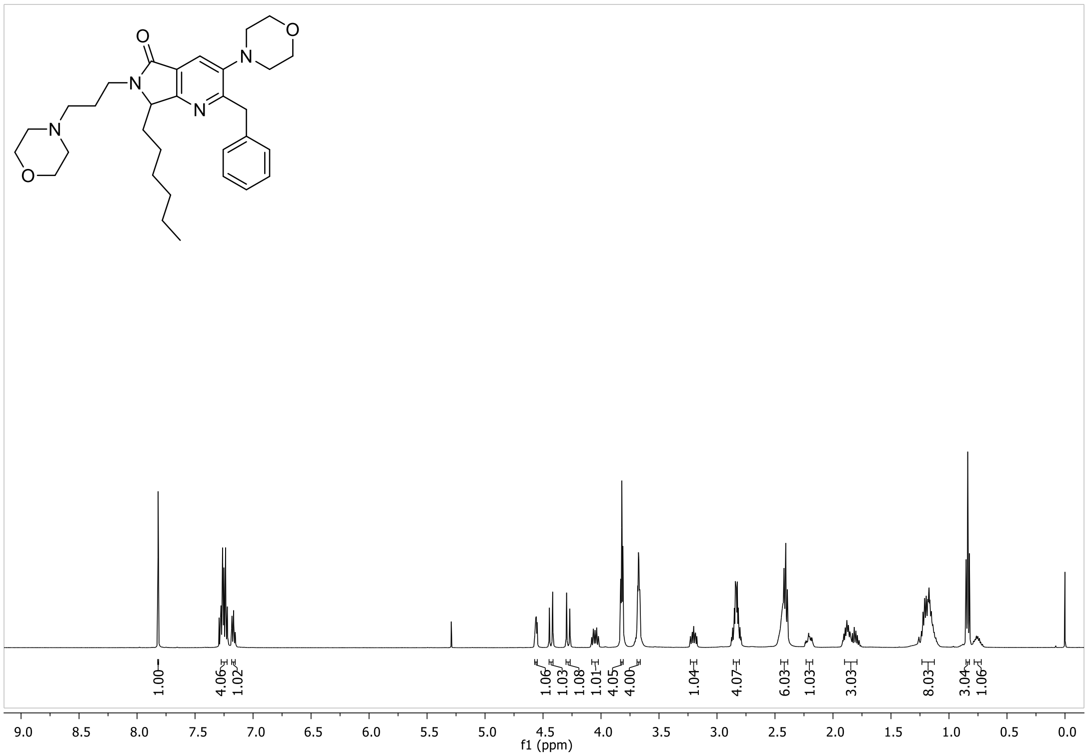

Figure S25. ^{1}H NMR spectrum of the product **11m**

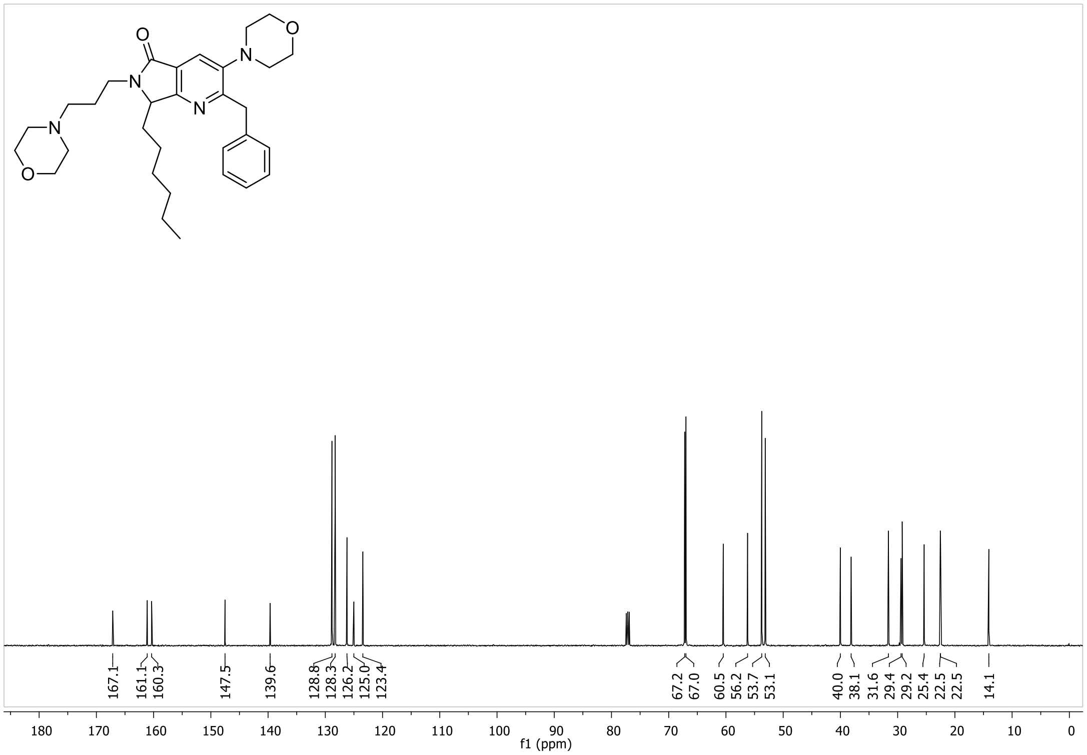

Figure S26. ^{13}C NMR spectrum of the product **11m**

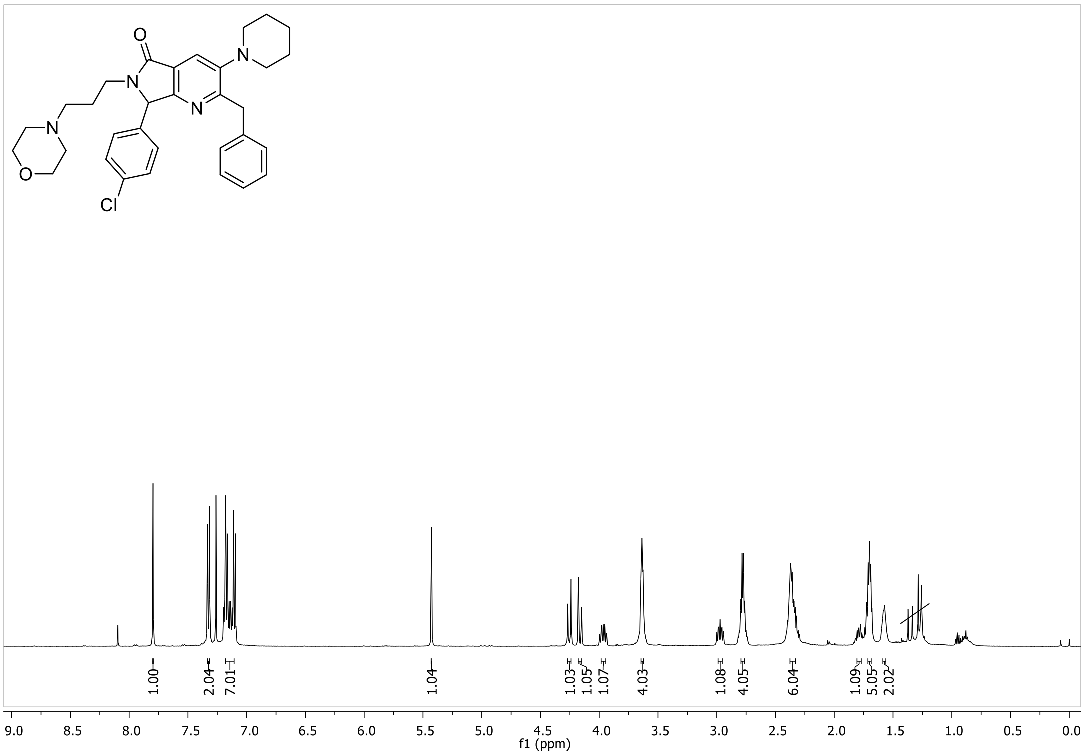

Figure S27. ^{1}H NMR spectrum of the product **11n**

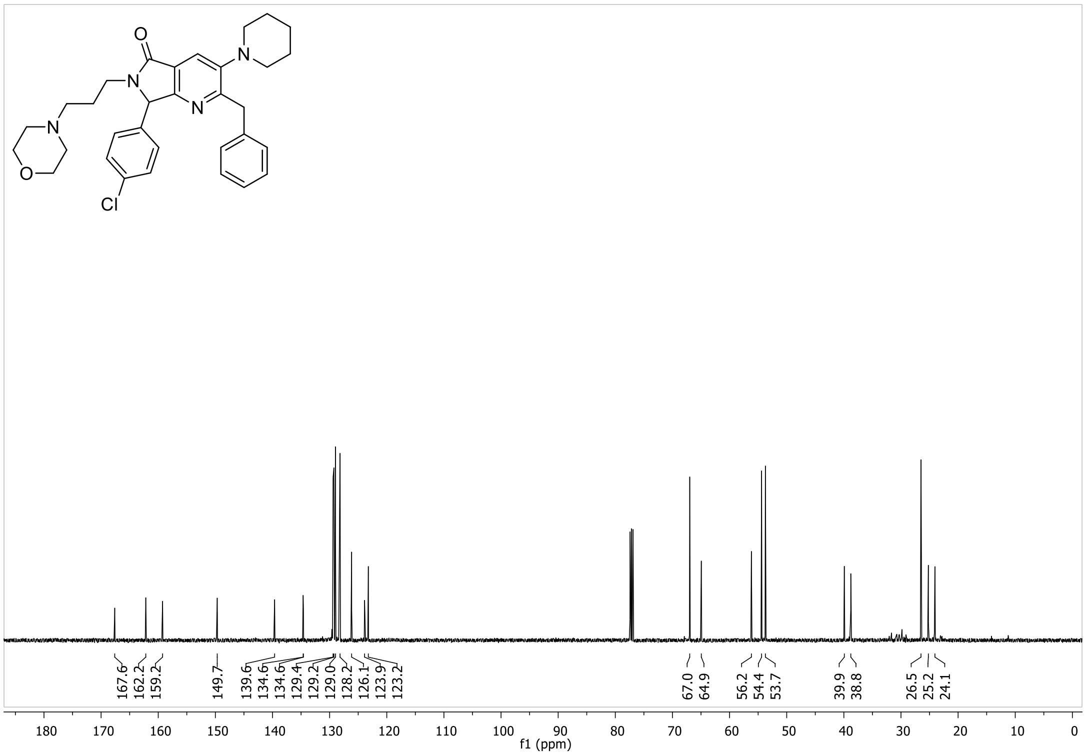

Figure S28. ^{13}C NMR spectrum of the product **11n**

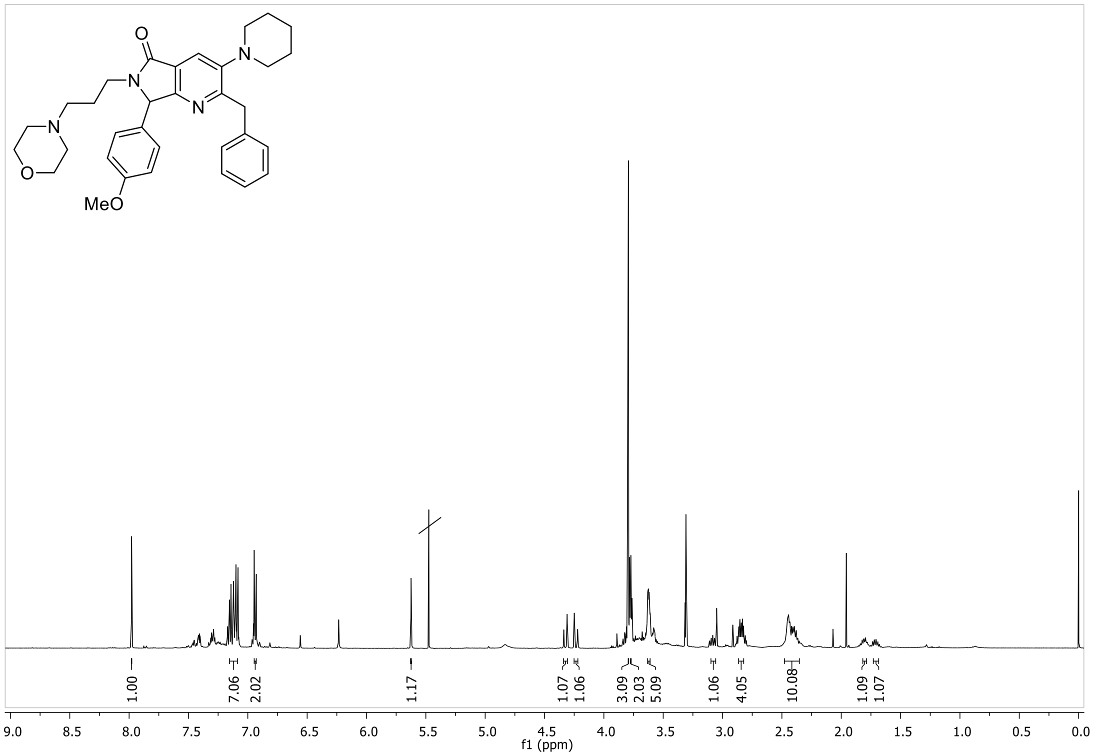

Figure S29. ^{1}H NMR spectrum of the product **11o**

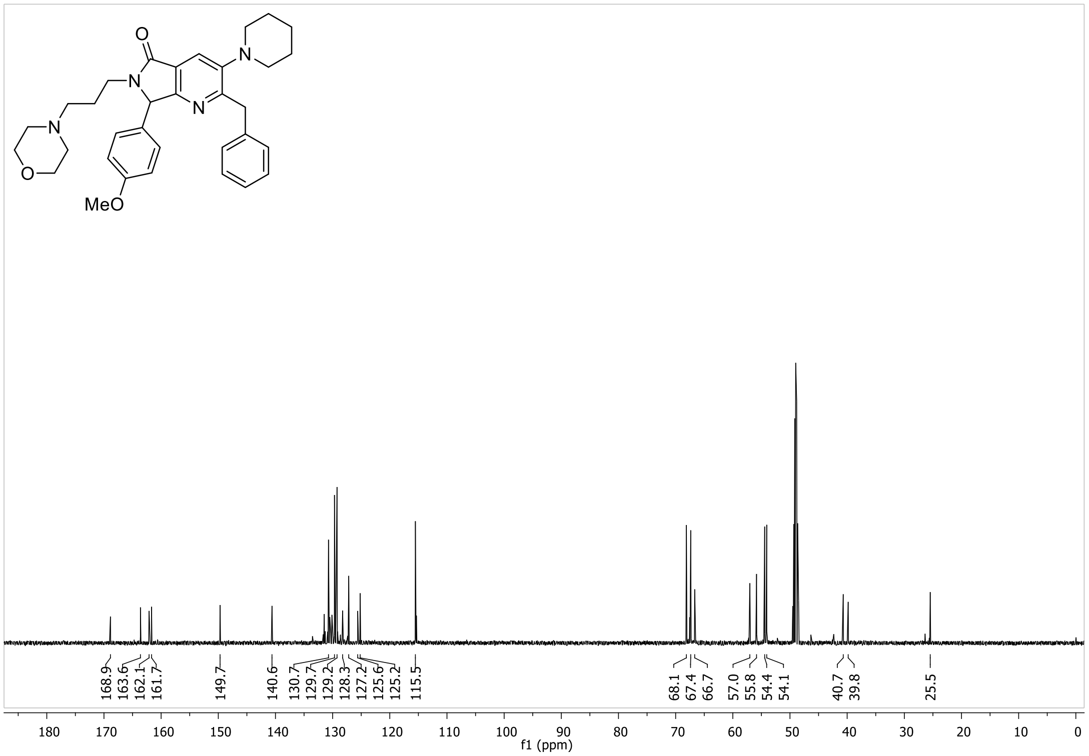

Figure S30. ^{13}C NMR spectrum of the product **11o**

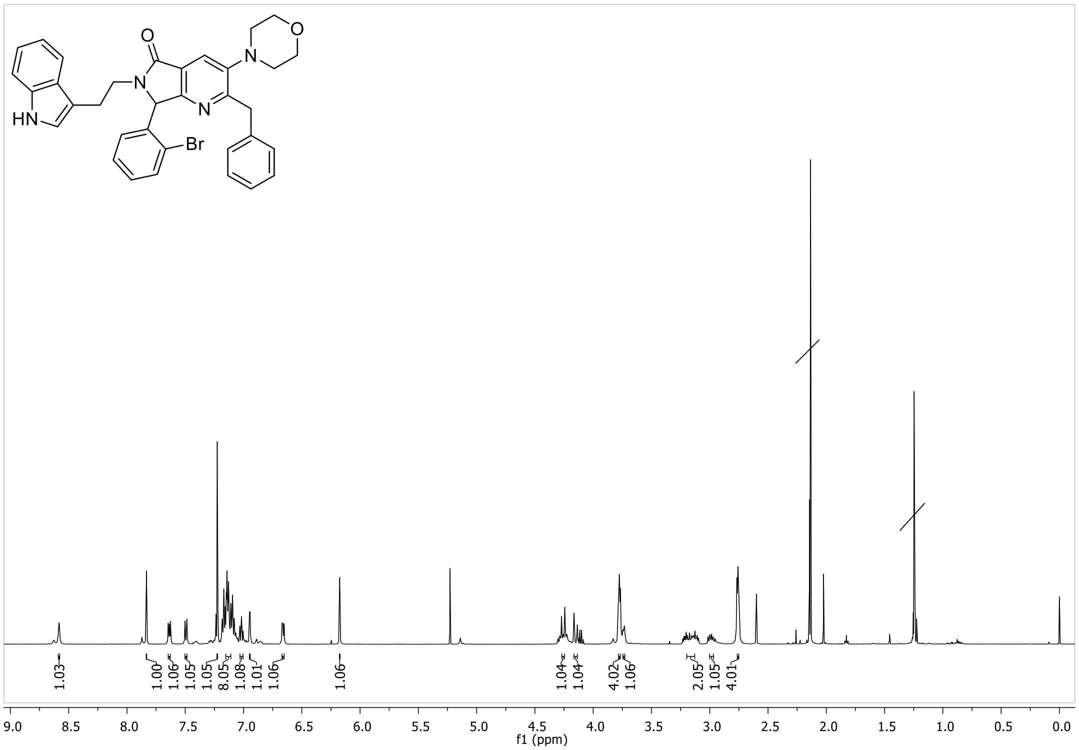

Figure S31. ^{1}H NMR spectrum of the product **11p**

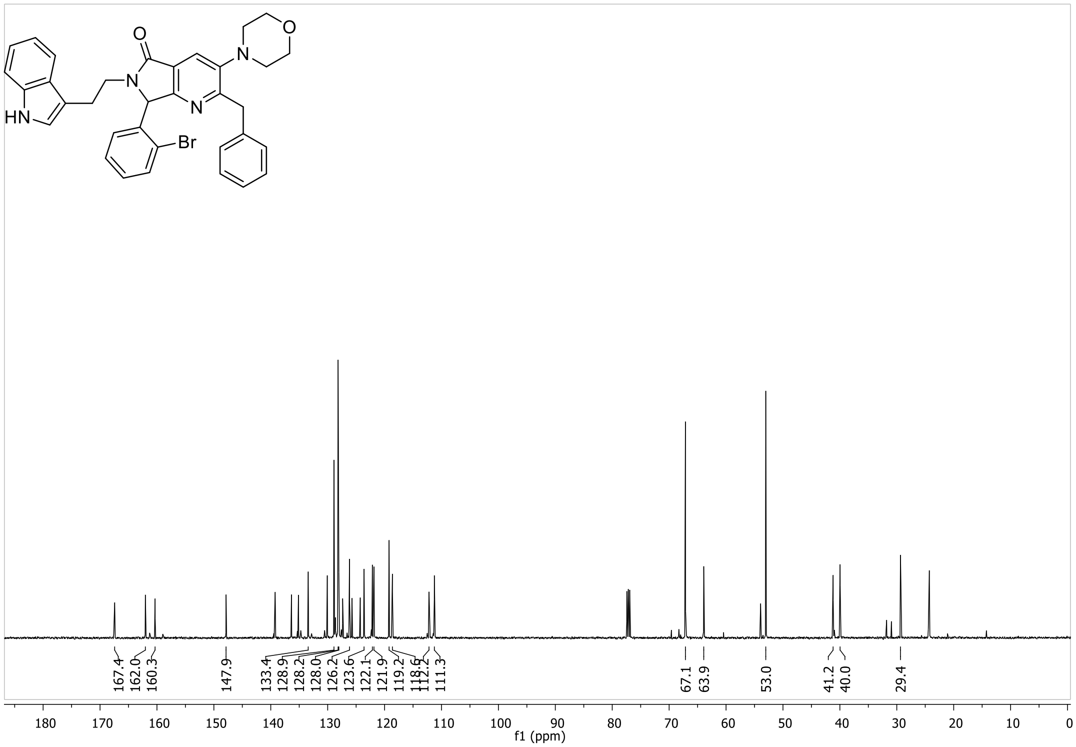

Figure S32. ^{13}C NMR spectrum of the product **11p**

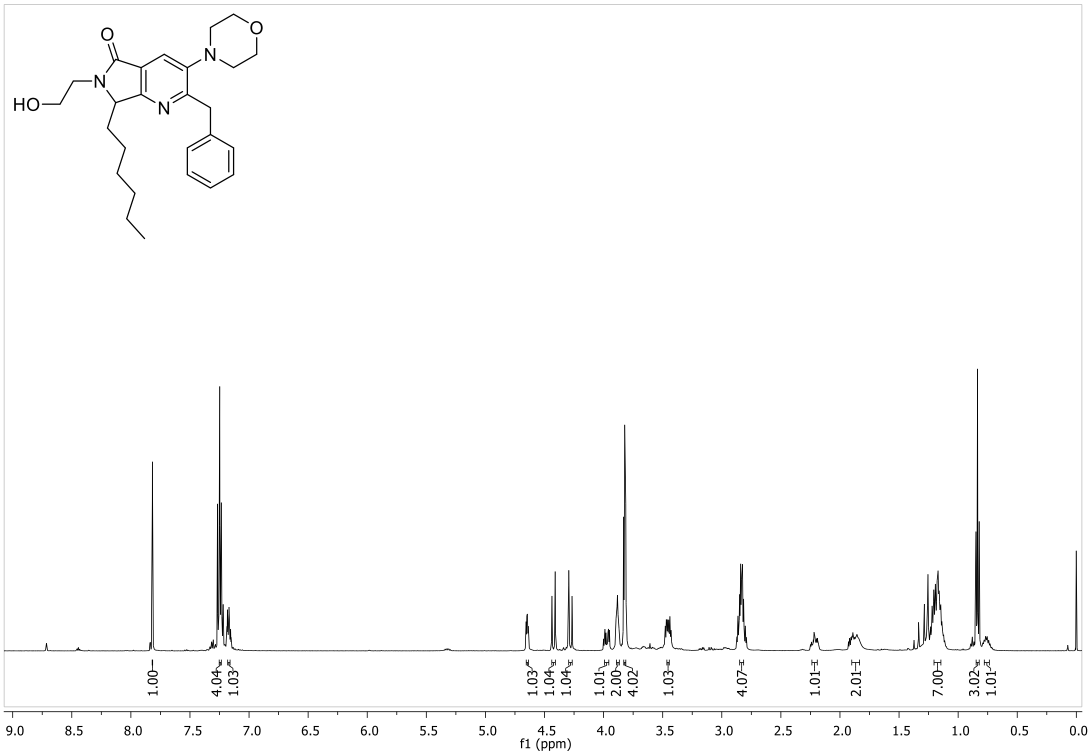

Figure S33. ^{1}H NMR spectrum of the product **11q**

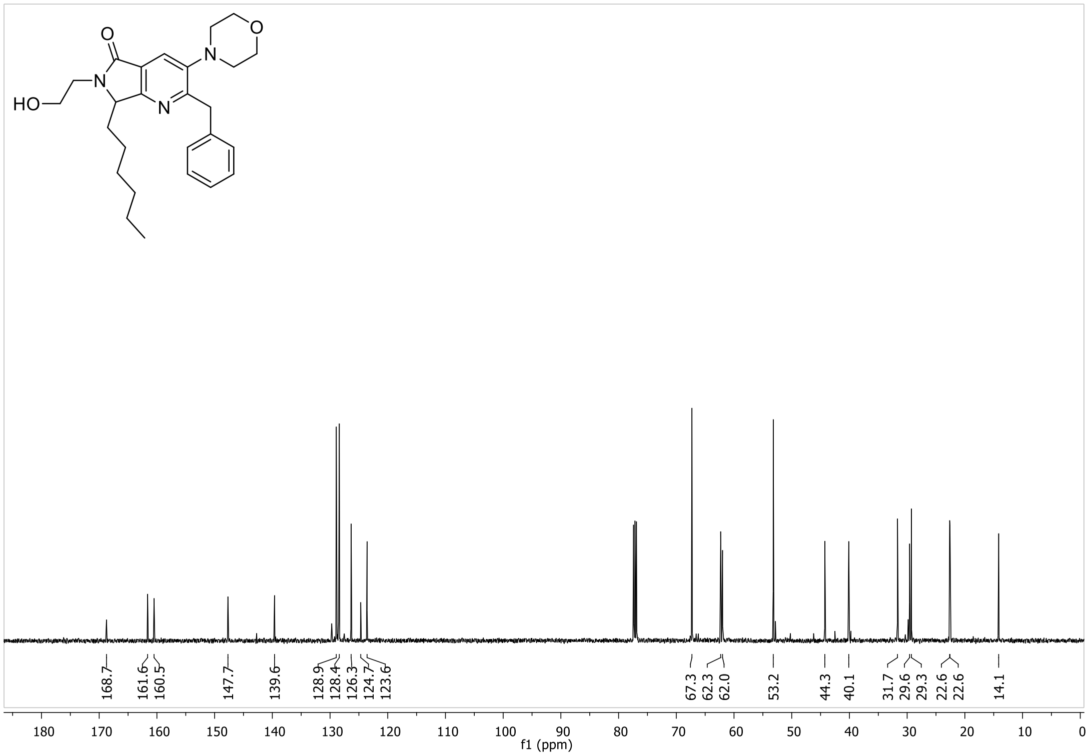

Figure S34. ^{13}C NMR spectrum of the product **11q**

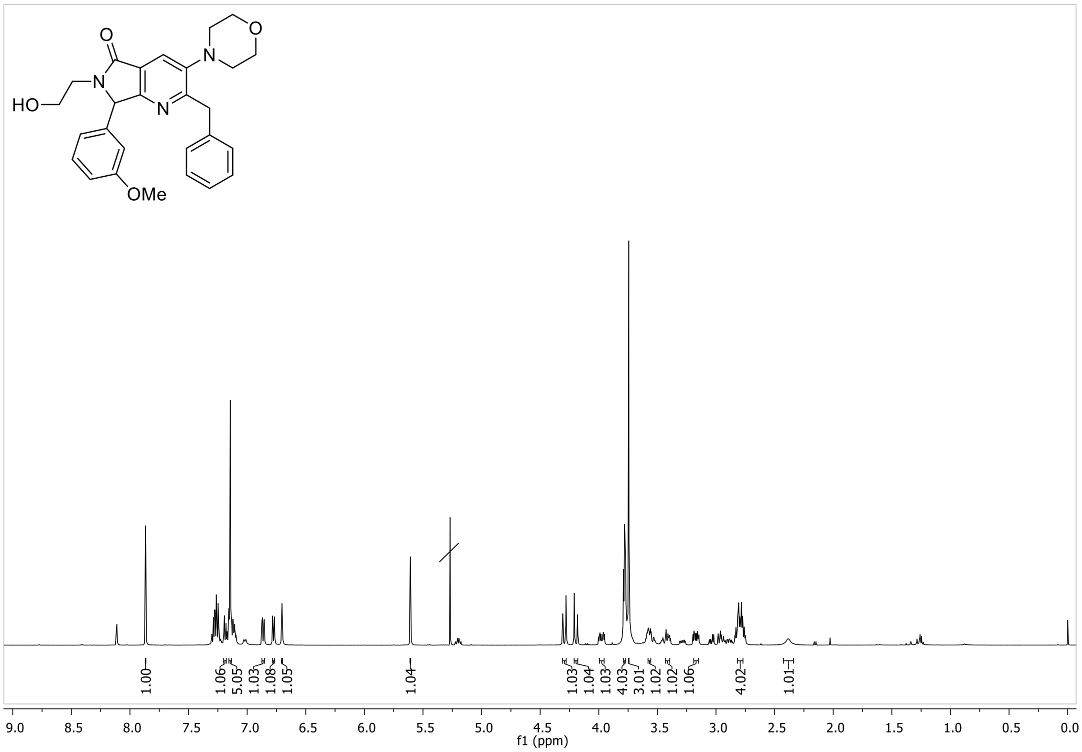

Figure S35. ^{1}H NMR spectrum of the product **11r**

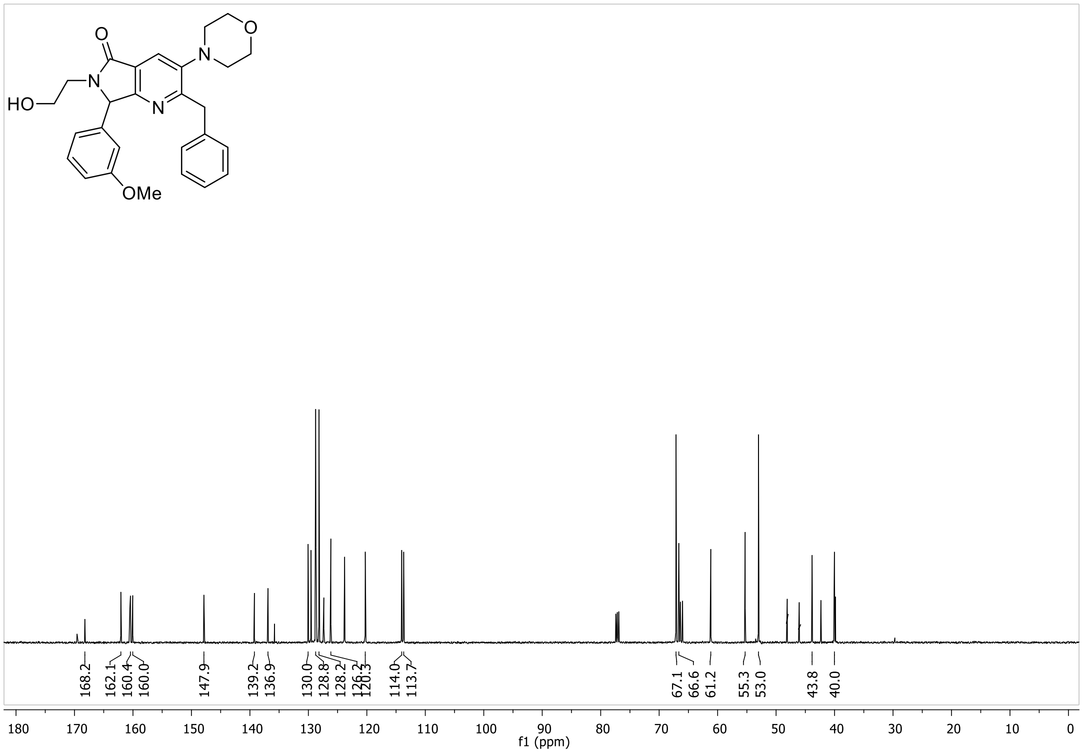

Figure S36. ^{13}C NMR spectrum of the product **11r**

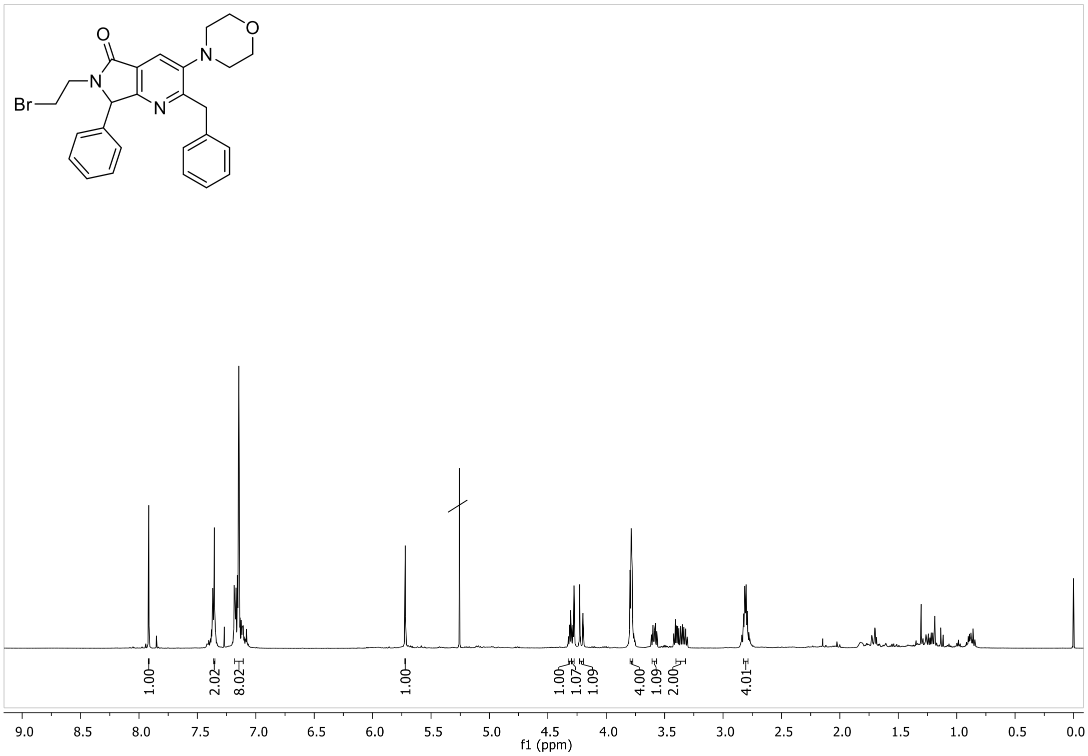

Figure S37. ^{1}H NMR spectrum of the product **11s**

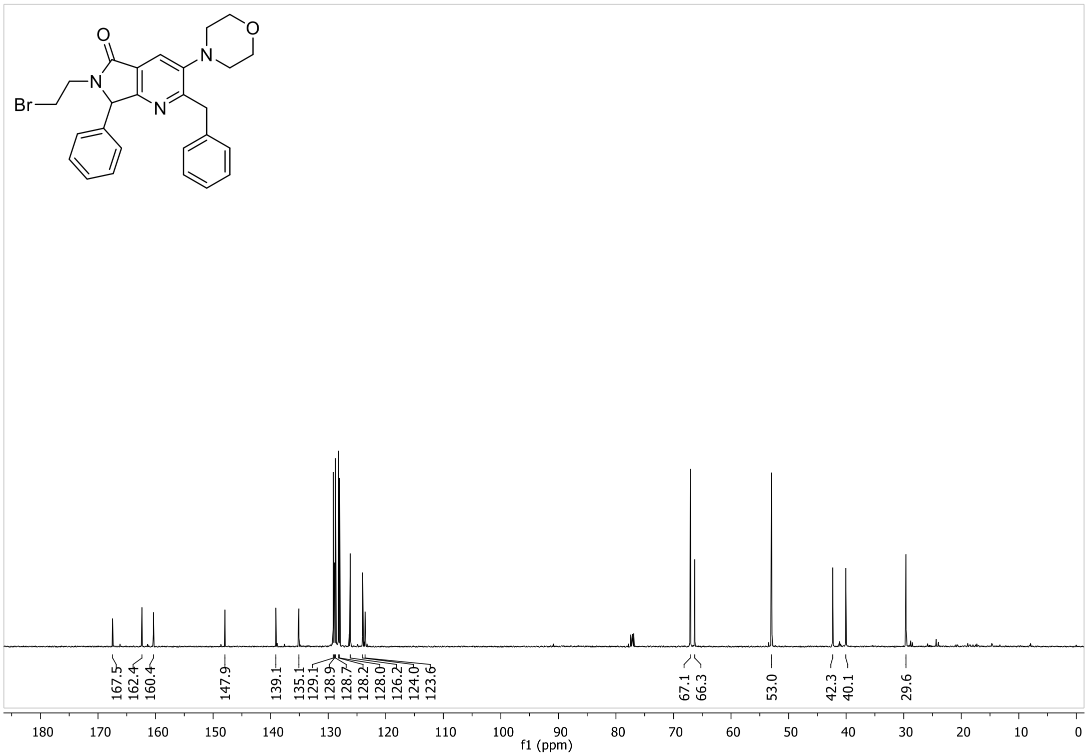

Figure S38. ^{13}C NMR spectrum of the product **11s**

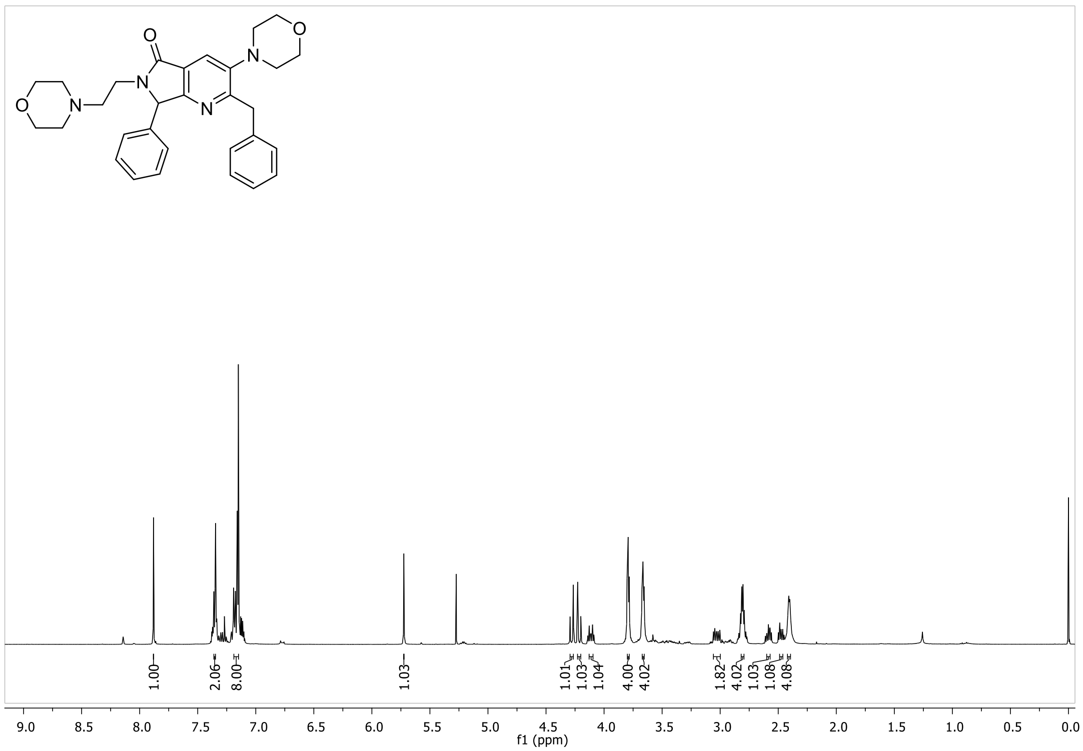

Figure S39. ^{1}H NMR spectrum of the product **11t**

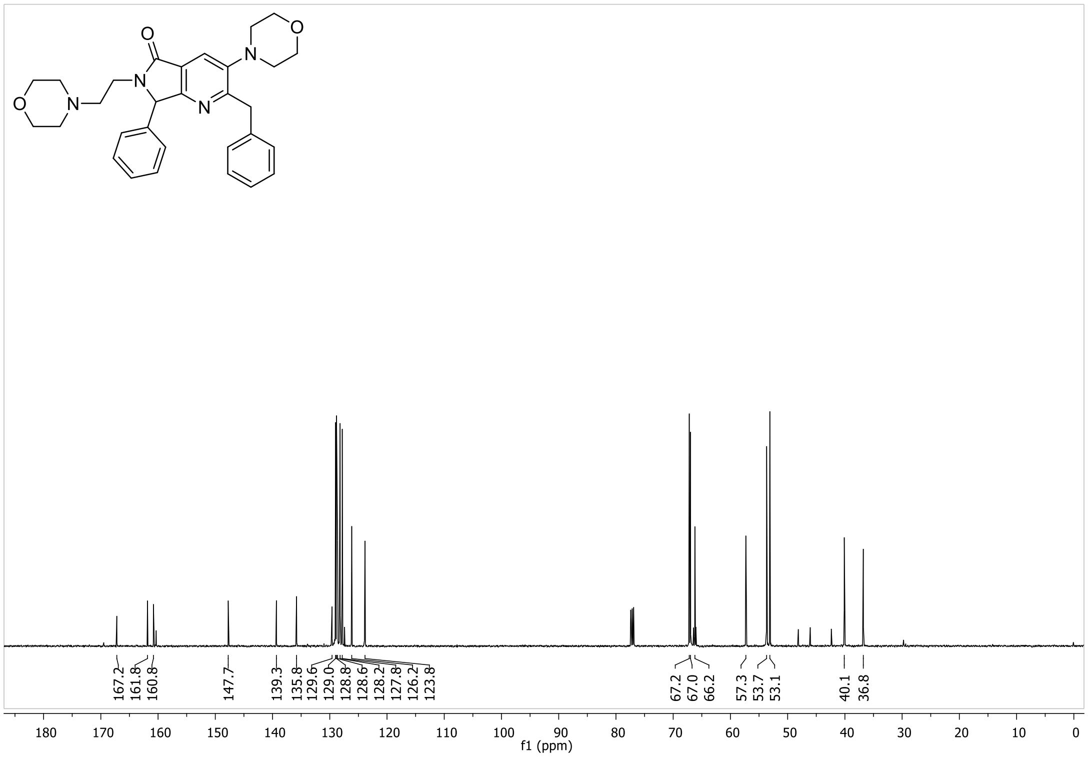

Figure S40. ^{13}C NMR spectrum of the product **11t**

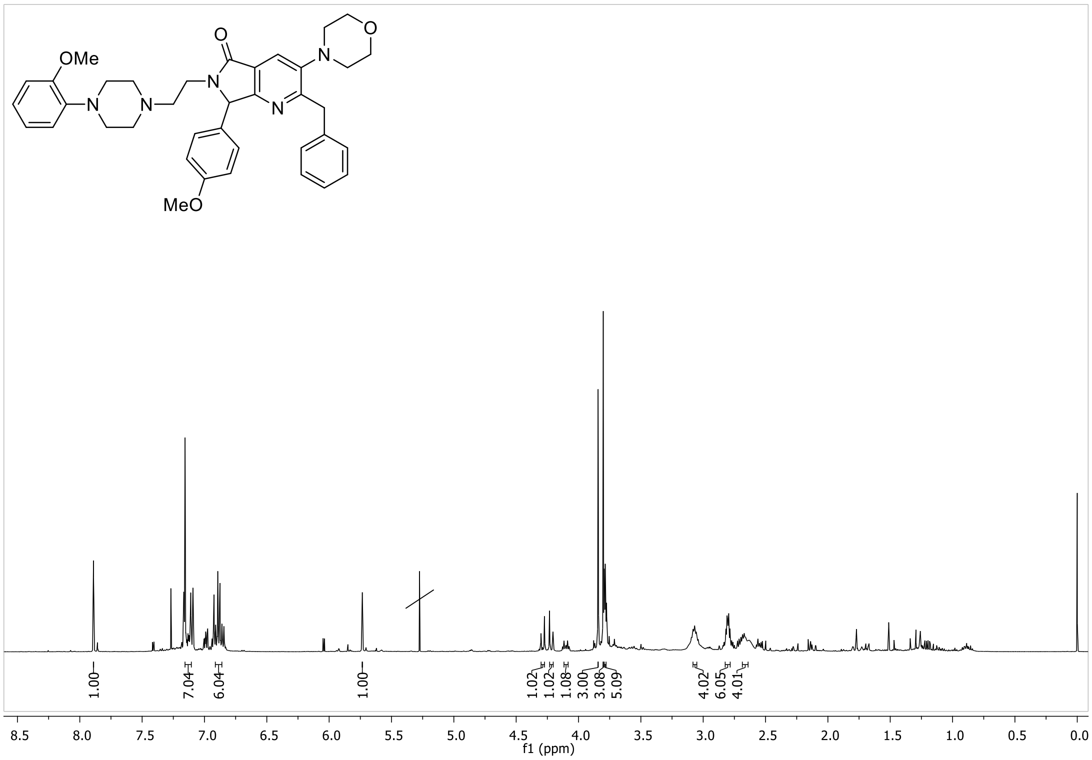

Figure S41. ^{1}H NMR spectrum of the product **11u**

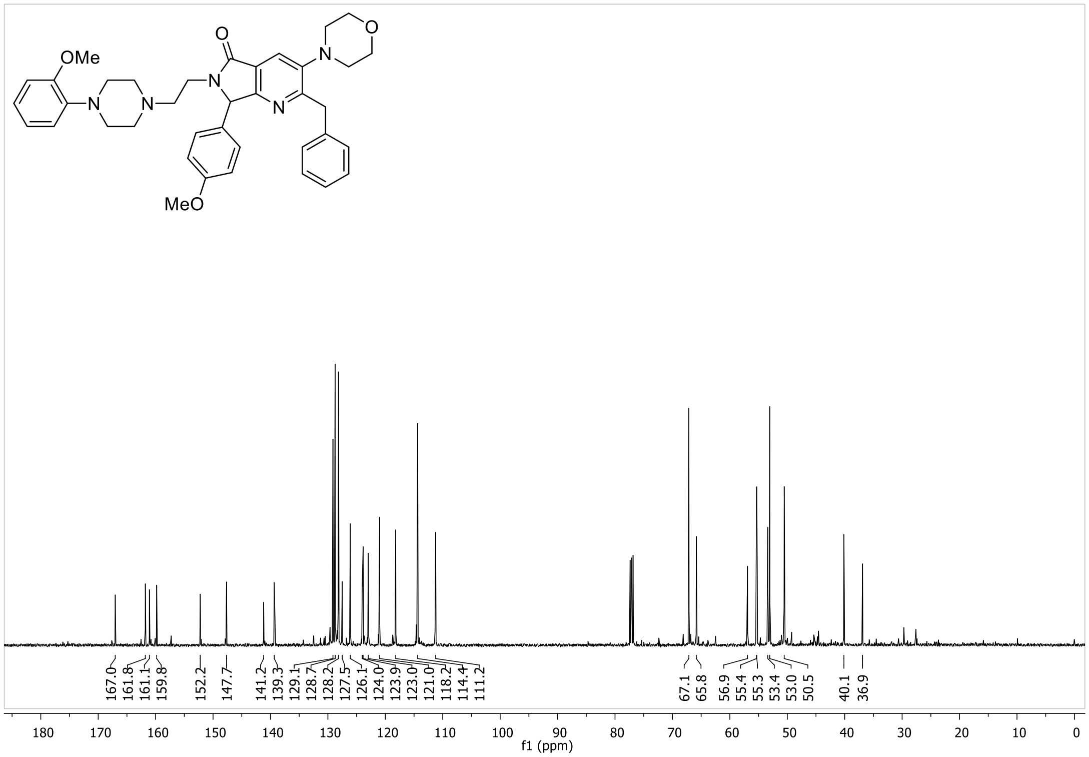

Figure S42. ^{13}C NMR spectrum of the product **11u**

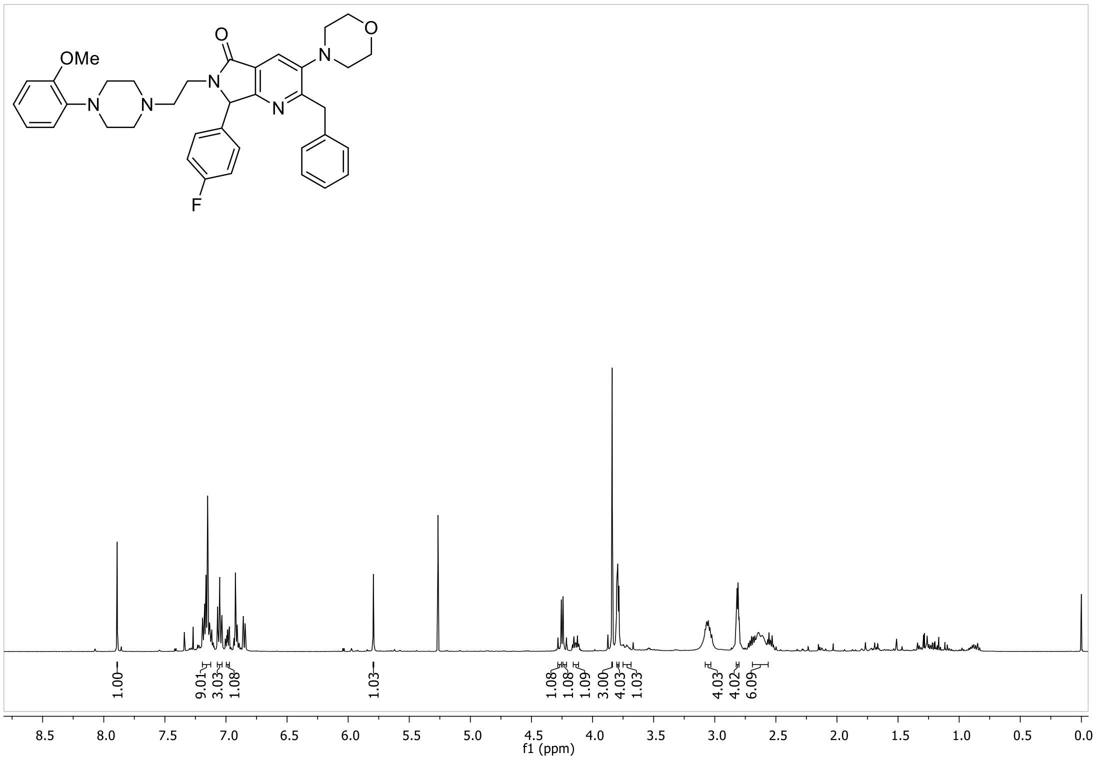

Figure S43. ^{1}H NMR spectrum of the product **11v**

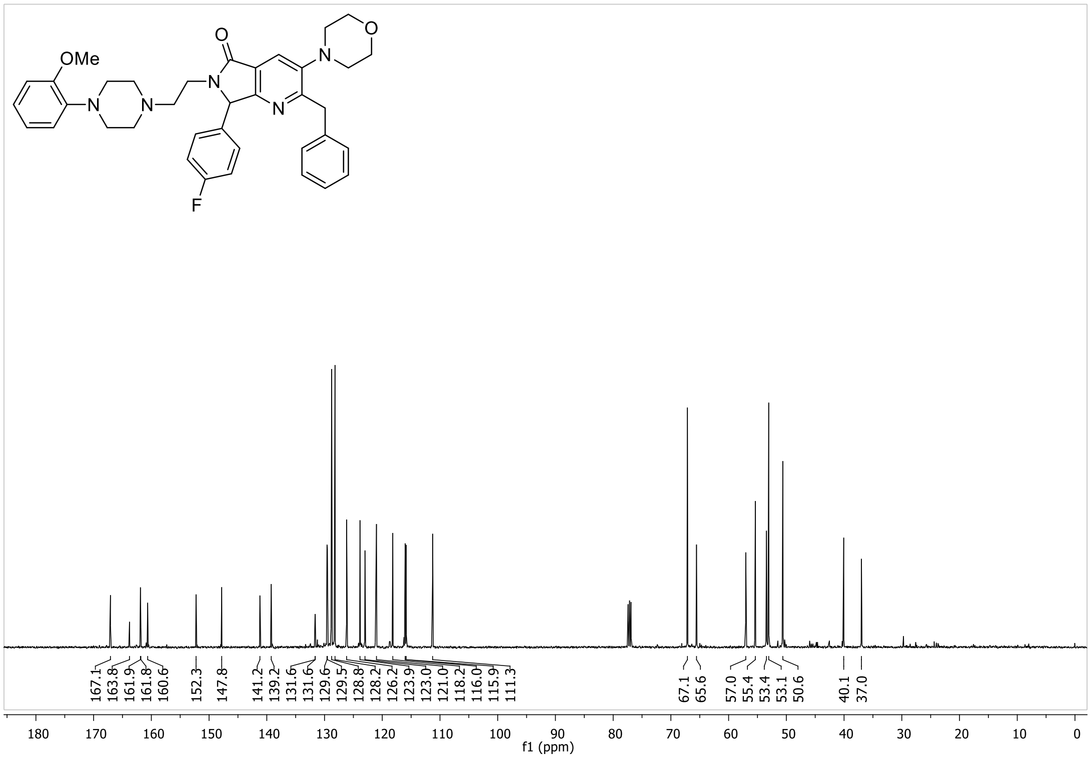

Figure S44. ^{13}C NMR spectrum of the product **11v**

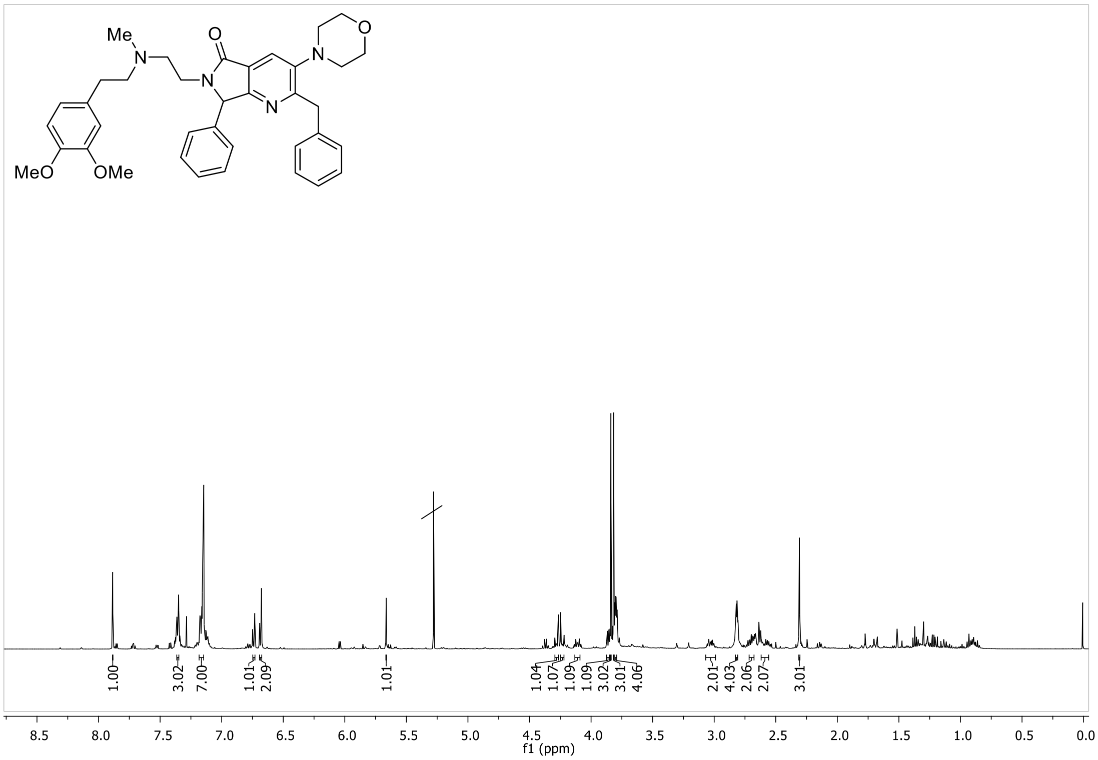

Figure S45. ^{1}H NMR spectrum of the product **11w**

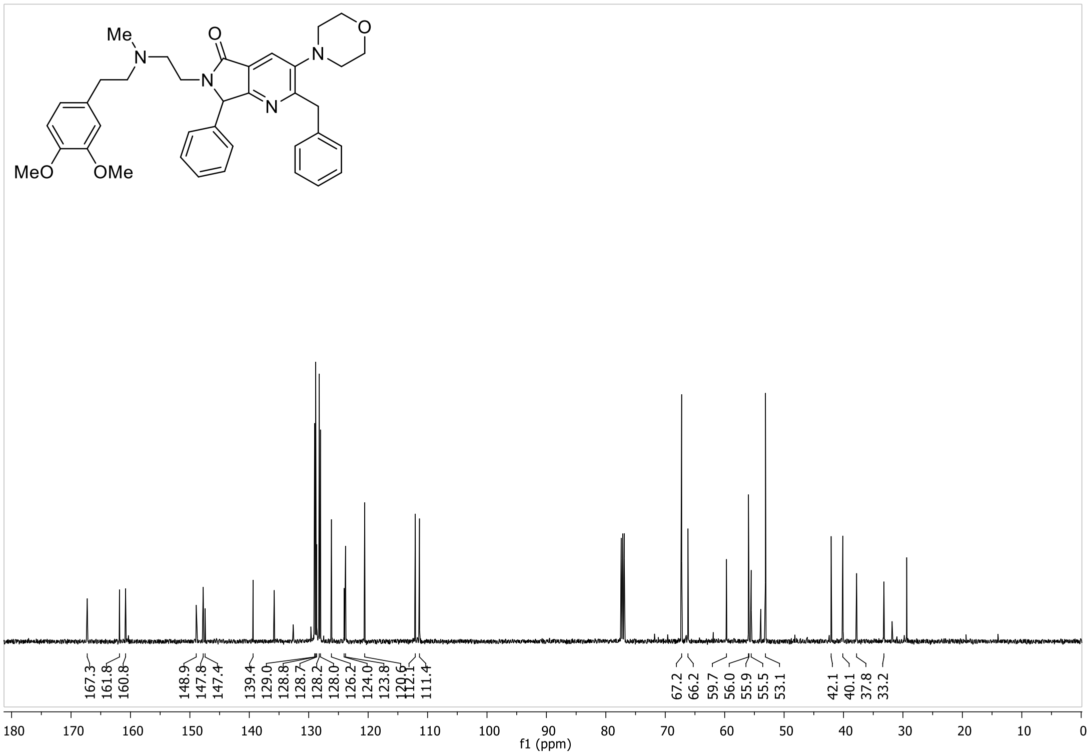

Figure S46. ^{13}C NMR spectrum of the product **11w**

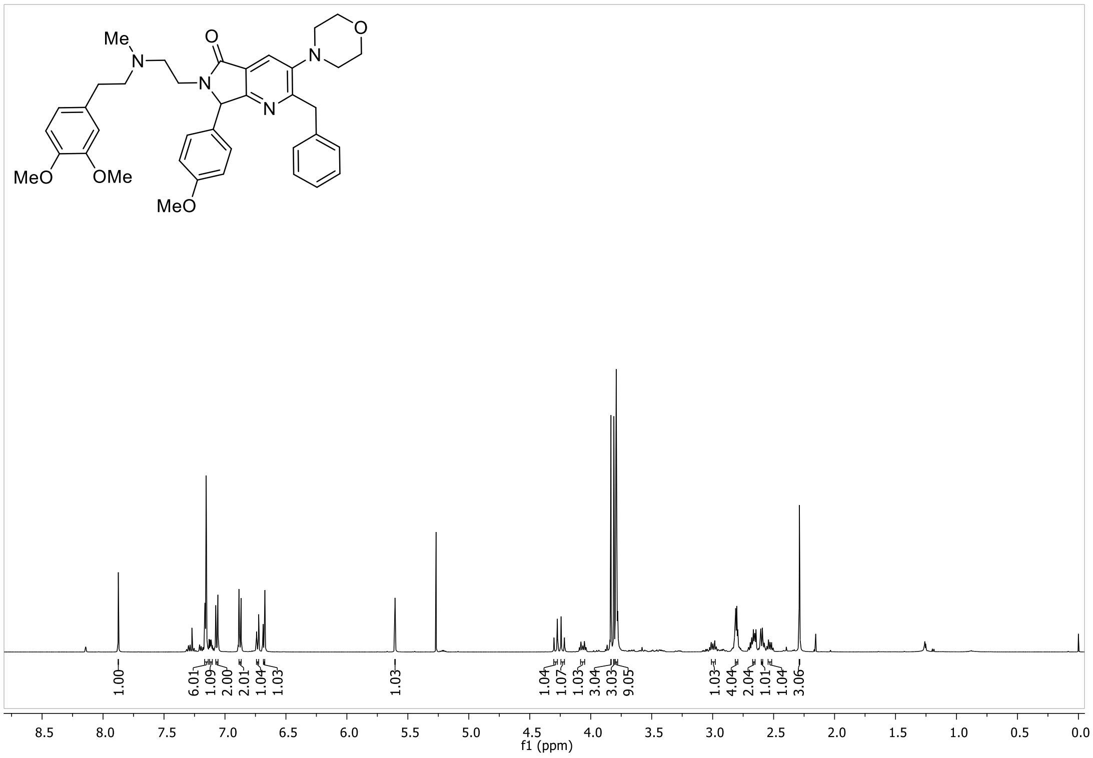

Figure S47. ^{1}H NMR spectrum of the product **11x**

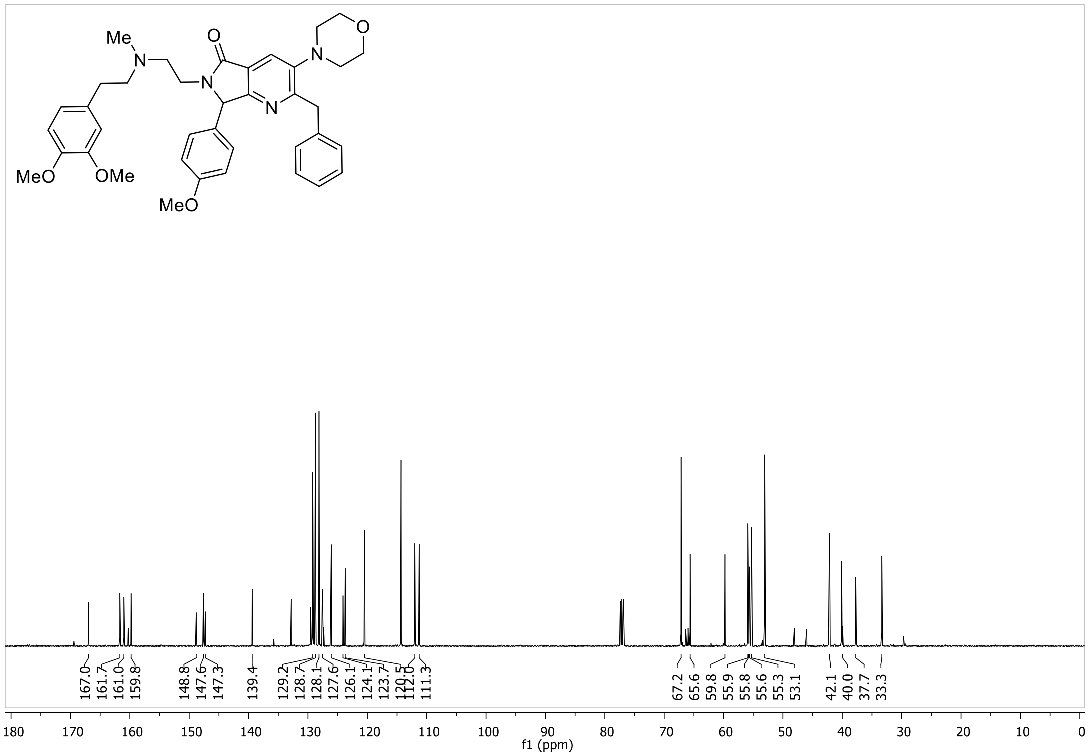

Figure S48. ^{13}C NMR spectrum of the product **11x**
